# Efficacy of supervised immersive virtual reality-based training for the treatment of chronic fatigue in post-COVID syndrome: study protocol for a double-blind randomized controlled trial (IFATICO Trial)

**DOI:** 10.1186/s13063-024-08032-w

**Published:** 2024-04-03

**Authors:** Jonas Tesarz, Hannah Lange, Marietta Kirchner, Axel Görlach, Wolfgang Eich, Hans-Christoph Friederich

**Affiliations:** 1 Department of General Internal Medicine and Psychosomatics, Heidelberg University, Im Neuenheimer Feld 410, 69120 Heidelberg, Germany, Germany; 2 DZPG (German Centre for Mental Health), Partner Site Heidelberg/ Mannheim/ Ulm, Heidelberg, Germany; 3 Institute of Medical Biometry, University of Heidelberg, Heidelberg, Germany

**Keywords:** Virtual Reality, Chronic fatigue, Exercise, Post-COVID, Immersive

## Abstract

**Background:**

The treatment of persistent fatigue after COVID-19 infection is complex. On the one hand, it involves maintaining a sufficient level of physical and mental activity to counteract possible degenerative processes of the body and nervous system. On the other hand, physical and mental activities can also lead to worsening of symptoms. Therefore, the challenge in treating Post-COVID fatigue is to stimulate the body and central nervous system in a way that stimulates growth and improvement, but does not overtax individual physical and mental limits. Special training programs try to take these characteristics into account, but often reach their limits. A promising approach is offered by new fitness technologies based on immersive virtual realities that stimulate both body and brain while minimizing physical and psychological stress. The aim of this study is to investigate the efficacy of supervised immersive Virtual Reality (VR)-based activity training compared to conventional activity training for patients with Post-COVID-associated fatigue.

**Methods:**

In a single centre, individually randomised, prospective, double-blind two-arm exploratory superiority trial with parallel group design, *N* = 100 patients with persistent fatigue after COVID-19 infection will be recruited. The intervention includes a supervised immersive neuromuscular training (12 sessions of 30 min over 6 weeks) based on a novel VR-exercise device. We will systematically compare the effects of this intervention on Post-COVID-associated fatigue with a supervised conventional activation program of comparable scope without an immersive environment. The primary outcome is the difference between groups in absolute change in the mean fatigue symptom severity measured on the Fatigue Severity Scale (FSS) from baseline to posttreatment assessment. Posttreatment assessment in both groups will be conducted by blinded outcome assessors. At three and six months afterwards, patients are sent self-report questionnaires for follow up. The main analysis will be based on the intention-to-treat principle.

**Discussion:**

To the best of our knowledge, this is the first exploratory study on a supervised immersive neuromuscular training for the treatment of persistent fatigue after COVID-19 infection.

**Trial registration:**

German register for clinical studies (ID: DRKS00032059)

Prospectively registered on June 16th 2023.

URL of trial registration:

**Supplementary Information:**

The online version contains supplementary material available at 10.1186/s13063-024-08032-w.

## Introduction

### Background and rationale

Worldwide, hundreds of millions of people have become infected with the novel virus during the SARS-CoV-2 pandemic. Some of those infected experience long-term symptoms that can last for weeks to months and are often referred to as "Long-COVID " or "Post-COVID syndrome" [1, 2]. According to current knowledge, the most common long-term symptoms are fatigue [3], poor concentration and shortness of breath, as well as a pronounced exercise intolerance ("Post-exertional malaise") [4, 5]. The symptom-cluster “fatigue” (including “chronic fatigue” and “rapid physical exhaustion”) is the most reported symptom cluster 6 to 12 months after acute infection with an estimated prevalence of 37, 2% [6].

Affected persons who previously had no complaints, both physically and psychologically, suddenly find themselves confronted with substantial performance losses in both their mental and physical abilities [4, 5]. Although the complaints are often substantial and can also be objectified in clinical examinations, the underlying mechanisms are still largely unknown. Accordingly, no causal therapeutic approaches have been developed so far [1].

The treatment of affected patients is often complex. It is recommended to maintain a sufficient level of physical and mental activity to counteract possible degenerative processes of the body and nervous system [1, 7, 8]. Newer therapeutic approaches attempt to address not only the musculoskeletal system and the cardiovascular system, but also the neuromuscular processes by including the brain and the nervous system as central elements of movement control in the training. At the same time, physical and mental activities can also lead to a worsening of symptoms [9]. There is a risk that the general condition and thus the quality of life of the patients will worsen in the long-term due to overstraining [9, 10].

Adequate pacing is therefore recommended to accompany activation [1]. Pacing is the strategy of remaining as far as possible below the physical, mental, emotional and social stress limit [8]. The central message of pacing is therefore to listen to one's own body and to stay within one's own energy limits. However, since even low levels of physical activity can overtax individual performance limits, an adequate dosage of exertion is often not possible within regular sports and fitness programs, and even specialized training programs often reach their limits.

Different training strategies have been proposed to overcome these difficulties [11]. One proven strategy are personalized training programs, in which individual exercise volumes and intensities are adapted to the personal needs and abilities of the affected person in a one-on-one setting. Other approaches emphasize the promotion of neuromuscular control and neurocognitive skills in movement performance. [12, 13] Such neuromuscular training approaches place an emphasis on improving proprioceptive skills, coordination, and neuromuscular gating [14, 15]. New fitness technologies based on immersive virtual realities that stimulate both body and brain while minimizing physical and mental stress offer a promising approach.

The ICAROS system is a special system that is increasingly being used in the rehabilitation and fitness sector [16, 17]. The ICAROS system is an immersive VR flight simulator that allows participants to fly through virtual worlds with full physical effort and experience them from a bird's eye view.

The virtual reality flight simulator is controlled by the participant's own body movement and thus conveys the feeling of flying. To promote participant motivation and engagement, the approach combines immersion with gamification, opening new challenges and game variations for participants. Patients with limited physical capacity can therefore experience joyful and “fatigue-free” physical activities linking the experience of joy to the experience to the experience of being physically active. ICAROS systems are used in sports, and rehabilitation training [18]. Initial studies have shown that ICAROS training programs simultaneously develop reflexes and coordination skills and train different muscle groups [19]. The brain can thus gradually relearn its capabilities without having to exceed individual limits. So far, however, little is known about the impact of "virtual reality"-based fitness applications on individuals with Post-COVID associated conditions.

## Objectives

The IFATICO randomised controlled trial (RCT) aims to investigate the efficacy of a supervised immersive VR-based neuromuscular activity training compared to a conventional non-immersive activity training of the same scope for patients with Post-COVID-associated fatigue. This paper presents the study protocol for the trial, adhering to the Standard Protocol Items: Recommendations for Interventional Trials (SPIRIT) Statement [20] (see Additional file 2 for the checklist) and reported in line with the SPIRIT 2013 Guidance for protocols of clinical trials [21], while the results of the trial will be reported in line with the CONSORT Statement for Randomized Trials of Nonpharmacologic Treatments [22].

The primary objective of the IFATICO trial is to study whether an immersive VR-based motor-sensory training is superior in treating patients with persistent fatigue symptomatology after COVID-19 infection compared to a conventional non-immersive training of the same extent according to the current standard recommendations of the WHO. The primary outcome is the difference between groups in absolute change in fatigue symptom severity from baseline to post-treatment. Secondary objectives are to: 1) test whether individuals in the intervention arm differ from individuals in the comparison arm in fatigue symptom severity at 3 and 6 months follow-up, burden of specific Post-COVID-associated complaints, quality of life, and global impression of change, and 2) to evaluate the effects on objective performance scores on the 6-min walk test and handgrip strength test, concentration ability, and memory performance, and 3) whether the intervention is safe and whether aversive side effects occur.

### Trial design

IFATICO is a single-centre, double-blind, randomized controlled exploratory superiority trial with two parallel arms. Participants will be randomised to the immersive VR intervention or the non-immersive trainings arm with 1:1 allocation, stratified by fatigue symptom severity (Fatigue Severity Score, FSS [23, 24]; three levels: mild, moderate, severe), and age (two levels: < 30 years vs. >  = 30 years).

## Methods

### Study setting

The main setting of this trial will be the Medical Clinic of the University Hospital Heidelberg in Germany. The University Medical Clinic of Heidelberg is a tertiary care hospital with facilities for highly differentiated diagnostics and treatments. For the supervised training sessions, the patient will visit the outpatient clinic of Heidelberg University Hospital. Here, the therapies for the two study arms will be performed in different areas to minimize the probability of an exchange between participants of different study arms.

### Eligibility

Patients who visit the outpatient clinic of the Department of General Internal Medicine and Psychosomatics will be made aware of the study either by their attending physician or by information in the waiting room. Patients will also be able to contact the study team through social media. Additionally, patients will be recruited on the level of primary healthcare (via teaching practices cooperating with the university) and secondary healthcare (via the Long-Covid Network Rhein-Neckar) to ensure a representative sample.

Eligibility for participation will be determined by telephone screening followed by in-person assessments. During the telephone screening, a trained staff member will ask patients whether they have a medical diagnosis of Post-COVID and suffer from fatigue, are available for the study and are able to reach the study location and complete questionnaires and computer tasks. Following this initial screening, the patients will be sent the baseline questionnaire assessing fatigue level, comorbidities as well as other information relevant for inclusion and baseline data for patient reported outcome measures. If no exclusion reason was present at this stage, participants will be invited to an additional face-to-face screening at the study institution. To ensure standardized assessment conditions, patients are instructed to avoid exhaustive activities for 72 h prior to assessment. At this time, patients will be evaluated for their physical and mental capabilities to ensure adequate exercise capacity for the study program (Post-COVID Scale of functional status (PCFS) of grade 3 or better) [25–27].

### Inclusion and exclusion criteria

#### Patients

The inclusion criteria require patients to 1) meet the WHO definition for this disease according to the WHO Case Definition 2021 [28]; [2] have fatigue symptoms that (i) occurred during the course of the COVID-19 infection and that either persist with symptom onset after the acute COVID-19 or its treatment, or that occur after the end of the acute phase but can be understood as a consequence of the SARS-CoV-2 infection [2] and (ii) have at least moderate severity, defined as an FSS-value of 36 or higher [23, 24] 3) agree to participate in the study by written informed consent, 4) be capable of giving consent, and 5) be 18 years or older.

The focus of this study is patients with persistent fatigue symptoms and exercise-induced insufficiency after COVID-19 infection who are significantly impaired in their participation in social and working life, rather than rehabilitation of patients that suffer from the consequences of a critical illness with intensive medical care. Therefore, we excluded patients who received intensive care for or since their COVID-19 disease and had "post-intensive care syndrome" (PICS) [6], patients who developed specific somatic sequelae such as cardiovascular complications with temporal latency as a consequence of COVID-19 disease.

Therefore, the exclusion criteria are as follows: 1) ICU stay since first COVID-19 infection, 2) need for more intensive medical management (e.g., grade 4 PCFS or severe comorbidities), 3) pre-existing medical conditions that are also associated with fatigue and by which the fatigue can be better explained than by Post-COVID syndrome (e.g. recurrent depressive disorder, ME/CFS), 3) inability to participate due to comorbid neurological or musculoskeletal conditions that result in moderate to severe physical disability or that would militate against VR exposure (eg. vertigo, significant hearing and/or visual impairment), 4) severe cognitive impairment or dementia, 5) known pregnancy, 6) insufficient German language skills, 7) participation in another COVID interventional study (observational studies are allowed), 8) weight over 130 kg (because the Icaros device is not suitable for patients with a higher weight) and 9) as a performance test, inability to reach the study location by using the staircase to the first floor (to ensure a minimal level of physical fitness).

Whether patients also fulfil the International Consensus Criteria (ICC) for ME/CFS [29] is queried and documented.

### Intervention arm

Dose, mode, and scheme of intervention.

The aim of this study is to investigate the efficacy of an outpatient supervised immersive neuromuscular training (12 sessions of 30 min each over 6 weeks) based on a novel VR training device, which is based on the principle of exergaming (from "exercise" and "gaming") and fosters neuromuscular control. The key element is the game-based animation of a virtual flight experience controlled by full-body movements.

Participants are placed in a special individually adjustable and movable medical training device (Icarus Health pro, CE MD Basic UDI-DI 426240612 0005D5), into which they lie almost horizontally in a `wingsuit position´ and rest on their forearms and lower legs and balancing the device in two axes. By shifting their center of gravity, they can control both the training device and virtual reality. The higher the degree of permitted deflection in these axes the more challenging the training gets. Additionally, patients receive visual information via a virtual reality headset, thus creating the illusion of flying over a landscape. The VR headset induces a dynamic flight experience that is closely coupled to the body movements in the ICAROS system. This creates the immersive feeling of "flying" for the participant. At the same time, the navigation requires the player to constantly control the body position and balance in space by appropriately tensing and loosening the arms, legs, and core muscles. For projecting the VR image, VR glasses (e.g., Samsung Gear glasses (HTC Vive Pro Virtual Reality Brille, Nr. 1668031) are used. The immersion in virtual reality makes it possible for patients with limited physical capacity to experience virtually "fatigue-free" joyful activities, such as flying through the mountains in a wingsuit or moving weightlessly through space (Table 1).

**Table 1:** Immersive neuromuscular training intervention

Session	Content
Opening sessions (Session 1–2)	▪ A detailed patient education will be performed to develop a better understanding of the links between fatigue and neuromuscular control ▪ The participant is introduced to the ICAROS training device and the different VR training programs. The patient is then asked to choose the VR training setting that suits him best (e.g., flight over the mountains) ▪ To accustom the patient to the VR experience and avoid adverse events, we will slowly increase the level of immersion. We will begin with combining the ICAROS device with a tablet and move on to a VR headset once the patient feels comfortable ▪ The control of the individual training intensity is done via the subjective rate of perceived exertion (RPE) on a standardized 10-point Borg-scale [8]. There are different exhaustion phases according to the WHO: 1) Preparation for return to exercise: RPE 0–1, 2) Low intensity activity: RPE 2–3, 3) Moderate intensity activity: RPE 4–5, 4) Moderate intensity exercises with coordination and functioning skills: RPE 5–7 and 5) Return to baseline exercise: RPE 8–10. Patients are asked, what level of exhaustion leads to worsening of symptoms in their daily lives. During our training sessions, we will stick to the exhaustion phase below If, for example, a subject experiences PEM when going on walks that lead to an exhaustion level of 6, we will choose phase 3) for this respective patient. During our training session we will train until he feels like he/ she has reached the exhaustion level of “5” on the 10-point Borg scale. This RPE monitoring [30, 31] allows us to control the patients' exercise intensity regardless of the cardiorespiratory exercise level, since patients with Post-COVID-associated fatigue have difficulty reaching the normally recommended heart rate due to fatigue and chronotropic incompetence [32] ▪ Within this training setting ("set") there is the possibility to repeat an individual number of training runs (“repetitions”: about 50–90 s). To determine the initial number of repetitions for a participant, the patient is asked to perform an immersive exercise as many times as is necessary until the subject reaches his or her individual determined exhaustion limit
Core sessions (Session 3–11)	▪ The immersive training programs are conducted under guidance. Participants can always ask questions and receive technical support in setting up the VR set and adjusting the ICAROS system. Before and after each session, the effect of the last session and the current session, respectively, is briefly discussed ▪ Participants can increase the difficulty or the number of ICAROS fights if they stay within their RPE-phase. If training does not lead to worsening of symptoms for at least seven days and patients feel ready to, they can proceed to the next higher exhaustion phase ▪ In those cases, in which an adjustment of the training intensity should become necessary due to subjective exhaustion or Post-exertional malaise, an adjustment of the extent and length is carried out according to the 5-phase model of the WHO recommendations [8]. That means we will return to a lower exhaustion phase ▪ In individual cases where patients arrive to the training session with an initial exhaustion level that is already above their RPE-phase, patients will be asked whether they want to do the training at all. If they want to, repetitions will be performed only on this exhaustion level and no further increase of exhaustion is allowed. In case patients do not feel fit to complete any training, the session will be rescheduled
Closing session (Session 12)	▪ During the last session the subjects are offered to compare themselves with the games from the first session and to reflect on possible changes on a voluntary basis ▪ Short feedback is given and ways to improve the sustainability of the progress made are discussed.

*VR* Virtual Reality*, RPE* subjective rate of perceived exhaustion*, WHO* World Health Organization*, PEM* Post-exertional malaise

### Comparison arm

Dose, mode, and scheme of intervention.

The control intervention is designed as a "golden standard treatment" for the treatment of Post-COVID-associated fatigue and consists of a supervised conventional activation program of comparable scope without an immersive environment based on the WHO recommendations for self-management after Covid-19-related illness [8]. Patients will be informed that two different activation programs will be tested against each other in the study. The trainers will describe the exercise program as a "first-line" exercise program for the treatment of Post-COVID-associated fatigue, which works by helping patients strengthen the body's neuromuscular control and thereby overcome fatigue. Initial patient education on the goals of the study, the principles of neuromuscular control to manage fatigue is kept the same between both study arms to minimize bias due to differences in expectations. The training program will be based on the general WHO guidelines for exercise management in Post-COVID-associated fatigue [8]. By the time of the study design, there were no generally accepted consensus recommendations outside of WHO recommendations, and only few papers had previously evaluated exercise prescription in detail, so only very general recommendations were available at baseline [33]. Similarly, few randomized clinical trials have been conducted on the safety and efficacy of different exercise programs in COVID-19 patients, and too few patients have been included to provide evidence-based recommendations. Chen et al. [34] published a systematic review and meta-analysis on the effect of pulmonary rehabilitation in patients with Post-COVID, which identified overall only three studies with a total of 233 patients. The treatment regimens studied were device-based respiratory training, coughing exercises, diaphragmatic training, and stretching exercises. Based on their literature review, Cattadori and colleagues proposed a concrete exercise protocol that is a combination of several recommended exercise programs [33]. However, all these recommendations have in common that they were derived from the rehabilitation field for the rehabilitation of patients with Post-COVID-associated symptoms after a severe initial course of infection (e.g., ICU stay), and that data on efficacy and safety have been lacking to date, especially in patients with an initially mild course. We therefore followed the WHO recommendations and modified them according to those currently published recommendations (Table 2).

**Table 2:** Non-immersive physical activity training (Control)

Session	Content
Opening sessions (Session 1–2)	▪ A detailed patient education will be performed to develop a better understanding of the links between fatigue and neuromuscular control ▪ The participant is introduced to the WHO physical activity training recommendations for managing fatigue ▪ The control of the individual training intensity is done via the subjective rate of perceived exertion (RPE) on a standardized 10-point Borg-scale [8]. There are different exhaustion phases according to the WHO: 1) Preparation for return to exercise: RPE 0–1, 2) Low intensity activity: RPE 2–3, 3) Moderate intensity activity: RPE 4–5, 4) Moderate intensity exercises with coordination and functioning skills: RPE 5–7 and 5) Return to baseline exercise: RPE 8–10. Patients are asked, what level of exhaustion leads to worsening of symptoms in their daily lives. During our training sessions, we will stick to the exhaustion phase below. If, for example, a subject experiences PEM when going on walks that lead to an exhaustion level of 6, we will choose phase 3) for this respective patient. During our training session we will train until he/ she feels like he/ she has reached the exhaustion level of “5” on the 10-point Borg scale. This RPE monitoring [30, 31] allows us to control the patients' exercise intensity regardless of the cardiorespiratory exercise level, since patients with Post-COVID-associated fatigue have difficulty reaching the normally recommended heart rate due to fatigue and chronotropic incompetence [32] ▪ The WHO recommendations for physical activity include a gradually increasing activity program based on the Borg scale of perceived exertion (RPE: 0–10) as explained above with respiratory exercises and stretching in the exhaustion phase 1) and specific resistance exercises (e.g., biceps curls, wall presses, arm raises, sit to stand, knee extensions, squats, and heel raises) and recommendations for independent cardiorespiratory activities (running, swimming, cycling, or dancing) in all following phases ▪ Subsequently, the patient is asked to choose the exercises that are most suitable for him/her (e.g. biceps curls, wall presses, arm raises and so on). During one training session he will complete two upper-body and two lower-body exercises. Within this training setting ("set"), there is the option to repeat an individual number of training runs (60–120 s) (repetitions). To determine the initial number of repetitions for a participant, the patient is asked to perform an exercise as many times as necessary until he/she reaches his/ her individual determined exhaustion limit ▪ In individual cases where patients arrive to the training session with an initial exhaustion level that is already above their RPE-phase, patients will be asked whether they want to do the training at all. If they want to, repetitions will be performed only on this exhaustion level and no further increase of exhaustion is allowed. In case patients do not feel fit to complete any training, the session will be rescheduled
Core sessions (Session 3–11)	▪ The WHO training programs are conducted under guidance. Participants can always ask questions and receive technical support in performing the exercises. Before and after each session, the effect of the last session and the current session, respectively, is briefly discussed ▪ Participants can increase the difficulty or the number of exercises if they stay within their RPE-phase. If training does not lead to worsening of symptoms for at least seven days and patients feel ready to, they can proceed to the next higher exhaustion phase ▪ In those cases, in which an adjustment of the training intensity should become necessary due to subjective exhaustion or Post-exertional Malaise, an adjustment of the extent and length is carried out according to the 5-phase model of the WHO recommendations [8]. That means we will return to a lower exhaustion phase
Closing session (Session 12)	▪ During the last session the subjects are offered to compare themselves with the games from the first session and to reflect on possible changes on a voluntary basis ▪ Short feedback is given and ways to improve the sustainability of the progress made are discussed

*RPE* subjective rate of perceived exhaustion*, WHO* World Health Organization*, PEM* Post-exertional malaise

#### Modifications

We anticipate no substantive modifications of the intervention, since it was piloted and subsequently adapted as part of a preceding feasibility study.

#### Concomitant care

The interventions are planned as an add-on to standard therapy. Prior to the trial, there are no restrictions regarding medication or other finished treatments. In both groups, participants may continue the treatment they received at baseline or start new therapies recommended by their treatment providers. Patients that participate in other intervention studies are however excluded from the study. For more detail, see exclusion criteria. Adjunctive treatment, as well as additional therapies started during the intervention, will be assessed using a self-report questionnaire after completion of therapy and at follow-up.

Additionally, we will instruct patients in both arms to include aerobic exercise in their daily lives, increasing the volume in short intervals of 30–60 s per session and working toward the WHO guidelines on physical activity (150-300 min of moderate-intensity or 75-150 min of vigorous-intensity physical activity per week) [20, 35].

In accordance with the “WHO support sheet for self-management after COVID-19-related-illness”, we will advise participants in both arms to drink plenty of water, wear loose and comfortable clothing and to eat at least one hour before the session. We will also warm-up before and cool-down after training. Regarding breathlessness, we will make sure that the patient can still speak a full sentence without pausing more than once or twice. If he or she cannot do so, we will go back to a lower level of exercise intensity [8].

#### Intervention integrity

We formulated the following core intervention components: 1) individual training sessions supervised by trained health specialists, 2) fixed dose of 2–3 training sessions per week of 30 min each for each patient over a period of approximately six weeks, 3) interventions focus primarily on (Arm 1:) practicing manualized physical activity in immersive virtual reality using the study training device (Icaros flight simulator); or (Arm 2:) practicing manualized physical activity training according to the current standard recommendations of the WHO for treating persistent fatigue after COVID-19 infection and 4) patients following the general instruction to include physical activity in their daily lives as far as possible.

Concerning intervention integrity, we consider criterion 1) to be fulfilled by the fact that all training sessions only take place under face-to-face supervision by a specialist. The two training programmes will be conducted by different personnel in order to maintain blinding and ensure treatment neutrality. Accordingly, specialists in the intervention group and comparison group will be different people who will be assigned their own patients and who will not switch between groups. The respective specialists (persons with a medical-therapeutic background, e.g. medical or psychology students or physiotherapists) will be trained by medical professionals on the respective training programme as well as on medical information about post-COVID, on the difficulties of implementing activation therapy in the treatment of post-COVID and on general issues in the treatment of patients with chronic diseases, and will be regularly supervised (2 × 2 h introductory training, and every 4 weeks—adjust so that it corresponds approximately to the truth). In the intervention arm, this specialist must have completed a training program by representatives of ICAROS ensuring safe and effective handling of the device. In the control arm, this specialist must have a completed training by study coordinates ensuring safe and effective execution of all exercises. Otherwise, the experts will not differ in the extent of their training and the information on the study between the two study arms. In the intervention arm, this specialist must have completed a training program by representatives of ICAROS ensuring safe and effective handling of the device. We consider criterion 2) to be met if a dose of a minimum of ten and a maximum of 18 training sessions (of a minimum of 15 and a maximum of 40 min) has taken place over a period of 4–8 weeks. We will assess the adherence to criterion 3) by requesting therapists in both groups to fill in a self-assessment questionnaire at the end of each training session. This documents the content performed (type of exercises, including intensity, time, and number of repeats with a minimum number of 1 repeat), as well as any deviations from the treatment manual (see additional files 4 and 5). In the intervention arm, patients will additionally fill out the “Igroup Presence Questionnaire” assessing level of immersion [36, 37]. Fidelity is met, if the level of immersion is above “0”. We consider criterion 4) to be met, if patients report in 70% or more session that they remembered to include physical activity in their daily lives since the last session.

For further reference on intervention integrity, please find the fidelity checklist attached as additional file 3. This fidelity-checklist will be used to ensure intervention integrity in both the VR- and the comparison group.

### Outcomes

We will collect patient-reported outcome measures at baseline just prior to randomisation, directly after intervention and at three- and six-months after intervention. These timepoints are frequently applied in RCTs for the treatment of fatigue in chronic conditions and will permit comparisons to be drawn with prior related trials (Heine et al., 2015).

#### Primary outcome

The primary outcome of this trial is the difference in groups in the absolute change in fatigue symptom severity (FSS) score from pre- (T_0_) to post-treatment (T_1_), measured after a standardized 72-h resting state (as defined above). The FSS is a widely used, well-validated clinical rating scale for fatigue symptomatology in chronic conditions [23, 24, 38, 39].

#### Secondary outcomes

Secondary outcomes include the difference in groups in absolute change in FSS score from T_0_ to follow-up at three (T_2_)- and six-months (T_3_) after end of intervention. At T_1_, as well as at T_2_ and T_3_, we will also calculate the absolute change from T_0_ in depressive and anxiety symptom severity (HADS) [40], Post-exertional Malaise (PEM) [41], Post-COVID-scale of functional status [25, 27], Sleep problems with the Jenkins sleep scale (JSS) [42], physical activity with the exercise vital sign [43], pain severity on the MPI-D [44], the Six-minute walk test distance [45] and the hand grip test [46], performance in the concentration and memory tasks, respectively along with burden of specific somatic complaints (Somatic Symptom Disorder–B Criteria Scale, SSD-12) [47, 48] and the Somatic-Symptom-Scale-8 (SSS8) [49], health-related quality of life (SF-12 questionnaire) [50], and EQ-5D 5L [51] and overall impression of change on the patients global impression of change and therapists global impression of change (PGIC, TGIC).

An additional secondary endpoint is the fatigue level responder status at T_1_. Patients with a reduction in fatigue severity of ≥ 15% are classified as responders. This cutoff is consistent with current recommendations for minimal important differences for fatigue patient reported outcome measures [52–54].

### Sample size calculation

Given that this is an explorative trial no formal sample size calculation was performed. However, based on recent data from an intervention study [55] in patients with Post-COVID-associated complaints, in which an FSS post-treatment of 5.0 (SD = 1.4) vs. 3.4 (SD = 1.7) between groups was observed, a mean difference of 0.9 (SD = 1.5) is assumed as a conservative effect considering that no active therapy was offered to the control group in this study. With a sample size of n = 45 per group 80% power is achieved to reject the null hypothesis of equal means assuming a mean difference of 0.9 (SD = 1.5) with a significance level of 5% using a two-sided two-sample t-test. Assuming a drop-out rate of 10%, n = 50 per group will be included. The sample size calculation is based on testing the primary hypothesis that immersive VR-based training therapy leads to a significantly different change in mean fatigue severity score (FSS) from baseline to follow-up assessment (T1) as compared to conventional training therapy in the comparison arm. A difference of 0.45–0.88 points on the FSS is considered clinically relevant [56]; we conservatively define the margin of 0.9 as the minimal clinically relevant difference for our trial. Based on previous post-COVID research [55] we assume a common standard deviation of 1.5 among FSS- fatigue scores.

With a sample size of 45 in the experimental group (immersive neuromuscular training) and 45 in the control group (non-immersive physical activity training), a two-sided test for the difference between two means with a 0.05 significance level has 80% power to detect a difference of 0.9 when the standard deviation is 1.5. Assuming a dropout rate of 10%, we will randomize 100 participants, 50 to each condition.

Sample size calculation was performed in PASS 16.0.12.

### Recruitment

**Fig. 1:**
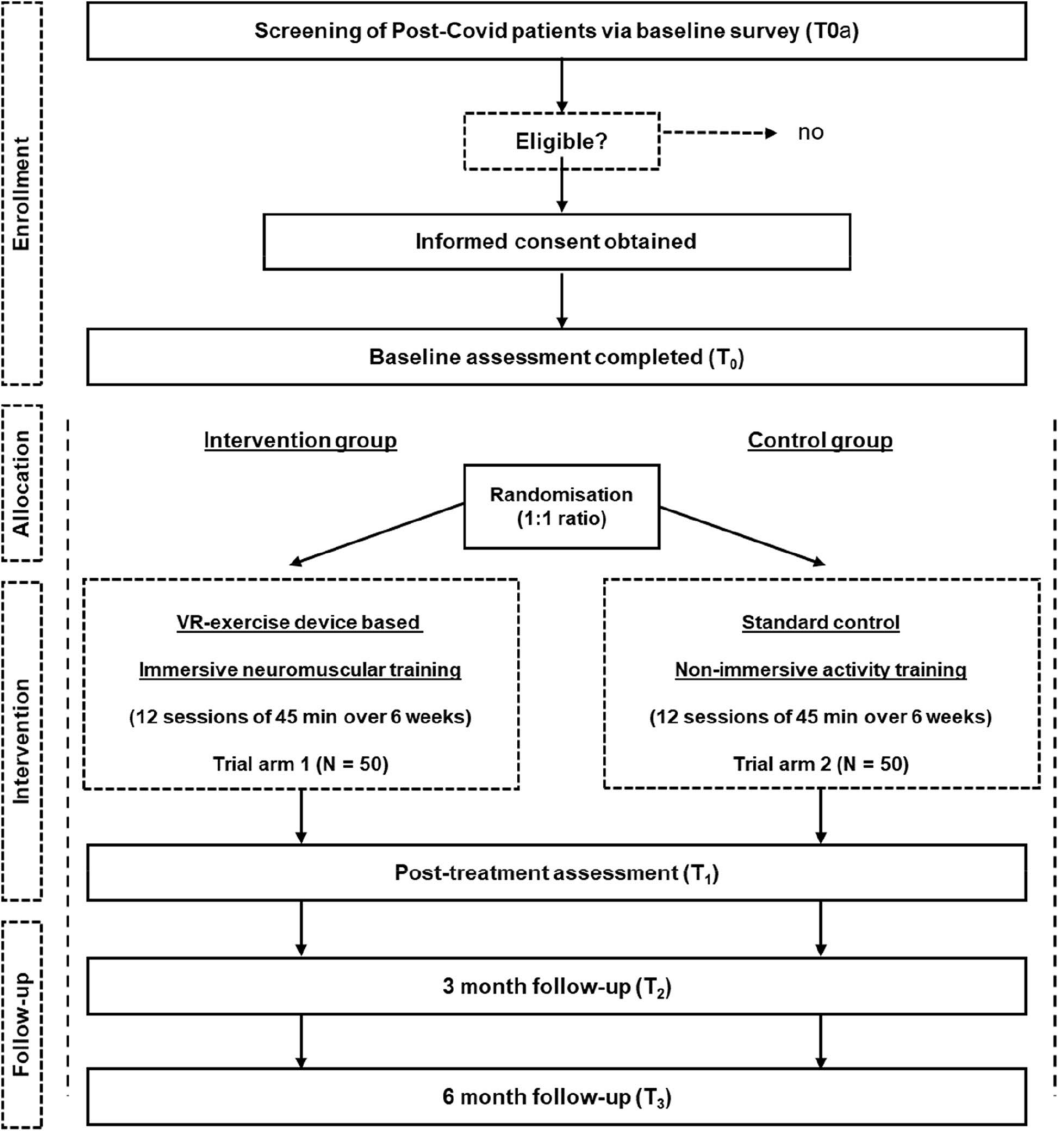
Study flow chart

In total, we plan to enrol 100 patients of whom approximately 50% will be allocated to the VR-based immersive neuromuscular training program. To ensure a timely recruitment and to recruit a broad and quite representative sample, recruitment is planned at three different levels (primary, secondary, and tertiary health care). Based on established research cooperations with university teaching practices of primary and secondary care as well as the Long-Covid Network Rhein-Neckar, an interdisciplinary network for the diagnosis and care of patients with Long-COVID complaints, we will be able to start implementation of screening and patient recruitment immediately with ethic approval (Fig. 1).

### Assignment of interventions

#### Allocation

To minimise reporting and selection bias, baseline measures will be collected just prior to randomisation. After giving written informed consent, eligible participants will then be randomly assigned (1:1) to the intervention or comparison arm via a secure web-based randomisation system (Sealed Envelope; https://www.sealedenvelope.com/) operated centrally by a data manager, not involved in the patient recruitment. Central randomisation will ensure concealment of the treatment sequence up to the allocation. The treatment sequence will be a computer-generated sequence of random numbers. Randomisation will be stratified by fatigue symptom severity (stratified by the FSS; three levels: mild, moderate, severe) and age (> 31, 31–50, < 50) using block randomization with variable block length.

#### Masking and patient information

To minimize potential bias due to expectancy and reporting bias, this study is randomized-controlled and blinded in accordance with current recommendations for conducting nonpharmacological trials [22, 57]. By withholding information about the exact content of the interventions and the hypothesis of our study, we attempt to provide the best alternative to a double-blind nonpharmacologic intervention study. Double-blinding encompasses firstly that subjects are not given insight into the content of the control study arm, and secondly that outcome assessors are not informed about what the study objective is and to which intervention arm participants are assigned. All outcome assessors are unaware of all patients group assignments. By also withholding information about the exact content of the interventions and the hypothesis of our study to patients, we attempt to provide the best alternative to a double-blind nonpharmacologic intervention study. Blinding of research staff conducting the intervention to this randomization is not possible. This approach is consistent with current principles for conducting nonpharmacologic intervention studies [58] and is now well established in larger randomized-controlled trials [59].

To minimize differences in expectations between intervention groups, standardized patient education will be provided regarding the exercise programs offered. In addition to the study procedure and the formal conditions of study participation, all participants will be informed in a standardized manner that they will 1.) participate in an individualized neuromuscular training program for the treatment of Post-COVID-associated complaints as part of this study, 2.) be randomly assigned, i.e., by chance, to one of two possible study arms as part of this study and, that 3.) these two study arms are similar in goal, scope, and intensity, but differ in terms of their technical implementation.

In this regard, participants will not receive any information about the specific content of the two treatment groups prior to participation. The patient information leaflet and educational discussion only state that the purpose of the study is to evaluate the effects of neuromuscular exercise therapy on fatigue by comparing two new outpatient exercise programs, without indicating that one of the programs is considered a control intervention (see additional file 6). After randomization, they will be informed about the content of the training program assigned to them via patient information leaflets (see additional files 7 and 8) and will not receive any information about the intervention in the other group. All researchers who assessed the outcomes or performed the data analyses will be masked as to group assignment. Patients will also be instructed not to discuss the content of their training program with other participant during the course of the study and may contact their trainer if they have any problems during study participation. In addition, we will instruct patients before the post-intervention interview not to mention which group, control, or intervention, they belonged to. In the case of unintentional unblinding during the assessment, the assessors will document how, and at which point the unblinding unfolded. Hence, we will be able to subsequently determine the extent to which blinded assessment was successful.

### Data collection

**Fig. 2:**
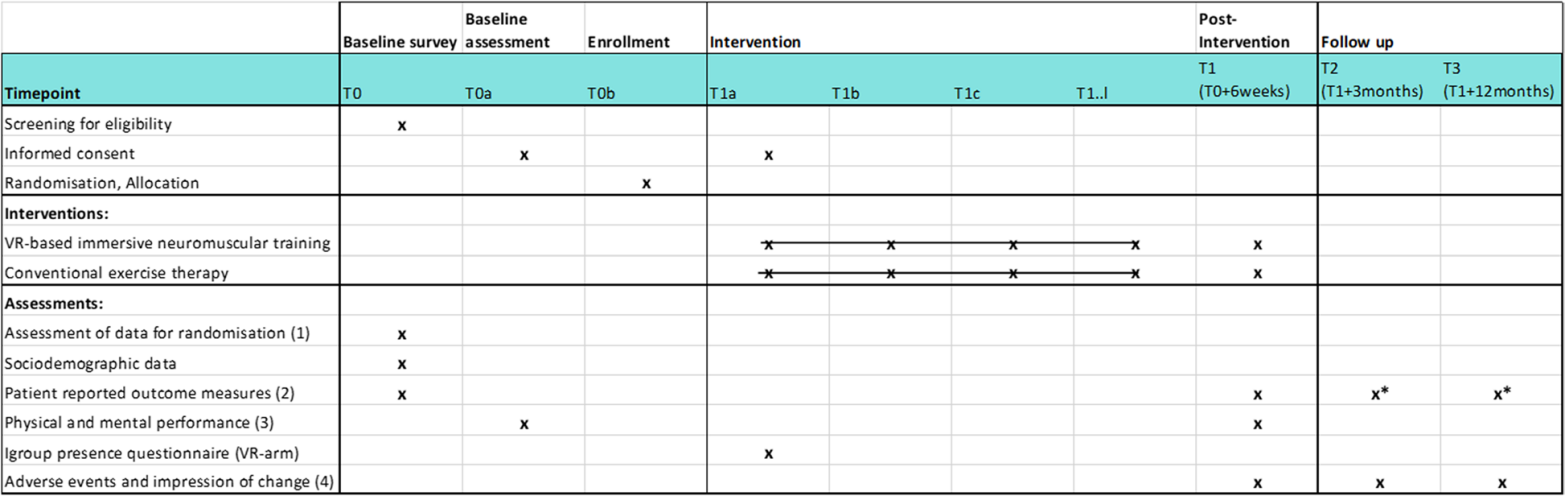
Study schedule. 1) Fatigue severity (FSS), age 2) PCSF*, FSS*, DSQ-PEM*, HADS, EQ-5D 5L, SSD-12, JSS*,* exercise vital sign*, SSS8, SSD12, SF12, MPI-D, Pain drawings, subjective theory for illness (items marked with * will also be assessed at follow up) 3) hand grip strength, 6 min walk test, *TMT, Stroop-test, N-back-test, Digit span-test* 4) TGIC (nur T1), PGIC, adverse events

We will collect participant data from intervention and comparison arms at baseline just prior to randomisation and at three- and six-months post intervention (see Fig. 2 for the study schedule). Informed verbal consent will be obtained from trial participants during our telephone screening by our study coordinates and informed written consent will be obtained by outcome assessors just prior to the pre-treatment assessment. Patients will be sent the consent form 24 h before giving verbal consent and therefore more than 24 h before giving written consent. We will use validated performance tasks and questionnaires and inform all participants that if they decide to withdraw from the study, the data already provided will be retained and used in the analyses unless they request otherwise and that a post-interventional assessment is planned even if they didn’t finish the study.

For the collection of patient-reported outcome measures as well as demographic and health data, we will use an electronic survey conducted in REDCAP. All data will automatically be updated to this database. For the collection of data from our assessments, we will use standardised paper-based checklists and sheets for all paper-based tasks. Data obtained from assessments will be manually uploaded to REDCAP. To ensure date quality, the data monitoring board will randomly choose patient files to assess whether data in REDCAP is identical to data in assessment-checklists and in sheets used for paper-based tasks.

### Data management

Responsibility for data management will be held by an independent data manager, which will not be involved in either the assessments or the delivery of therapies. Data are collected, managed, and stored in the central database under the supervision and responsibility of the data manager. The data manager ensures that all legal requirements for data protection are met and that data quality, sharing, and security (e.g., integrity of randomization) are maintained during all phases of the study. Data collected from patients after giving verbal but before giving written consent will be deleted if patients do not proceed to give written consent. In accordance with GCP guidelines, we ensure that all data and study documents are pseudonymized and retained for at least 15 years after study completion. All participants will receive a code number as a pseudonym and all records that contain names or other personal identifiers will be stored separately.

### Measures

At baseline, we will assess demographic and clinical characteristics, including age, gender, marital status, education level, employment status, physical health status, chronic medical disease, history of fatigue/depression/anxiety, current and past psychiatric treatment/psychotherapy, current and past psychopharmacological treatment, willingness to accept active training and willingness to accept psychosocial treatment. In addition, a specific `COVID-19 module´ is included that records information on the severity and course of acute COVID-19 infection, the development of Post-COVID symptoms after infection, and vaccination status.

Impairment of physical and mental abilities due to COVID-19 infection is classified using the Post-COVID-19 Functional Status Scale (PCFS) [25–27]. The PCFS focuses on assessing the limitations in daily living associated with persistent symptoms after COVID-19 infection. The scale covers the full spectrum of functional limitations from grade 0, "no functional limitations," to grade 4, "severe functional limitations," and grade 5, "death." Studies have demonstrated good construct validity of the PCFS scale in highly symptomatic adult subjects with confirmed and suspected COVID-19 infection three months after symptom onset [26].

Patient Reported Outcomes (PROMs):

- *Fatigue*: Fatigue symptom severity will be assessed at each timepoint using the German version of the Fatigue Severity Scale (FSS) [39]. The FSS is an established Patient Reported Outcome (PRO) instrument for assessing the severity of fatigue and its impact on a person's activities and lifestyle in patients with a variety of medical conditions [38]. The subject is asked to rate the severity of the fatigue symptoms experienced in the last week using a numeric scale ranging from 1 (strong disagreement with the statement) to 7 (strong agreement with the statement). It includes nine statements that describe the severity and impact of fatigue. FSS total scores are usually reported as the mean of the nine items (range 9—63); a higher score indicates higher fatigue severity. According to published cut-off scores for the FSS, a further distinction can be made between mild/no fatigue (FSS total score ≤ 35), moderate fatigue (36 ≤ FSS total score ≤ 52), and severe fatigue (FSS total score ≥ 53) [23, 24]. Acceptable reliability, internal consistency, sensitivity, and good responsiveness to change have been demonstrated for people with chronic fatigue in physical illness [38].
- *Post-exertional Malaise*: Presence and severity of PEM will be measured using the German version of the DePaul Symptom Questionnaire—Post-exertional Malaise (DSQ-PEM) [41, 60]. The first five items assess the frequency and severity of PEM over a six-month period. Frequency is rated on a 5-point Likert scale (0 = not at all, 1 = a little, 2 = about half the time, 3 = most of the time, and 4 = all of the time. Participants rated the severity of each symptom on a 5-point Likert scale: 0 = symptom absent, 1 = mild, 2 = moderate, 3 = severe, 4 = very severe). The DSQ-PEM has been shown to have good test–retest reliability, and these five DSQ PEM items have good internal reliability (α = 0.84) [61]. Three additional PEM items assess the duration of symptom worsening after physical and mental activity. Good test–retest reliability was found for these items on symptom exacerbation by physical activity (k = 0.84) and symptom exacerbation by mental activity (k = 0.74) [61]. The fourth additional PEM item assessed how quickly patients recover from activities typically undertaken by healthy individuals. This item has previously been shown to have good test–retest reliability (k = 0.88) [61]. The fifth additional PEM item assessed whether participants did not exercise because it worsened their symptoms. Good test–retest reliability was also demonstrated for this item (k = 0.79) [61]. A score above a threshold for one or more of the first five DSQ-PEM items is considered in level-1 assessment. A threshold score of 2 to 4 for frequency (half of the time, most of the time, or all of the time) combined with a score of 2 to 4 for severity (moderate, severe, or very severe) for the same item is indicative of PEM. This method is recommended by the Common Data Elements PEM Working Group of the National Institute of Neurological Disorders and Stroke (part of the National Institutes of Health) [62]. Level-2 assessment was developed to further operationalize the Common Data Elements recommendations [41] and includes additional questions related to rapid recovery, exacerbation of stress, and PEM duration. Item 7 or 8 must be answered "yes," and item 9 requires a response of ≥ 14 h [4].
- *Emotional Distress*: The level of anxiety and depression of the last week will be assessed via the Hospital Anxiety and Depression Scale (HADS). With 14 items, the HADS is a self-assessment scale that measures anxiety and depression through two subscales. Seven items for each subscale are rated by the patient on a 4-stage response format. It was particularly developed for somatic disorders and physical symptoms were therefore excluded. The HADS has high validity and reliability [40].
- *Health-related quality of life*: We will assess health-related quality of life using the SF-12 and EQ-5D 5L questionnaires [50, 51]. The SF-12 is a widely used general health questionnaire that consists of a mental component and a physical component and has demonstrated its psychometric robustness in numerous studies. Overall sum scores are in the range of 0 to 100 with higher scores indicating better quality of life. The EQ-5D 5L is a standardised instrument for measuring generic health status and comprises five dimensions: mobility, self-care, usual activities, pain/discomfort, and anxiety/depression [51]. The patient rates each dimension on 5 levels: no problems, slight problems, moderate problems, severe problems, and extreme problems. To calculate quality-adjusted life years, the EQ-5D preference weights which are EQ-5D health states evaluated with a German tariff are combined with time [63].
- *Global impression of change*: Global impression of change will be assessed from both the patient’s (patient’s global impression of change, PGIC) and the therapist’s´ perspective (therapist’s global impression of change, TGIC). It is a 7-point scale with answers coded from “very much improved” to “very much worse”.
- *Burden of somatic complaints:* We will measure burden of specific somatic complaints using the SSD-12 [47, 48] and the SSS8 [49].

The SSD-12 comprises 12 items that require respondents to rate how frequently they experience each cognition, emotion, or behaviour on a 5-point Likert scale (from 0 = “never” to 4 = “very often”). Overall sum scores are in the range of 0 to 48 and provide a three-dimensional measure of the psychological criteria of DSM-5 Somatic Symptom Disorder. The SSD-12 displays high internal consistency (Cronbach’s α = 0.94) and good convergent validity, correlating well with measures of somatoform complaints, depression, and anxiety.

*The SSS8 *[49] is a validated measure of somatic symptom burden. It contains 8 items, asking participants "During the past 7 days, how much have you been bothered by any of the following problems?" concerning gastrointestinal, pain, fatigue, and cardiopulmonary aspects. Items are scored on a 5-point Likert scale (0=Not at all, 1=A little bit, 2=Somewhat, 3=Quite a bit, 4=Very much). Somatic symptom scoring ranges from 0-32 [(no to minimal (0-3 points), low (4-7 points), medium (8-11 points), high (12-15 points), and very high (16-32 points)]. It has good sensitivity to change with a minimal clinically important difference (MCID) of 3-points [64].

The SSD-12 and the SSS8 in combination provide good diagnostic accuracy (SSS-8 + SSD-12: AUC = 0.79; 95% CI = 0.74-0.84) [65] for somatoform disorders.

- *The Exercise vital sign *[43] is a brief measure to estimate physical activity. It contains two items asking 1) “On average, how many days per week do you engage in moderate-to-vigorous physical activity (like a brisk walk)?” and 2) “On average, how many minutes do you engage in physical activity at this level?”. Since the most frequently used measure for physical activity, the International Physical Activity Questionnaire (IPAQ), is under criticism for not being a valid measure of physical activity [66] we found the exercise vital sign as a brief self-reported measure in combination with giving the patients pedometers to count steps in their daily lives the best fit for our study.
- *Sleep problems*: The Jenkins Sleep scale (JSS) [42] is a simple and non-time-consuming measure for the most common symptoms of sleep disorders. The instrument includes four items measuring quality of sleep over the preceding four weeks rated on a six-point Likert scale from 0) not at all to 5) 22–31 days a month. Items regard 1) trouble falling asleep, 2) trouble staying asleep, 3) frequent awakenings during the night, and 4) subjective feelings of fatigue and sleepiness despite having had a typical night’s rest. A cut off score of 2 accounts for sleep disturbances, correlating to at least one troubled night a week [67]. The JSS has good internal consistency (Cronbachs alpha: 0,8 [68]) as well as good construct and content validity and reliability [67–70].

#### Pain assessment

- *Pain severity*: Pain severity will be assessed via the pain severity subscale of the Multidimensional Pain Inventory (MPI-D) [44]. This subscale summarizes the items pain now, pain in the last week and suffering related to pain. The MPI-D is a valid instrument with a high reliability (Cronbach’s α ≥ 0.90).
- *Pain drawings*: Pain drawings will be used to quantify the spatial extent of pain. Drawings will be recorded on an Android device (Android Galaxy Tab S6) using a special stylus pen and the app "Squid" (version: 3.5.0). Participants are asked by the neurophysiological investigator to mark all painful areas of their body on a provided body figure (two schemes—front and back view). To ensure that patients did not focus only on their main pain, the instruction stated, "Mark all areas where you have pain (That is, all affected areas, not just the back!). Mark the overall area where the pain occurs." After completion the pain drawing, the participant and physician discuss it together to eliminate any misunderstandings. The created pain drawings are extracted as image files. The area of the extracted pain areas is calculated by counting the number of pixels and relating them to the area of the filled body template, resulting in percentages of spatial pain extent. Areas will be calculated using the Analyze function in ImageJ.

#### Physical performance

- *Six-minute walk test distance.* The participants’ aerobic performance capacity was assessed by submaximal exercise testing (6-min walk test distance; 6-MWTD) in which the walked distance in 6 min along an indoor flat 35-m corridor was calculated and interpreted (poor prognosis if the walked distance is 300 m or less) [45].
- *Hand grip test*: Muscle fatigue and fatigability will be assessed by ten repeat hand grips at maximum force. The HGT is performed twice, once at the beginning of the assessment (F_1_), and repeatedly after 60 min (F_2_). Based on these values we will calculate the Maximum Force (F_max_), Mean Force (F_mean_), as well as the Fatigue Ratio (F_max_/F_mean_) and the individual Recovery Rate (F_2mean_/F_1mean_). We will measure the hand grip strength (HSG) using a digital hand dynamometer (GRIPX B07RZWB57J). For this, participants must sit in an upright position and place the forearm of the dominant hand in full supination on a standard table. Before starting the measurement, all participants have the opportunity to pull the handle twice to familiarize themselves with the device. The grip is pulled with maximum force for three seconds, followed by a five-second relaxation period under the supervision of the study assistant. Within one session, this procedure is repeated ten times with the dominant hand. The participants are verbally motivated during the measurement to continue using their maximum force and to perform all repetitions. The dynamometer measured the highest value reached within three seconds (force measurement in kg). The mean grip strength of all ten trials will be calculated for baseline values (F_mean_). The test with the highest measured value from the ten repetitions is scored as the maximum force (_Fmax_). For quantifying the degree of fatigability will use the ratio of the maximum force and the mean force (F_max_/F_mean_) with higher values indicating stronger decrease of force within one session. For quantifying the recoverability, we will use the ratio of the mean force from the first testing and the second testing (F_2mean_ / F_1mean_) with lower values indicating impaired recovery [46].
- In order to obtain data about physical activity from everyday life and promote physical activity [71], the patients were given a pedometer (NAKOSITE LUX2433) to take home. The device counted the steps every day during the study and steps taken were read out and documented by therapists at each training session.

#### Mental performance

To assess neurocognitive function, we will employ several brief tests.

- *Trail Making Test*: The Trail Making Test (TMT) provides information on visual search, scanning, speed of processing, mental flexibility, and executive functions [72, 73]. The TMT consists of two parts. TMT-A requires an individual to draw lines sequentially connecting 25 encircled numbers distributed on a sheet of paper. Task requirements are similar for TMT-B except the person must alternate between numbers and letters (e.g., 1, A, 2, B, 3, C, etc.). The score on each part represents the amount of time required to complete the task.
- *Stroop-Test*: The Stroop-Test is a measure used in both—experimental and clinical purposes [74]. It assesses the ability to inhibit cognitive interference, which occurs when the processing of a stimulus feature affects the simultaneous processing of another attribute of the same stimulus. Subjects are required to read three different tables as fast as possible. Two of them represent the “congruous condition” in which participants are required to read names of colors (henceforth referred to as color-words) printed in black ink and name different color patches. Conversely, in the third table, named color-word condition, color-words are printed in an inconsistent color ink (for instance the word “red” is printed in green ink). Thus, in this incongruent condition, participants are required to name the color of the ink instead of reading the word. In other words, the participants are required to perform a less automated task (i.e., naming ink color) while inhibiting the interference arising from a more automated task (i.e., reading the word). Interference is expressed as the difference between the times on these two types of cards. Total time per card divided by number of stimuli on the card occasionally is used to estimate time per stimulus [75].
- *N-Back Task*: The N-Back task is a continuous performance task that is used to measure working memory [76]. In the N-Back task, participants are presented a sequence of stimuli one-by-one. For each stimulus, they need to decide if the current stimulus is the same as the one presented *N* trials ago. The *N* can be 1 trial, 2 trials, 3 trials, etc. [77].
- *Digit Span*: The Digit Span test is a subtest of the Wechsler Adult Intelligence Scale (WAIS) as well as of the Wechsler Memory Scales (WMS) [78, 79]. Subjects are asked to read a sequence of numbers and asked to repeat the same sequence back to the examiner in order (forward span) or in reverse order (backward span). Forward span evaluates the efficiency and capacity attention. Backward span is an executive task particularly dependent on working memory. The Digit Span subtest can be scored as one summary value (age-normed and contributing to summary scores in the Wechsler tests), or separately for forwards and backwards performance [78].

#### Treatment expectation and satisfaction, compliance, and acceptability

- *Treatment Expectation Questionnaire (TEX-Q):* The Treatment Expectations Questionnaire (TEX-Q) is a generic, multidimensional self-report scale for assessing patient expectations of medical and psychological treatments and allows comparison of the effects of multidimensional expectations across medical conditions [80].
- *Igroup Presence Questionnaire (IPQ)*: The Igroup Presence Questionnaire (IPQ) is a scale that measures the feeling of presence in a virtual environment (VE). The current version of the IPQ consists of three subscales and an additional general item that is not assigned to any subscale. The three subscales concern the following dimensions 1) Spatial Presence—the feeling of being physically present in the VE, 2) Involvement—measuring the attention paid to the VE and the involvement experienced and 3) Experienced Realism—measuring the subjective experience of realism in the VE. The additional general item assesses the overall "feeling of being there." [36, 37].
- *Severe adverse events (SAEs):* SAEs will be documented and reported descriptively.
- *Dropouts*: Dropouts will be analysed according to the underlying reason for dropout and distinction will be made between dropouts preventable and not preventable by modification of study design.

### Retention

We will continuously monitor the trial for any operational issues (i.e., failure in appointment management, no-show of patients). To encourage manageme at each study timepoint, non-responders will receive up to five reminders in total via phone, mail, and e-mail. These reminders will offer the option of being mailed a hard copy of the questionnaire to complete and return via reply paid envelope and/or filling in the FSS alone.

### Statistical methods

Descriptive statistics (absolute and relative frequencies for categorical variables and measures of position (mean, median) and variability (standard deviation, interquartile range, and range) for continuous variables) will be used to compare participant characteristics between the study arms at baseline. Additionally, baseline characteristics will be analysed in patients who discontinue the study.

The analysis of the primary outcome is conducted in the full analysis set following the intention-to-treat principle so that all randomised patients are analysed. Missing values will be imputed by multiple imputation with the fully-conditional specification method [81]. In a sensitivity analysis, the per-protocol population is analyzed, which includes only patients in whose cases the integrity of the intervention (criteria 1–4) is met. Additionally, a complete case analysis will be performed meaning that the analysis of the primary endpoint is repeated in the subset with no missing values for the primary endpoint or relevant covariates. In another sensitivity analysis, we will exclude unmasked patients from the analysis, to assess the impact of unmasking patients. Furthermore, to evaluate the potential influence of at-home aerobic training, we will conduct an additional sensitivity analysis. This will involve adjusting for at-home aerobic training as a co-variable to discern its impact on outcome variables. Lastly, we will execute another sensitivity analysis where patients who were unmasked are excluded, assessing the effect of unmasking on the results.

The null hypothesis for the primary outcome is: The mean absolute change in fatigue symptom severity from T0 to T1 is the same in both groups. The null hypothesis will be tested using a linear regression model at a significance level of 5%. In addition to the group variable, the regression model will contain the following variables at baseline: fatigue symptom severity and age (fixed effects). We will compute effect sizes and interpret them together with the respective 95% confidence intervals [82]. The analyses of the secondary outcomes will be purely exploratory and analogous to the analysis of the primary outcome. We will conduct subgroup analyses for the primary and secondary outcomes applying linear regression models, which will also contain an interaction term between the study arm (intervention vs. comparison) and the subgroup to be investigated. The corresponding *p*-values of these tests will be interpreted purely descriptively. The entire statistical evaluation will be performed in R (version 4.0.2 or higher) [83]. Prior to all analyses, we will pre-specify a statistical analysis plan.

#### Missing data

Applying the participant retention strategies outlined above, we will try to minimise the missing outcome data. Notwithstanding, we will record reasons participants are lost to follow-up.

### Monitoring

We will record all adverse events with respect to relation to study, severity, potential for the event to have been anticipated, and action taken. In the case of severe adverse events clearly related to the study intervention, the study director will be informed and, in consultation with the local ethics committee, will decide on the continuation of the study. No interim analysis for effectiveness will be performed.

An independent Data Safety and Monitoring Board (DSMB) will be established for this trial to ensure the ongoing safety and integrity of the research. The DSMB will conduct regular assessments of the study at key intervals (baseline, 50% and 75% enrolment and at the end of recruitment and testing (last patient out)). The study will be overseen by the Principal Investigator (J. Tesarz), without the formation of a formal Scientific Study Steering Committee. This information has been included in the manuscript for full reporting of study governance*.*

### Dissemination policy

Regardless of the magnitude or direction of effect, the results of this trial will be presented at relevant national and international conferences and as published articles in peer-reviewed journals. Publication of the study results will be based on the CONSORT-SPI 2018 statement for social and psychological interventions and the CONSORT Harms 2022 statement [84, 85] . All authors will contribute sufficiently to the manuscript to be included as authors. Due to local data protection regulations, data sets cannot be made publicly available.

## Discussion

The overall objective of the IFATICO study is to evaluate the efficacy and safety of immersive VR-based motor-sensory training in patients with persistent fatigue symptoms after COVID-19 infection. Although there are now a large number of studies on Post-COVID syndrome as well as Post-COVID-associated fatigue, the number of clinical trials on therapeutic interventions is small. This is consistent with the fact that, to date, few specific treatment approaches exist for the management of Post-COVID-associated symptoms. Therefore, most treatment recommendations are based on expert consensus and refer to mostly non-specific treatment strategies for the treatment of fatigue and impaired physical or mental performance in general. At the same time, however, it is currently completely unclear whether these treatment approaches can be transferred one-to-one to Post-COVID associated symptoms. Since physical activation has also been associated with symptom worsening in patients with chronic fatigue and Post-exertional malaise, concerns about the safety of traditional physical training and activation programs have been raised repeatedly. Studies that have investigated specific treatment programs in this patient group and that have assessed efficacy and safety are currently scarce. The IFATICO-Trail aims to fill this knowledge gap and expand the treatment spectrum for these patients.

### Trial status

At the time of submission, patient recruitment to IFATICO is ongoing. The anticipated study completion date is November 2024**.** This trial was prospectively registered on the German Registry for clinical trials (DRKS) with study ID: DRKS00032059 on 16.06.2023. Any significant modification of the protocol (for example changes in eligibility criteria or analyses) will be the subject of a protocol amendment and will be transmitted to the concerned parties (principal investigator, outcome assessors, treatment providers and participants). If applicable, the protocol will be updated in the German registry for Clinical trials.

### Supplementary Information

**Additional file 1.** Ethical approval document.

**Additional file 2.** Standard Protocol Items: Recommendations for Interventional Trials (SPIRIT) checklist.

**Additional file 3.** IFATICO-Fidelity-checklist.

**Additional file 4.** SOP: Training manual intervention group.

**Additional file 5.** SOP: Training manual control group.

**Additional file 6.** Patient information before randomisation.

**Additional file 7.** Patient information after randomisation in intervention group.

**Additional file 8.** Patient information after randomisation in control group.

**Additional file 9.** CONSORT-Statement for Randomized Trials of Nonpharmacologic treatments (2017).

## Data Availability

Not applicable.

## Abbreviations

DSQ-PEM: De Paul Symptom Questionnaire for Post-exertional Malaise
EQ-5D-5L: EuroQol Research Foundation 5-level version
FSS: Fatigue Severity Scale
HADS: Hospital Anxiety and Depression Scale
ITT: Intention-to-treat
JSS: Jenkins Sleep Scale
MPI-D: Multidimensional Pain Inventory, deutsche Version
PCFS: Post-Covid Scale of functional status
PGIC: Patient’s global impression of change
TGIC: Therapist’s global impression of change
RCT: Randomized controlled trial
SF-12: Short-Form-Health Survey 12
SPIRIT: Standard Protocol Items: Recommendations for Interventional Trials
SSD12: Somatic Symptom Disorder-B Criteria Scale
SSS8: Somatic Symptom Scale 8
