# Supplementary material for: Efficacy of supervised immersive virtual reality-based training for the treatment of chronic fatigue in post-COVID syndrome: study protocol for a double-blind randomized controlled trial (IFATICO Trial)
Source: Trials. 2024 Apr 3;25:232. doi: 10.1186/s13063-024-08032-w (PMC10993519; doi:10.1186/s13063-024-08032-w)
Supplement: Supplementary file 3 — Additional file 3. IFATICO-Fidelity-checklist. [file 13063_2024_8032_MOESM3_ESM.docx]

# IFATICO- Fidelity Checklist (1, 2)

## Strategies used to improve fidelity (planned)

**Training of providers**

All staff delivering VR-therapy was initially trained by representatives of ICAROS GmBH ensuring safe and effective handling of the device. Delivery of training sessions in both intervention and control groups was rehearsed using role play and peer review techniques. Booster sessions each month will be included to increase adherence to treatment manuals and providers will be subsequently examined by the study director.

**Delivery of the intervention**

All trainers will adhere to a treatment manual to provide standardized training sessions that was tested thoroughly during our pilot trial considering feasibility and suitability to patient needs. Therapists are requested to fill in a self-assessment questionnaire at the end of each training session, documenting the exercises performed (including intensity, time, and number of repeats), as well as any deviations from the treatment manual.

Patients in the intervention arm of the study will be asked to fill out the Igroup-presence questionnaire to assess the level of immersion.

In case of cancellation of training sessions therapists will try to reschedule the appointment without further complications and offer alternative appointments on weekends in case patients are not easily available during the week because of pacing.

**Adherence to intervention**

To further increase adherence to intervention, we will ask patients to which extent they followed our instructions to include aerobic exercises in their daily lives whenever possible and remind them of the benefits of doing so.

**Data collection**

Before and after the intervention and at follow up, patients will have the opportunity to choose between a hard copy of the assessment questionnaire per mail and an online link, to increase participation. Questionnaires at follow up will be short (10min) to minimize missing data due to loss to follow up.

To minimize no-show or drop-out of patients, non-responders will receive up to five reminders via phone, mail, and e-mail.

## Extent, to which fidelity criteria were met (actually)

**Training of providers**

- All training sessions will be delivered by trained coaches.

**Delivery of the intervention**

- Igroup Presence questionnaire
- Answers on the Likert scale are rated from -3 to 3 from not immersive at all to very immersive. The scores on the different subscales are documented.
- The level of immersion will be assessed once after the first fully immersive flight.

| Objective | Recording |  |  |  |
| --- | --- | --- | --- | --- |
|  | <0 | >0 | >14 | >28 |
| Spatial presence |  |  |  |  |
| Involvement |  |  |  |  |
| Experienced Realism |  |  |  |  |
| Feeling of being there |  |  |  |  |

- Fidelity criterion is met, if immersion levels are over a score of zero. Mean immersion level and quartiles will be reported.
- Frequency and duration of training sessions
- Number of training sessions divided by the intended number of 12 training sessions is documented as a percentage.
- Duration of training sessions divided by the intended duration of 45minutes is documented as a percentage.
- Time period over which training sessions took place is divided by the intended time period of 6 weeks and is documented as a percentage.
- The Frequency and Duration of Training sessions will be assessed after the last training session took place.

| Objective | Recording |  |  |
| --- | --- | --- | --- |
|  | <83% (10ses.) | 83-150% (10-18ses.) | >150% (>18ses.) |
| Number of training sessions |  |  |  |
|  | <66% (30min) | 66-133% (30-60min) | >133% (>60min) |
| Duration of training sessions |  |  |  |
|  | <66% (<4weeks) | 66-133% (4-8weeks) | >133% (>8weeks) |
| Time period, in which all sessions took place |  |  |  |

- Fidelity criteria are met if a dose of a minimum of ten and a maximum of 14training sessions (of a minimum of 30 and a maximum of 60 minutes) has taken place over a period of 4-8 weeks.
- Deviations from training manual
- Deviations from training manual will be assessed after every training session.

**Intervention arm:**

| Objective/ Activity | Recording |  |
| --- | --- | --- |
|  | + | - |
| Instructor welcomed the patient to the session |  |  |
| (Opening session) Instructor educated the patient about risks and benefits of exercise therapy and the suggestions made by the WHO |  |  |
| (Opening session) Instructor explained the safe use of ICAROS Health including how to mount and dismount the device |  |  |
| (Opening session) Instructor let the patient choose a game |  |  |
| (Opening session) Instructor let the patient perform Icaros flights with low difficulty until the upper limit of his or her RPE-level was reached and documented the number of repetitions as a baseline |  |  |
| Instructor asked, whether the last training session improved or worsened symptoms |  |  |
| Instructor assessed the individual RPE level of the patient correctly and trained accordingly (x2) |  |  |
| Instructor ensured that the session could be paused or stopped at any time the patient felt uncomfortable and that the patient would not be left alone |  |  |
| Patient performed at least four of six exercises for warm up |  |  |
| Patient performed at least one repetitions on the Icaros-device |  |  |
| *Patient performed Icaros flights and was able to maintain or rise the difficulty* |  |  |
| *Patient performed Icaros flights and was able to maintain or rise the number of repetitions* |  |  |
| Instructor offered help when needed during the performance of Icaros flights and positively reassured patients |  |  |
| Patient performed at least four of six warm-up exercises again |  |  |
| Patient performed at least three of five cooling-down exercises |  |  |
| Instructor gave constructive feedback |  |  |
| Instructor answered any open questions about the intervention/ study |  |  |
| (Closing session) Patient was given the opportunity to give feedback about the study and his or her experience and is welcomed to reflect on his or her progress |  |  |

**Comparison arm:**

| Objective/ Activity | Recording |  |
| --- | --- | --- |
|  | + | - |
| Instructor welcomed the patient to the session |  |  |
| (Opening session) Instructor educated the patient about risks and benefits of exercise therapy and the suggestions made by the WHO |  |  |
| (Opening session) Instructor explained the exercise and performed every exercise once as instruction |  |  |
| (Opening session) Instructor let the patient perform every exercise until the upper limit of his or her RPE-level was reached and documented the number of repetitions as a baseline, divided them by three and rounded off |  |  |
| Instructor asked, whether the last training session improved or worsened symptoms |  |  |
| Instructor assessed the individual RPE level of the patient correctly and trained accordingly (x2) |  |  |
| Instructor ensured that the session could be paused or stopped at any time the patient felt uncomfortable and that the patient would not be left alone |  |  |
| Patient performed at least four of six exercises for warm up |  |  |
| Patient performed at least one exercise |  |  |
| Patient performed two exercises for the upper body |  |  |
| *Patient was able to maintain or rise number of repetitions of upper-body exercise* |  |  |
| *Patient reached ten repetitions of both exercises; the optimal number of repetitions suggested by the WHO* |  |  |
| *Patient was able to maintain or rise difficulty of upper-body exercises* |  |  |
| Patient performed two exercises for the lower body |  |  |
| *Patient was able to maintain or rise number of repetitions of lower-body exercise* |  |  |
| *Patient reached ten repetitions of both exercises; the optimal number of repetitions suggested by the WHO* |  |  |
| *Patient was able to maintain or rise difficulty of lower-body exercises* |  |  |
| Instructor offered help when needed during the performance of exercises and positively reassured patients |  |  |
| Patient performed at least four of six warm-up exercises again |  |  |
| Patient performed at least three of five cooling-down exercises |  |  |
| Instructor gave constructive feedback |  |  |
| Instructor answered any open questions about the intervention/ study |  |  |
| (Closing session) Patient was given the opportunity to give feedback about the study and his or her experience and is welcomed to reflect on his or her progress |  |  |

- Fidelity to treatment manual will be scored as number of items that were recorded as “+” divided by number of all items in percent. The item “Instructor assessed the individual RPE level of the patient correctly and trained accordingly” will be counted twice to highlight its importance. At the end of the trial, the mean percentage is determined for every patient. Fidelity criteria are met, if the mean percentage is 80% or higher and partly met if the mean percentage is between 50 and 80%.

**Adherence to intervention**

- Whether patients remembered to include exercise in their lives will be assessed before every training session.

| Objective | Recording |  |  |
| --- | --- | --- | --- |
|  | + | - | N/A |
| Aerobic exercise was included in daily life |  |  |  |

- The fidelity criterion for adherence to intervention is met, if patients answer with “yes” in 70% or more of the times asked whether they remembered to include aerobic exercise in their daily lives as far as possible.

## References

1. Hoffmann TC, Glasziou PP, Boutron I, Milne R, Perera R, Moher D, et al. Better reporting of interventions: template for intervention description and replication (TIDieR) checklist and guide. BMJ. 2014;348:g1687.

2. Bellg AJ, Borrelli B, Resnick B, Hecht J, Minicucci DS, Ory M, et al. Enhancing treatment fidelity in health behavior change studies: best practices and recommendations from the NIH Behavior Change Consortium. Health Psychol. 2004;23(5):443-51.
