# Supplementary material for: Efficacy of supervised immersive virtual reality-based training for the treatment of chronic fatigue in post-COVID syndrome: study protocol for a double-blind randomized controlled trial (IFATICO Trial)
Source: Trials. 2024 Apr 3;25:232. doi: 10.1186/s13063-024-08032-w (PMC10993519; doi:10.1186/s13063-024-08032-w)
Supplement: Supplementary file 4 — Additional file 4. SOP: Training manual intervention group. [file 13063_2024_8032_MOESM4_ESM.docx]

**Manual IFATICO Interventionsgruppe**

Inhalt

[Opening session 2](#_Toc156384746)

[Ablaufplan 2](#_Toc156384747)

[2) Aufklärung 2](#_Toc156384748)

[Spiele auf dem ICAROS-Gerät 3](#_Toc156384749)

[3) Aufwärmen 7](#_Toc156384750)

[4) RPE-Ausgangswert 8](#_Toc156384751)

[5) Cooling Down 9](#_Toc156384752)

[6) Closure 10](#_Toc156384753)

[Core sessions 11](#_Toc156384754)

[Ablaufplan 11](#_Toc156384755)

[2) Auswirkungen des letzten Trainings 11](#_Toc156384756)

[3) Aufwärmen 11](#_Toc156384757)

[4) Training 11](#_Toc156384758)

[5) Cooling Down 11](#_Toc156384759)

[6) Closure 12](#_Toc156384760)

[Closing session 13](#_Toc156384761)

[Ablaufplan 13](#_Toc156384762)

[2) Auswirkungen des letzten Trainings 13](#_Toc156384763)

[3) Aufwärmen 13](#_Toc156384764)

[4) Training 13](#_Toc156384765)

[5) Cooling Down 13](#_Toc156384766)

[6) Closure 14](#_Toc156384767)

Opening session

Ablaufplan

- 1. Ankommen: **5min**
- 2. Aufklärung, Erklären zum Icaros-Gerät, Aussuchen eines Spiels, Zuordnen zu einer RPE-Phase: **10 min**
- 3. Aufwärmen: **5min**
- 4. Ermitteln eines Ausgangswert für die Anzahl an Wiederholungen: Spielen von Icaros-Tablet-Spielen (je 50-90s) bis die RPE-Obergrenze der jeweiligen Phase erreicht ist (+Dokumentation): **10 min**
- 5. Cooling-Down: **5min**
- 6. Klären von Fragen, Verabschieden: **5min**
- Puffer: **5min**
- Gesamt: **45min**

2) Aufklärung

- Beziehung von neuromuskulärer Kontrolle und Fatigue
- Risiken bei VR (Schwindel, Übelkeit)
- Ängste und Unsicherheiten des Probanden explorieren und reduzieren

**Erklärung zum Icaros-Gerät**

- Patienten können jederzeit pausieren oder unterbrechen, wenn es Ihnen nicht gut geht
- Wir beginnen mit Spielen am Tablet und erst wenn sich die Patienten wohlfühlen, bekommen sie die VR-Brille.
- Ein Spiel/ Flug dauert 50-90s, danach wird jeweils kurz das RPE-Level erfragt, wenn die Patienten währenddessen bereits an die RPE-Obergrenze ihrer jeweiligen Phase stoßen, dann wird die Session beendet und die nächste Session mit einem Flug weniger gestartet.
- Erklärungen zum Einstieg auf das Gerät und Abstieg und Versicherung, dass man die ganze Zeit nebendran steht.
- Noch bestehende Ängste explorieren und positive Einstellung zum Trainingsprogramm evaluieren und ggf. stärken

# Spiele auf dem ICAROS-Gerät

Das Eintauchen in die virtuelle Realität ermöglicht es Patienten mit eingeschränkter körperlicher Leistungsfähigkeit, nahezu "ermüdungsfrei" freudige Aktivitäten zu erleben, wie z.B. in einem Wingsuit durch die Berge zu fliegen oder sich schwerelos durch den Raum zu bewegen.

Das ICAROS-Gerät wird mit zahlreichen "Spielen" geliefert, aus denen man wählen kann:


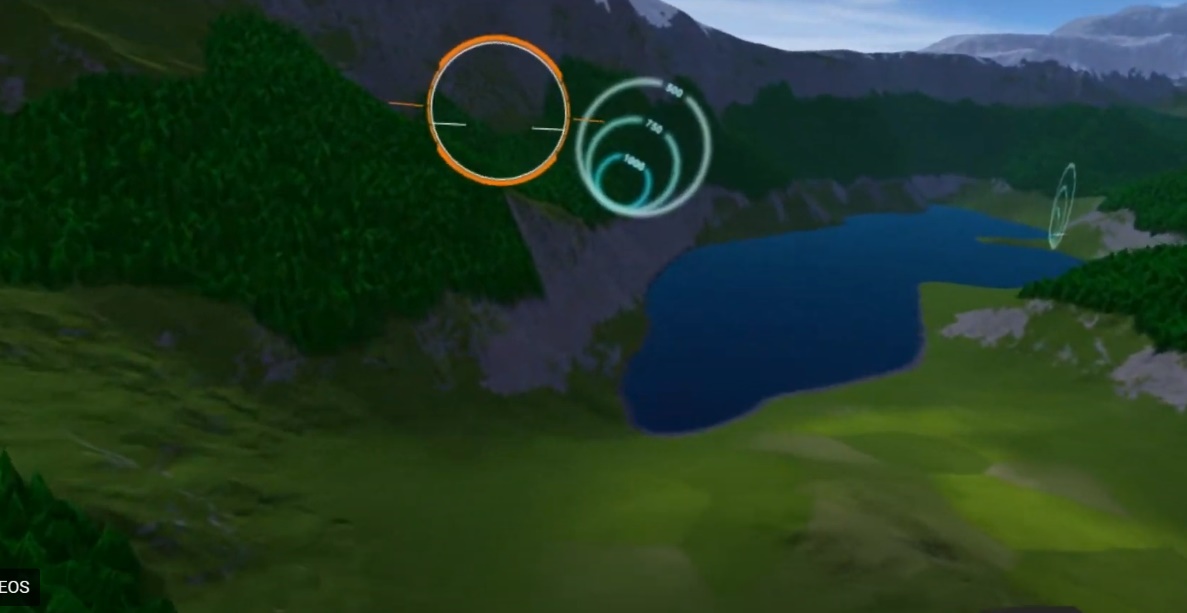
- Icaros Aim", bei dem man durch Berge fliegt, entweder ohne zusätzliche Herausforderung oder mit Ringen als Ziel, durch die man fliegen soll oder mit Raumschiffen auf die man schießen muss.

- "Deep", in dem man unter Wasser taucht, entweder ohne zusätzliche Herausforderung oder mit Ringen als Ziel, durch die man fliegen soll, oder zum Tiere suchen und sammeln oder zum kämpfen gegen ein U-Boot.


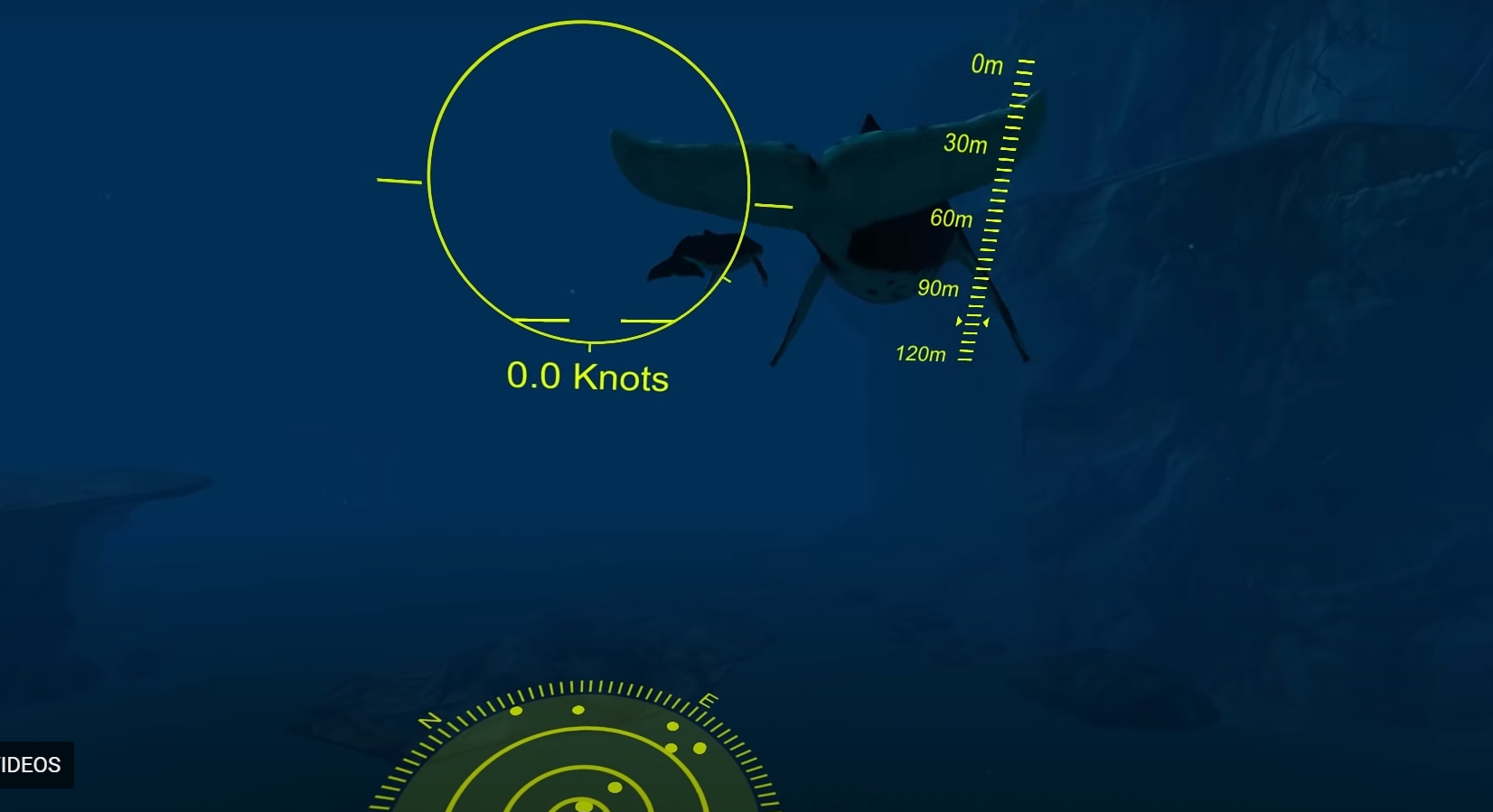


- "Eden One", in dem man durch Berge mit Ringen als Ziel, durch die man fliegen soll.


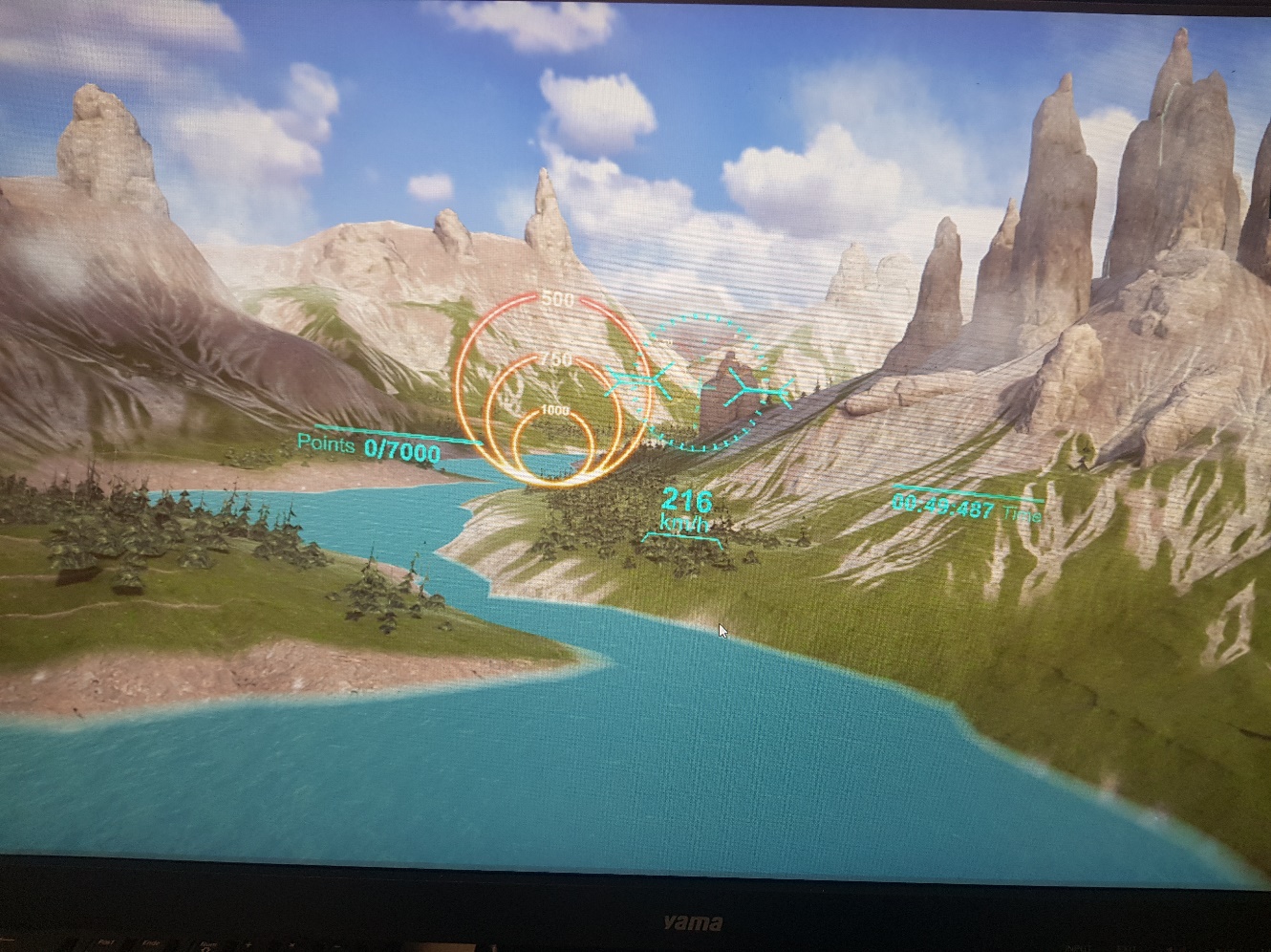


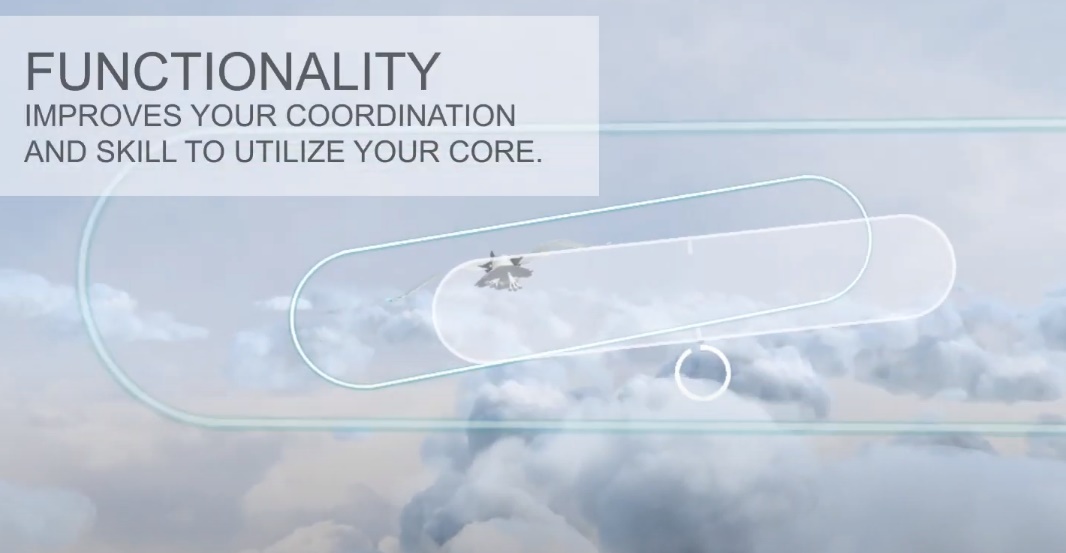
- "Core", bei dem man die Rumpfmuskulatur und die Koordination mit speziellen Übungen trainieren kann, die auf Stabilität (das Gerät in einer bestimmten Position halten), Funktionalität (das Gerät vorsichtig durch die Ringe bewegen) oder Reaktion (das Gerät schnell bewegen, um Bälle zu fangen) abzielen.

- Gravity", bei dem man aus einer fliegenden Kapsel abspringt mit Ringen als Ziel, durch die man fliegen soll um dann auf einem Flugzeugträger zu landen.


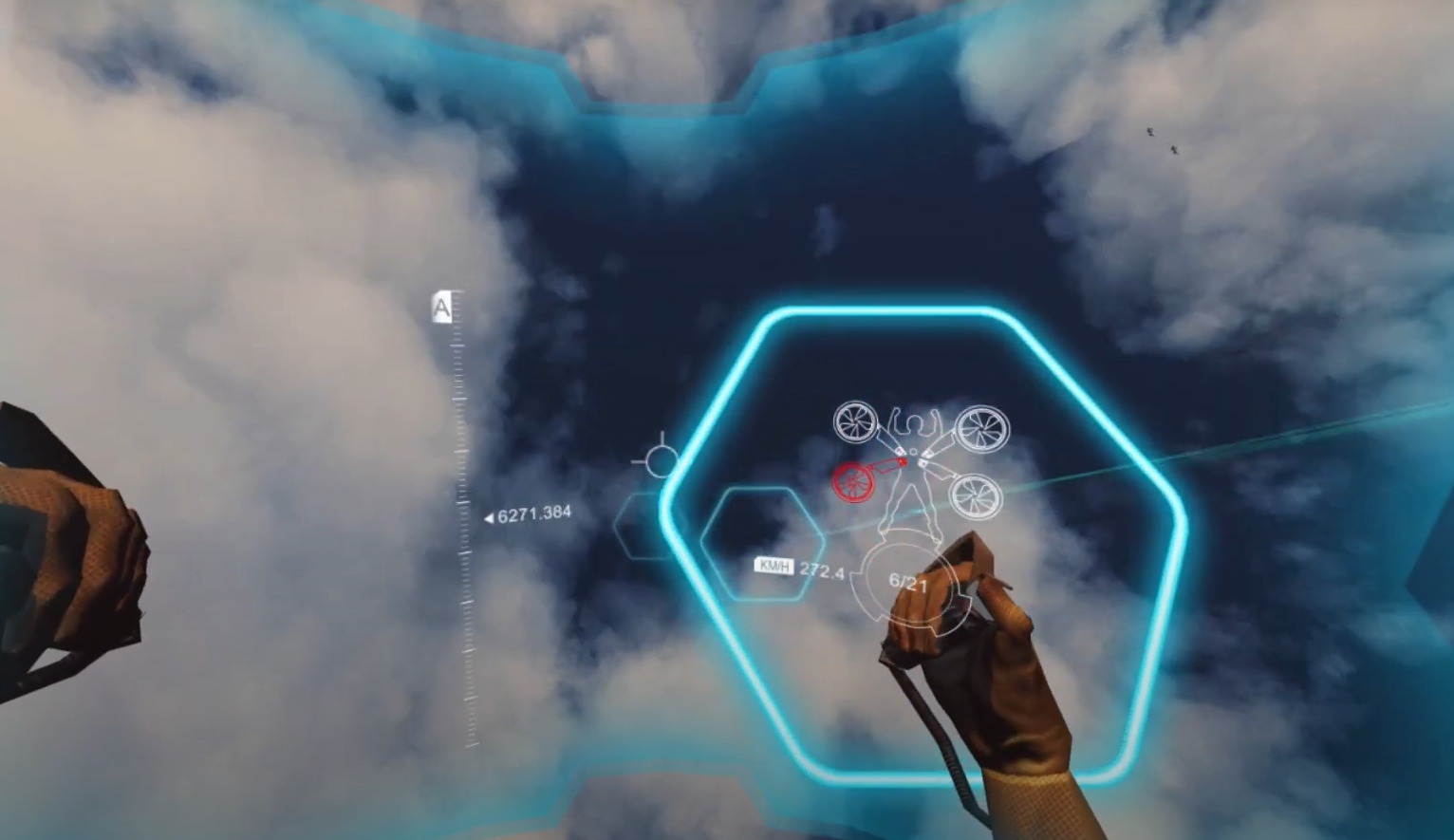


- "Flug", bei dem man Drohnen sucht und abschießt.


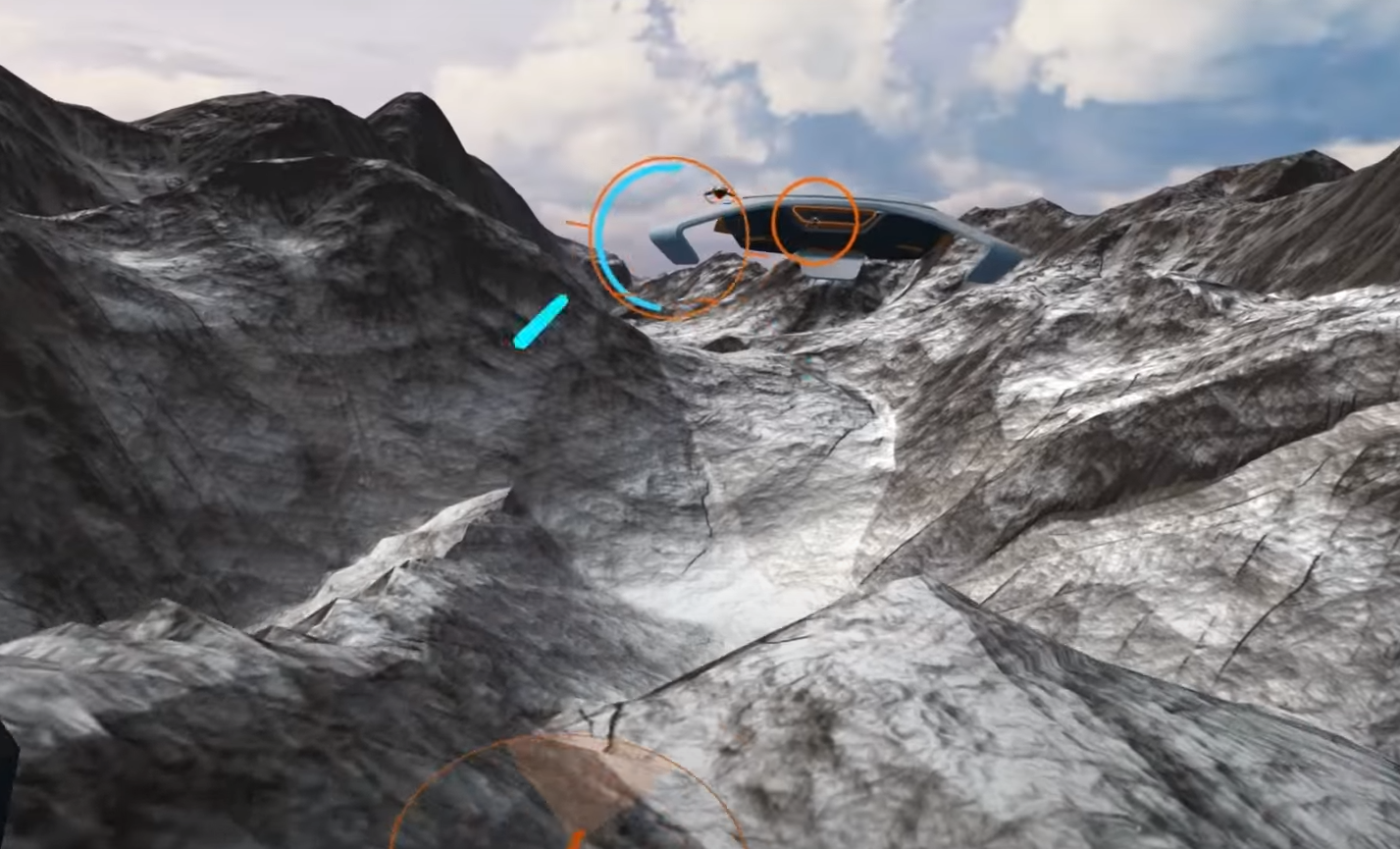


Während der Intervention können die Patienten frei wählen, welches Spiel sie spielen wollen, solange sie innerhalb ihrer festgelegten Anstrengungsphase bleiben. Die Spiele werden nacheinander eingeführt, wobei mit einfachen Aufgaben wie dem Fliegen ohne zusätzliche Herausforderung begonnen und der Schwierigkeitsgrad erhöht wird, sobald sich der Patient wohl fühlt. Falls die Patienten keine Vorliebe haben, wird empfohlen, die Sitzung mit einer einfachen Aufgabe zu beginnen, und die Schwierigkeit der weiteren Aufgaben wird danach bestimmt, wie anstrengend das erste Spiel empfunden wurde. Jedes Spiel dauert anfangs eine Minute, sobald sich die Patienten damit wohl fühlen, wird die Dauer in 30er-Intervallen erhöht. Die Patienten werden ermutigt, die Spiele zu wählen, bei denen sie sich am wohlsten fühlen.

**Aussuchen eines Spiels**

3) Aufwärmen

(entsprechend WHO recommendations)

- Im Stehen: Side bands, Ankle Taps (je 2-4mal)
- Im Sitzen: Shoulder shrugs, shoulder circles, Knee lifts, ankle circles (je 2-4mal)

**
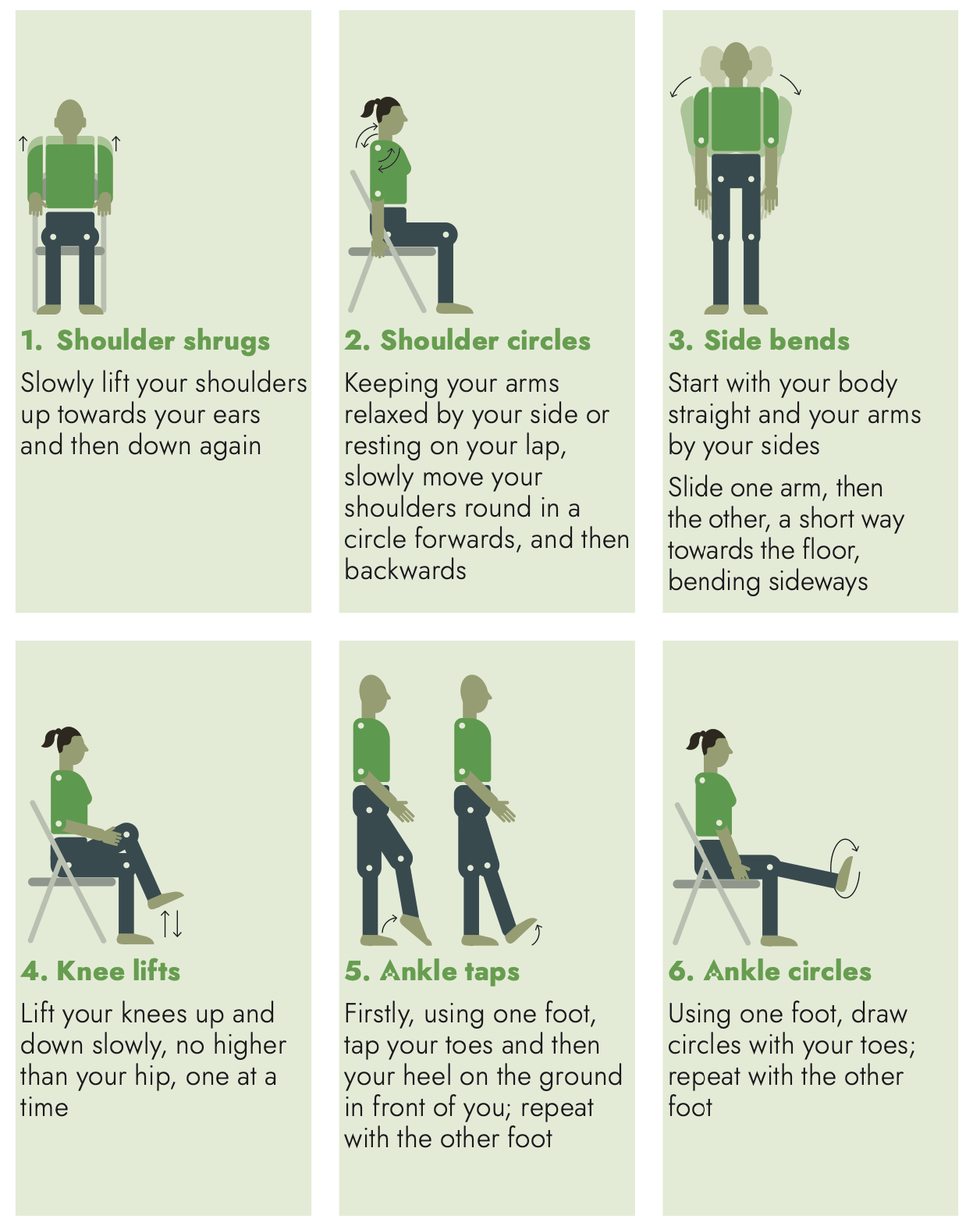
**

(Quelle WHO 2020 [Support for Rehabilitation: Self-Management after COVID-19 Related Illness (who.int)](https://www.who.int/publications/m/item/support-for-rehabilitation-self-management-after-covid-19-related-illness))

4) RPE-Ausgangswert

Das Level der Anstrengung, die im Alltag zu einer Verschlechterung der Symptome bis hin zur PEM führt wird erfragt. Das Anfangstraining richtet sich nach der vorherigen RPE-Phase

**(Phase 1: Preparation for return to exercise: RPE 0-1)**

- Patient*in gibt an, im Alltag schon bei geringer Anstrengung (RPE 2-3) zum Beispiel bei leichten Haushalts- oder Gartenarbeiten eine Verschlechterung der Symptome zu verspüren
- Übungen: Atemübungen, Dehnübungen)

**Phase 2: Low-intensity activity: RPE 2-3**

- Patient*in gibt an, im Alltag bei mittlerer Anstrengung (RPE 4-5) zum Beispiel beim Treppensteigen eine Verschlechterung der Symptome zu verspüren
- Übungen: Icaros flight bis RPE 2-3

**Phase 3: Moderate intensity activity: RPE 4-5**

- Patient*in gibt an, im Alltag bei mittlerer bis hoher Anstrengung (RPE 5-7) zum Beispiel Joggen, Schwimmen oder Fahrradfahren eine Verschlechterung der Symptome zu verspüren
- Übungen: Icaros flight bis RPE 4-5

**Phase 4: Moderate intensity exercises with coordination and functioning skills: RPE 5-7**

- Patient*in gibt an, im Alltag bei hoher Anstrengung (RPE 8-10) eine Verschlechterung der Symptome zu verspüren, obwohl die Übungen vor der Covid-Infektion gut toleriert wurden
- Übungen: Icaros flight bis RPE 5-7

**(Phase 5: Return to baseline exercise: RPE 8-10)**


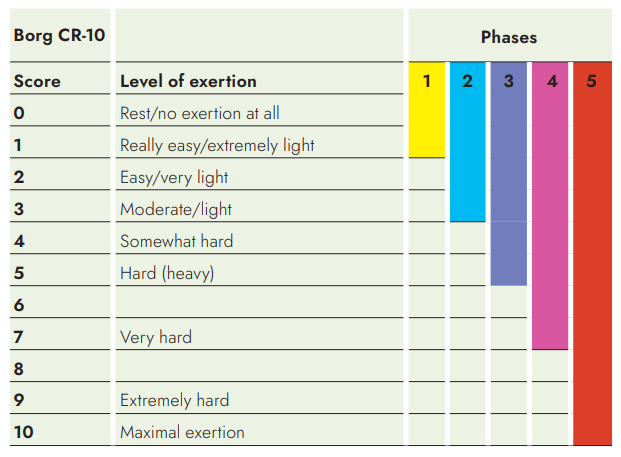


(Quelle WHO 2021 [Support for rehabilitation: self-management after COVID-19-related illness, second edition (who.int)](https://www.who.int/europe/publications/i/item/WHO-EURO-2021-855-40590-59892))

**Steuerung des Intensitätslevels der Trainingssession**

**->** Patient*in fliegt so viele Icaros-Flüge (a 50-90s), bis das RPE-Level unter der Obergrenze der RPE-Phase erreicht ist. Beispiel: RPE-Phase 4-5, Patienten fliegen bis sie ein RPE-Level von 4 angeben.

5) Cooling Down

- 2-minütiges Laufen auf der Stelle
- Im Stehen: Side bands, Ankle Taps (je 2-4mal)
- Im Sitzen: Shoulder shrugs, shoulder circles, Knee lifts, ankle circles (je 2-4mal)
- Im Stehen: Dehnen der Schulter, Waden und des Quadriceps (für 15-20s)
- Im Sitzen: Dehnen der Seiten und der ischiocruralen Muskulatur (für 15-20s)

6) Closure

- Positiven Zustand sicherstellen: Der Therapeut überprüft, ob der Proband in einem positiven Zustand die Sitzung verlässt. Sollte der Proband Unbehagen, Schmerzen oder Übelkeit angeben, Frägt der Therapeut, was der Proband tun kann, um sich gut zu fühlen (z.Bsp. Atemübung, frische Luft, Dehnung, Imaginations/ Entspannungsübung).
- Positiv-Rückmeldung: Ferner wird dem Patienten rückgemeldet, welche Fortschritte er gemacht hat

Core sessions

Ablaufplan

- 1. Ankommen: **5min**
- 2. Besprechung der Auswirkung der letzten Session: **5min**
- 3. Aufwärmen: **5min**
- 4. Training bis zum Erreichen des RPE-Levels unter der Obergrenze für die RPE-Phase der Patienten: **10 min**
- 5. Cooling-Down: **5min**
- 6. Klären von Fragen, Verabschieden: **5min**
- Puffer: **5min**
- Gesamt: **40min**

2) Auswirkungen des letzten Trainings

- Gab es nach dem letzten Training eine Symptomverschlechterung? Wenn ja, dann Rückstufung um eine RPE-Phase

- Gab es nach dem letzten Training eine Verbesserung oder ein Gleichbleiben der Symptome? Fortschreiten in die nächsthöhere RPE-Phase bei Ausbleiben einer Verschlechterung über sieben Tage, also über die letzten beiden Trainingssessions

3) Aufwärmen

- Im Stehen: Side bands, Ankle Taps (je 2-4mal)
- Im Sitzen: Shoulder shrugs, shoulder circles, Knee lifts, ankle circles (je 2-4mal)

4) Training

5) Cooling Down

- 2-minütiges Laufen auf der Stelle
- Im Stehen: Side bands, Ankle Taps (je 2-4mal)
- Im Sitzen: Shoulder shrugs, shoulder circles, Knee lifts, ankle circles (je 2-4mal)
- Im Stehen: Dehnen der Schulter, Waden und des Quadriceps (für 15-20s)
- Im Sitzen: Dehnen der Seiten und der ischiocruralen Muskulatur (für 15-20s)

6) Closure

- Positiven Zustand sicherstellen: Der Therapeut überprüft, ob der Proband in einem positiven Zustand die Sitzung verlässt. Sollte der Proband Unbehagen, Schmerzen oder Übelkeit angeben, Frägt der Therapeut, was der Proband tun kann, um sich gut zu fühlen (z.Bsp. Atemübung, frische Luft, Dehnung, Imaginations/Entspannungsübung).
- Positiv-Rückmeldung: Ferner wird dem Patienten rückgemeldet, welche Fortschritte er gemacht hat

Closing session

Ablaufplan

- 1. Ankommen: **5min**
- 2. Besprechung der Auswirkung der letzten Session, Vergleich mit der ersten Session zur Betrachtung des Fortschritts: **5min**
- 3. Aufwärmen: **5min**
- 4. Training bis zum Erreichen des RPE-Levels unter der Obergrenze für die RPE-Phase der Patienten: **10 min**
- 5. Cooling-Down: **5min**
- 6. Klären von Fragen, Verabschieden, Feedback: **10min**
- Puffer: **5min**
- Gesamt: **40min**

2) Auswirkungen des letzten Trainings

- Gab es nach dem letzten Training eine Symptomverschlechterung? Wenn ja, dann Rückstufung um eine RPE-Phase

- Gab es nach dem letzten Training eine Verbesserung oder ein Gleichbleiben der Symptome? Fortschreiten in die nächsthöhere RPE-Phase bei Ausbleiben einer Verschlechterung über sieben Tage, also über die letzten beiden Trainingssessions

3) Aufwärmen

- Im Stehen: Side bands, Ankle Taps (je 2-4mal)
- Im Sitzen: Shoulder shrugs, shoulder circles, Knee lifts, ankle circles (je 2-4mal)

4) Training

5) Cooling Down

- 2-minütiges Laufen auf der Stelle
- Im Stehen: Side bands, Ankle Taps (je 2-4mal)
- Im Sitzen: Shoulder shrugs, shoulder circles, Knee lifts, ankle circles (je 2-4mal)
- Im Stehen: Dehnen der Schulter, Waden und des Quadriceps (für 15-20s)
- Im Sitzen: Dehnen der Seiten und der ischiocruralen Muskulatur (für 15-20s)

6) Closure

- Positiven Zustand sicherstellen: Der Therapeut überprüft, ob der Proband in einem positiven Zustand die Sitzung verlässt. Sollte der Proband Unbehagen, Schmerzen oder Übelkeit angeben, Frägt der Therapeut, was der Proband tun kann, um sich gut zu fühlen (z.Bsp. Atemübung, frische Luft, Dehnung, Imaginations/Entspannungsübung).
- Positiv-Rückmeldung: Ferner wird dem Patienten rückgemeldet, welche Fortschritte er gemacht hat
- Klären von offenen Fragen
- Verabschieden
- Feedback
