# Supplementary material for: Efficacy of supervised immersive virtual reality-based training for the treatment of chronic fatigue in post-COVID syndrome: study protocol for a double-blind randomized controlled trial (IFATICO Trial)
Source: Trials. 2024 Apr 3;25:232. doi: 10.1186/s13063-024-08032-w (PMC10993519; doi:10.1186/s13063-024-08032-w)
Supplement: Supplementary file 5 — Additional file 5. SOP: Training manual control group. [file 13063_2024_8032_MOESM5_ESM.docx]

**Manual IFATICO Vergleichsgruppe**

Inhalt

[Opening session 2](#_heading=h.gjdgxs)

[Ablaufplan 2](#_heading=h.30j0zll)

[2) Aufklärung, Erklärung der Übungen 2](#_heading=h.1fob9te)

[3) Aufwärmen 3](#_heading=h.3znysh7)

[4) RPE-Ausgangswert 4](#_heading=h.2et92p0)

[5) Cooling Down 5](#_heading=h.tyjcwt)

[6) Closure 6](#_heading=h.3dy6vkm)

[Core sessions 7](#_heading=h.1t3h5sf)

[Ablaufplan 7](#_heading=h.4d34og8)

[2) Auswirkungen des letzten Trainings 7](#_heading=h.2s8eyo1)

[3) Aufwärmen 7](#_heading=h.17dp8vu)

[4) Training 8](#_heading=h.3rdcrjn)

[5) Cooling Down 9](#_heading=h.26in1rg)

[6) Closure 10](#_heading=h.lnxbz9)

[Closing session 11](#_heading=h.35nkun2)

[Ablaufplan 11](#_heading=h.1ksv4uv)

[2) Auswirkungen des letzten Trainings 11](#_heading=h.44sinio)

[3) Aufwärmen 11](#_heading=h.2jxsxqh)

[4) Training 11](#_heading=h.z337ya)

[5) Cooling Down 11](#_heading=h.3j2qqm3)

[6) Closure 12](#_heading=h.1y810tw)

# Opening session

## Ablaufplan

- 1. Ankommen: **5min**
- 2. Aufklärung, Erklären der Übungen, Zuordnen zu einer RPE-Phase: **10 min**
- 3. Aufwärmen: **5min**
- 4. Ermitteln eines Ausgangswert für die Anzahl an Wiederholungen: Durchführung der Übungen mit der Anzahl an Wiederholungen bis das RPE-Level unter der RPE-Obergrenze der jeweiligen Phase erreicht ist (+Dokumentation): **15min**
- 5. Cooling-Down: **5min**
- 6. Klären von Fragen, Verabschieden: **5min**
- Puffer: **5min**
- Gesamt: **50min**

## 2) Aufklärung, Erklärung der Übungen

**Aufklärung**

- Beziehung von neuromuskulärer Kontrolle und Fatigue

**Erklärung der Übungen**

- Patienten können jederzeit pausieren oder unterbrechen, wenn es Ihnen nicht gut geht
- Es gibt zwei Übungen für die Arme und zwei Übungen für die Beine, davon machen sie je bis zu 10 Wiederholungen (1 Set)
- Nach jedem Set wird eine 1-minütige Pause gemacht, insgesamt werden drei Sets gemacht
- Werden 10 Wiederholung gut toleriert, so kann das Gewicht/ der Schwierigkeitsgrad erhöht werden

## 3) Aufwärmen

- Im Stehen: Side bands, Ankle Taps (je 2-4mal)
- Im Sitzen: Shoulder shrugs, shoulder circles, Knee lifts, ankle circles (je 2-4mal)

**
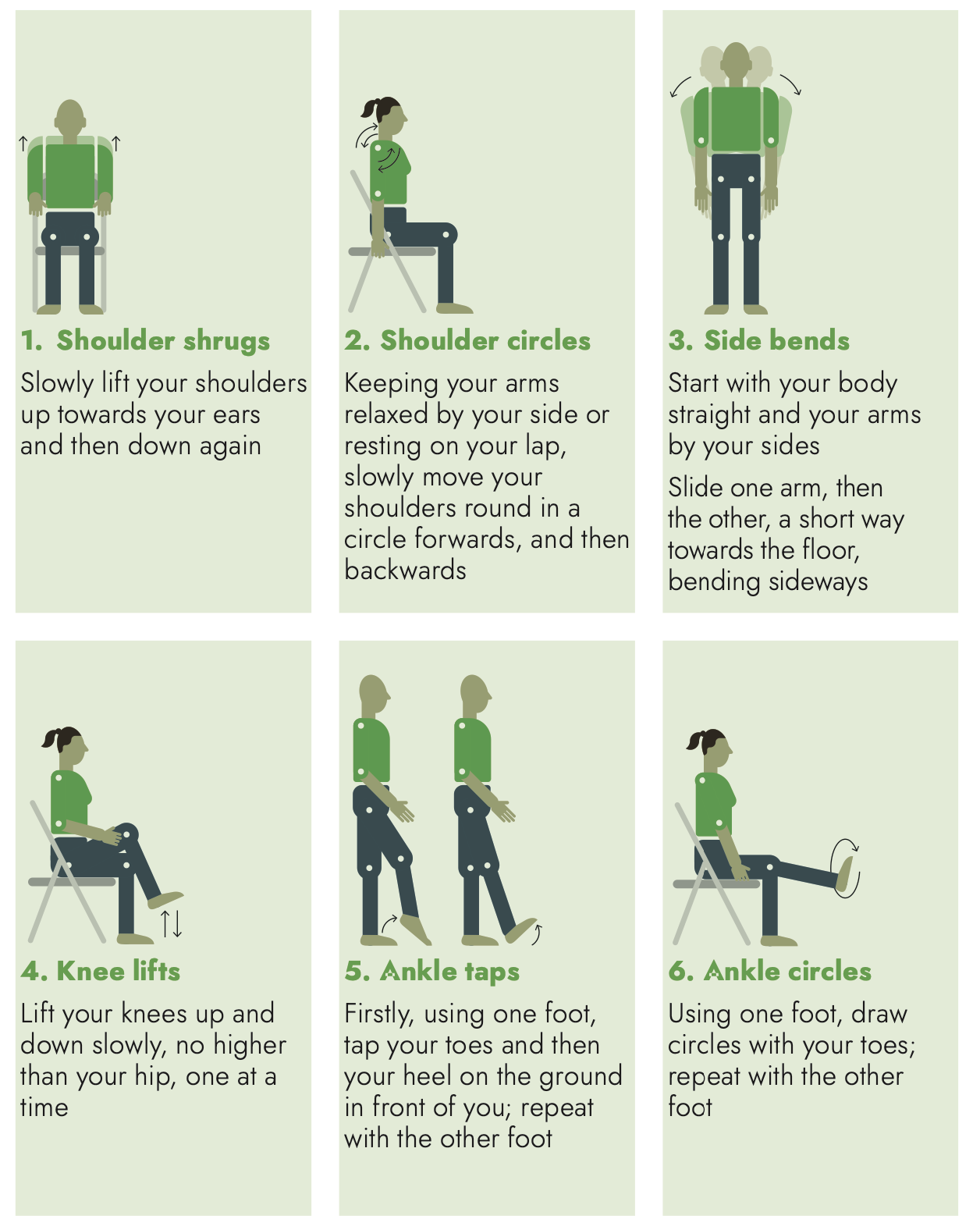
**

(Quelle WHO 2020 [Support for Rehabilitation: Self-Management after COVID-19 Related Illness (who.int)](https://www.who.int/publications/m/item/support-for-rehabilitation-self-management-after-covid-19-related-illness))

## 4) RPE-Ausgangswert

Das Level der Anstrengung, die im Alltag zu einer Verschlechterung der Symptome bis hin zur PEM führt wird erfragt. Das Anfangstraining richtet sich nach der vorherigen RPE-Phase

**(Phase 1: Preparation for return to exercise: RPE 0-1)**

- Patient*in gibt an, im Alltag schon bei geringer Anstrengung (RPE 2-3) zum Beispiel bei leichten Haushalts- oder Gartenarbeiten eine Verschlechterung der Symptome zu verspüren
- Übungen: Atemübungen, Dehnübungen)

**Phase 2: Low-intensity activity: RPE 2-3**

- Patient*in gibt an, im Alltag bei mittlerer Anstrengung (RPE 4-5) zum Beispiel beim Treppensteigen eine Verschlechterung der Symptome zu verspüren
- Übungen: Kraftübungen bis RPE 2-3

**Phase 3: Moderate intensity activity: RPE 4-5**

- Patient*in gibt an, im Alltag bei mittlerer bis hoher Anstrengung (RPE 5-7) zum Beispiel Joggen, Schwimmen oder Fahrradfahren eine Verschlechterung der Symptome zu verspüren
- Übungen: Kraftübungen bis RPE 4-5

**Phase 4: Moderate intensity exercises with coordination and functioning skills: RPE 5-7**

- Patient*in gibt an, im Alltag bei hoher Anstrengung (RPE 8-10) eine Verschlechterung der Symptome zu verspüren, obwohl die Übungen vor der Covid-Infektion gut toleriert wurden
- Übungen: Kraftübungen bis RPE 5-7

**(Phase 5: Return to baseline exercise: RPE 8-10)**


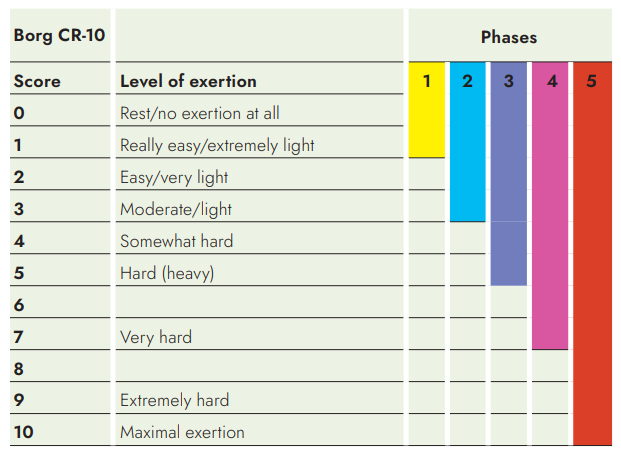


(Quelle WHO 2021 [Support for rehabilitation: self-management after COVID-19-related illness, second edition (who.int)](https://www.who.int/europe/publications/i/item/WHO-EURO-2021-855-40590-59892))

**->** Patient*in macht so viele Wiederholungen einer Übung pro Set, bis das RPE-Level unter der Obergrenze der RPE-Phase erreicht ist. Beispiel: RPE-Phase 4-5-Patienten fliegen bis sie ein RPE-Level von 4 angeben

- Zur Ermittlung des Anfangswertes machen Patienten von einer Übung am Stück so viele Wiederholungen, dass das RPE-Level unter der Obergrenze der jeweiligen Phase erreicht wird. Dieser Wert wird für die folgenden Sessions dreigeteilt und abgerundet, um die anfängliche Zahl der Wiederholungen pro Set zu ermitteln

## 5) Cooling Down

- 2-minütiges Laufen auf der Stelle
- Im Stehen: Side bands, Ankle Taps (je 2-4mal)
- Im Sitzen: Shoulder shrugs, shoulder circles, Knee lifts, ankle circles (je 2-4mal)
- Im Stehen: Dehen der Schulter, Waden und des Quadriceps (für 15-20s)
- Im Sitzen: Dehnen der Seiten und der ischiocruralen Muskulatur (für 15 bis 20s)

## 6) Closure

- Positiven Zustand sicherstellen: Der Therapeut überprüft, ob der Proband in einem positiven Zustand die Sitzung verlässt. Sollte der Proband Unbehagen, Schmerzen oder Übelkeit angeben, Frägt der Therapeut, was der Proband tun kann, um sich gut zu fühlen (z.Bsp. Atemübung, frische Luft, Dehnung, Imaginations/Entspannungsübung).
- Positiv-Rückmeldung: Ferner wird dem Patienten rückgemeldet, welche Fortschritte er gemacht hat

# Core sessions

## Ablaufplan

- 1. Ankommen: **5min**
- 2. Besprechung der Auswirkung der letzten Session: **5min**
- 3. Aufwärmen: **5min**
- 4. Training bis zum Erreichen des RPE-Levels unter der Obergrenze für die RPE-Phase der Patienten: **20 min**
- 5. Cooling-Down: **5min**
- 6. Klären von Fragen, Verabschieden: **5min**
- Puffer: **5min**
- Gesamt: **50min**

## 2) Auswirkungen des letzten Trainings

- Gab es nach dem letzten Training eine Symptomverschlechterung? Wenn ja, dann Rückstufung um eine RPE-Phase

- Gab es nach dem letzten Training eine Verbesserung oder ein Gleichbleiben der Symptome? Fortschreiten in die nächsthöhere RPE-Phase bei Ausbleiben einer Verschlechterung über sieben Tage, also über die letzten beiden Trainingssessions

## 3) Aufwärmen

- Im Stehen: Side bands, Ankle Taps (je 2-4mal)
- Im Sitzen: Shoulder shrugs, shoulder circles, Knee lifts, ankle circles (je 2-4mal)

## 4) Training


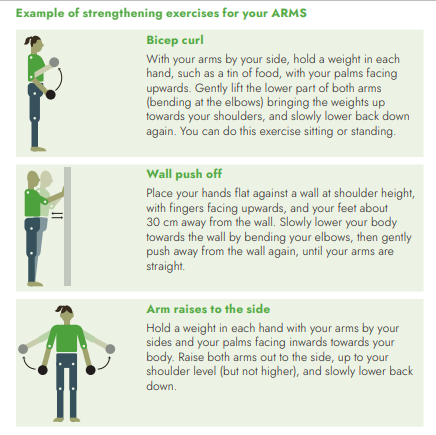


(Quelle WHO 2021 [Support for rehabilitation: self-management after COVID-19-related illness, second edition (who.int)](https://www.who.int/europe/publications/i/item/WHO-EURO-2021-855-40590-59892))


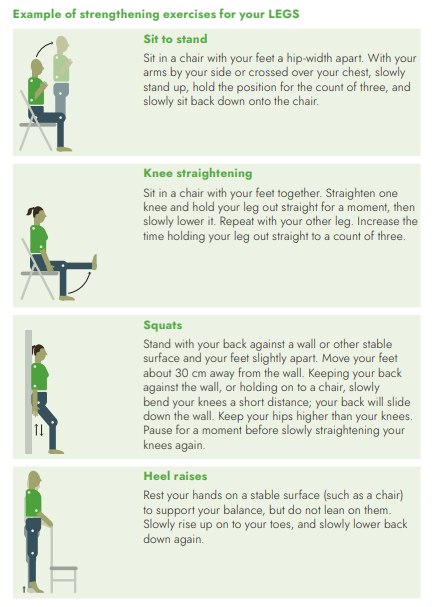


(Quelle WHO 2021 [Support for rehabilitation: self-management after COVID-19-related illness, second edition (who.int)](https://www.who.int/europe/publications/i/item/WHO-EURO-2021-855-40590-59892))

Die WHO-Empfehlungen umfassen drei Übungen für den Oberkörper (Bizepscurls, Liegestütze an der Wand und seitliches Anheben) und vier Übungen für den Unterkörper (Sit to Stand, Kniestrecker, Kniebeugen und Fersenerhöhung). Den Patienten werden alle Übungen gezeigt und erklärt, und sie können dann zwei Übungen für den Oberkörper und zwei für den Unterkörper auswählen, die für sie am besten geeignet sind. Eine Trainingseinheit lang werden diese Übungen beibehalten, aber in der nächsten Sitzung können die Patienten die Übungen wechseln. Die Patienten werden ermutigt, bewusst zu atmen, und sie werden bei der korrekten Ausführung der Übungen angeleitet.

## 5) Cooling Down

- 2-minütiges Laufen auf der Stelle
- Im Stehen: Side bands, Ankle Taps (je 2-4mal)
- Im Sitzen: Shoulder shrugs, shoulder circles, Knee lifts, ankle circles (je 2-4mal)
- Im Stehen: Dehen der Schulter, Waden und des Quadriceps (für 15-20s)
- Im Sitzen: Dehnen der Seiten und der ischiocruralen Muskulatur (für 15-20s)

## 6) Closure

- Positiven Zustand sicherstellen: Der Therapeut überprüft, ob der Proband in einem positiven Zustand die Sitzung verlässt. Sollte der Proband Unbehagen, Schmerzen oder Übelkeit angeben, Frägt der Therapeut, was der Proband tun kann, um sich gut zu fühlen (z.Bsp. Atemübung, frische Luft, Dehnung, Imaginations/Entspannungsübung).
- Positiv-Rückmeldung: Ferner wird dem Patienten rückgemeldet, welche Fortschritte er gemacht hat

# Closing session

## Ablaufplan

- 1. Ankommen: **5min**
- 2. Besprechung der Auswirkung der letzten Session, Vergleich mit der ersten Session zur Betrachtung des Fortschritts: **5min**
- 3. Aufwärmen: **5min**
- 4. Training bis zum Erreichen des RPE-Levels unter der Obergrenze für die RPE-Phase der Patienten: **20 min**
- 5. Cooling-Down: **5min**
- 6. Klären von Fragen, Verabschieden, Feedback: **10min**
- Puffer: **5min**
- Gesamt: **50min**

## 2) Auswirkungen des letzten Trainings

- Gab es nach dem letzten Training eine Symptomverschlechterung? Wenn ja, dann Rückstufung um eine RPE-Phase

- Gab es nach dem letzten Training eine Verbesserung oder ein Gleichbleiben der Symptome? Fortschreiten in die nächsthöhere RPE-Phase bei Ausbleiben einer Verschlechterung über sieben Tage, also über die letzten beiden Trainingssessions

## 3) Aufwärmen

- Im Stehen: Side bands, Ankle Taps (je 2-4mal)
- Im Sitzen: Shoulder shrugs, shoulder circles, Knee lifts, ankle circles (je 2-4mal)

## 4) Training

## 5) Cooling Down

- 2-minütiges Laufen auf der Stelle
- Im Stehen: Side bands, Ankle Taps (je 2-4mal)
- Im Sitzen: Shoulder shrugs, shoulder circles, Knee lifts, ankle circles (je 2-4mal)
- Im Stehen: Dehen der Schulter, Waden und des Quadriceps (für 15-20s)
- Im Sitzen: Dehnen der Seiten und der ischiocruralen Muskulatur (für 15-20s)

## 6) Closure

- Positiven Zustand sicherstellen: Der Therapeut überprüft, ob der Proband in einem positiven Zustand die Sitzung verlässt. Sollte der Proband Unbehagen, Schmerzen oder Übelkeit angeben, Frägt der Therapeut, was der Proband tun kann, um sich gut zu fühlen (z.Bsp. Atemübung, frische Luft, Dehnung, Imaginations/Entspannungsübung).
- Positiv-Rückmeldung: Ferner wird dem Patienten rückgemeldet, welche Fortschritte er gemacht hat
- Klären offener Fragen
- Feedback und Rückblick auf die Entwicklung über die Therapie
