# Supplementary material for: Efficacy of supervised immersive virtual reality-based training for the treatment of chronic fatigue in post-COVID syndrome: study protocol for a double-blind randomized controlled trial (IFATICO Trial)
Source: Trials. 2024 Apr 3;25:232. doi: 10.1186/s13063-024-08032-w (PMC10993519; doi:10.1186/s13063-024-08032-w)
Supplement: Supplementary file 6 — Additional file 6. Patient information before randomisation. [file 13063_2024_8032_MOESM6_ESM.pdf]

UniversitätsKlinikum Heidelberg

Klinik für Psychosomatische Medizin | Im Neuenheimer Feld 410 | 69120 Heidelberg

Überprüfung der Akzeptanz, Alltagstauglichkeit und Nutzbarkeit eines personalisierten Trainingsprogramms zur Reduktion von Post-Covid-assoziierten Beschwerden (IFATICO)

Medizinische Klinik (Krehl-Klinik)

Abteilung Innere Medizin II
Klinik für Allgemeine Innere Medizin und Psychosomatik
(Standort Neuenheim)

Prof.(apl.) Dr. med. Jonas Tesarz
Klinik für Allgemeine Innere Medizin und Psychosomatik des Universitätsklinikums Heidelberg
Sektion Integrierte Psychosomatik
(AG Schmerz)

Im Neuenheimer Feld 410, 69120 Heidelberg
Tel. : +49 6221-5637862;
Fax: +49 6221-568450
E-Mail: jonas.tesarz@med.uni-heidelberg.de

Sehr geehrte Patientin, sehr geehrter Patient,
mit diesem Schreiben möchten wir Sie dazu einladen, an der oben genannten Studie zur Überprüfung der Akzeptanz, Alltagstauglichkeit und Nutzbarkeit eines personalisierten Trainingsprogramms zur Reduktion von Post-Covid-assoziierten Beschwerden teilzunehmen. Bitte lesen Sie sich die folgenden Informationen sorgfältig durch. Lassen Sie sich ausreichend Zeit und stellen Sie den Studienmitarbeitern alle Fragen, die für Sie wichtig sind. Sie können dann entscheiden, ob Sie teilnehmen möchten oder nicht.

An wen richtet sich die Studie?

Im Zuge der SARS-CoV-2-Pandemie haben sich weltweit hunderte von Millionen von Menschen infiziert. Bei einigen der Infizierten treten Langzeitbeschwerden auf, die über Wochen bis Monate andauern können und häufig als "Long-Covid" oder "Post-Covid-Syndrom" bezeichnet werden. Nach derzeitigem Kenntnisstand sind die häufigsten Spätsymptome Müdigkeit, Konzentrationsschwäche und Kurzatmigkeit, sowie eine ausgeprägte Belastungsintoleranz – Zustandsverschlechterung nach Belastung, manchmal auch „Post-Exertional Malaise“ genannt. Weitere Symptome können Übelkeit, Kopfschmerz Appetitlosigkeit, Durchfall, Verlust des Geruchs- und Geschmackssinns, Schwindel, Schmerzen oder psychische Symptome sein. Betroffene, die vorher sowohl körperlich als auch psychisch keinerlei Beschwerden hatten, sehen sich plötzlich mit deutlichen Leistungseinbußen sowohl in ihren geistigen als auch körperlichen Fähigkeiten konfrontiert. Wenn Sie sich von dieser Beschreibung angesprochen fühlen und Ihre akute Corona-Infektion 12 Wochen oder länger zurückliegt, möchten wir Sie herzlich zu einer Teilnahme an unserer Studie einladen

Welches Ziel verfolgt die Studie?

Obwohl Post-Covid Beschwerden oft massiv sind und in klinischen Untersuchungen nachgewiesen werden können, ist deren Entstehung noch weitestgehend unverstanden. Deswegen richten sich bisherige Therapieansätze lediglich gegen die Symptome, nicht aber die Ursache der Krankheit.

Es wird dabei empfohlen, einerseits körperlich und mental aktiv zu bleiben, um den Körper und das Nervensystem zu „trainieren“, also eine Verbesserung zu stimulieren. Andererseits muss auch eine Überanstrengung vermieden werden, da sie bei Patient*innen mit Belastungsintoleranz zu einer Verschlechterung der Symptome führen kann. (Deswegen wird oft ein sogenanntes „Pacing“ empfohlen: Patient*innen sollen dabei auf ihren eigenen Körper hören, sich ihre Energie gut einteilen und dabei innerhalb ihrer eigenen Leistungsgrenzen bleiben.) Bisherige Rehabilitationsprogramme versuchen, diese Besonderheiten zu berücksichtigen, stoßen aber oft an ihre Grenzen.

Neue neuromuskuläre Trainingstechniken, welche sowohl den Körper wie auch das Gehirn mit minimaler physischer und psychischer Belastung stimulieren, sind ein vielversprechender Lösungsansatz. Das Gehirn kann so Schritt für Schritt seine Leistungsfähigkeit wieder erlernen ohne die individuellen Grenzen übertreten zu müssen.

Das übergeordnete Ziel dieser Studie ist es daher, den Einfluss eines personalisierten neuromuskulären Trainingsprogramms auf Post-Covid-Beschwerden, Einschränkungen und Lebensqualität der Patient*innen zu untersuchen. Dazu möchten wir, begleitend zur Trainingstherapie wiederholt Daten von Ihnen erheben und auswerten.

Wie läuft die Studie ab?

Vor Ihrer Teilnahme wird ein **Telefongespräch oder persönliches Gespräch** durchgeführt, in welchem Sie uns zum einen weitere Fragen zu unserer Studie stellen können, und wir zum anderen überprüfen, ob Sie für unsere Studie geeignet sind.

Sie bekommen dann zunächst einen Fragebogen von uns, den Sie bequem von zu Hause ausfüllen können. Dabei können Sie wählen, ob Sie diesen in Papierform (mit Rückumschlag) oder online ausfüllen möchten. Die Fragebögen enthalten ca. 200 Fragen zum Ankreuzen, das Ausfüllen dauert ca. 30 min. Anhand der Fragebögen werden gezielt Symptome (z.B. zu möglichen Post-Covid-assoziierten Beschwerden, zu aktuellen Belastungsfaktoren, sowie zur allgemeinen Lebensqualität) und Erkrankungen erfragt und Sie werden gebeten, Fragen zu ihren allgemeinen Lebensumständen (z.B. soziale Teilhabe) zu beantworten.

Im Rahmen des ersten Studientermins werden wir zunächst einige kurze klinische Funktionstests mit Ihnen durchführen. Dies beinhaltet einen 6-Minuten-Gehtest (ein Test bei welchem wir Sie bitten werden, über einen Zeitraum von 6 Minuten der eigenen Leistung entsprechend möglichst weit zu gehen), eine Messung der Handkraft, sowie einen kurzen Test ihrer Konzentrations- und Merkfähigkeit. Ergänzend werden wir dann eine sogenannte quantitative sensorische Testung durchführen. Dabei wird eine Luftdruckmanschette an Ihren Unterschenkel angebracht und aufgepumpt, bis Sie angeben, Schmerzen zu empfinden. Dann wird die Luft wieder abgelassen. Wichtig hier ist zu beachten, dass Sie diese Testung jederzeit komplett stoppen können und es zu keinen nachhaltigen Schmerzen oder etwa Gewebsschäden kommt. Für diesen ersten Termin sollten Sie insgesamt eine Stunde einplanen.

Anschließend werden Sie einem von zwei möglichen personalisierten neuromuskulären Trainingsprogrammen zur Reduktion von Post-Covid-assoziierten Beschwerden **zufällig zugeordnet und über die jeweilige Zuteilung informiert**. Die Wahrscheinlichkeit, in

einem der beiden Programme zu landen, beträgt dabei jeweils 50%. Diese Therapieprogramme orientieren sich an den allgemeinen Empfehlungen der Weltgesundheitsorganisation (WHO) zum Trainingsmanagement bei Post-Covid-assoziierter Müdigkeit und umfassen zwei Sitzungen pro Woche von jeweils einer Dauer von 20-30 Minuten über insgesamt 6 Wochen. Das Trainingsprogramm orientiert sich dabei an den allgemeinen WHO-Empfehlungen zum Trainingsmanagement bei Post-Covid-assoziierter Müdigkeit. Nach Beendigung des Trainingsprogramms bekommen Sie einen Fragebogen, je nach Wunsch per Post oder online, zugeschickt und erhalten noch einen abschließenden Studientermin, bei welchem wir erneut die bereits anfangs durchgeführten kurzen klinischen Funktionstests mit Ihnen durchführen. Wir erfragen hier ganz konkret ihre Erfahrungen mit der Behandlung und erneut ihr allgemeines Befinden und ihre Symptome. Bitte beachten Sie, dass aus statistischen Gründen auch dann ein Assessment durchgeführt und der Fragebogen beantwortet werden soll, wenn Sie sich dazu entscheiden, die Studie abzubrechen. Nach 3 und 12 Monaten erhalten sie erneut einen Fragebogen, dieser wird ihnen auf dem von ihnen gewünschten Weg zugeschickt.

Was für eine Behandlung erhalte ich?
Das Trainingsprogramm orientiert sich an den allgemeinen WHO-Empfehlungen zum Trainingsmanagement bei Post-Covid-assoziierter Müdigkeit. Es umfasst insgesamt 12-18 Sitzungen a 30 Minuten und erstreckt sich über einen Zeitraum von insgesamt 6 Wochen (2-3 Sitzungen pro Woche). Das bedeutet, dass Sie wiederholt gebeten werden, Ihr Erschöpfungslevel anzugeben und wir die Anzahl der Wiederholungen einer Übung daran anpassen, um Überanstrengung zu vermeiden. Die Übungen sollen die Muskulatur von Armen, Beinen, Rücken und Bauch sowie Koordination und Reaktionsfähigkeit trainieren. Die einzelnen Trainingseinheiten finden unter persönlicher Anleitung durch einen in dem Trainingsprogram geschulten Trainer statt. Für die einzelnen Trainingseinheiten wird eine bequeme und elastische Kleidung empfohlen.

Habe ich einen persönlichen Nutzen?
Wenn sie an der oben genannten Therapie teilnehmen, erhalten Sie eine im Vergleich zur ambulanten Regelversorgung intensivierte und personalisierte Behandlung. Es ist daher davon auszugehen, dass sie einen persönlichen Nutzen haben werden. 1Fallen bei den klinischen Tests oder beim Ausfüllen der Fragebögen Symptome oder Problemstellungen auf, so können wir mit Ihnen zusammen weitere Therapiemöglichkeiten besprechen und Ihnen ggf. frühere Hilfe zukommen lassen.
Sie leisten durch ihre Teilnahme auf jeden Fall für andere Patienten einen wichtigen Beitrag zum weiteren Ausbau und zur Verbesserung von intensiven ambulanten Therapieangeboten.

Welche Risiken sind mit der Teilnahme verbunden?
Bei Patient*innen mit chronischer Erschöpfung/ Müdigkeit, kann körperliches Training zu einer Überlastung und Verschlechterung der Symptome führen. Bei der Gestaltung des Trainingsprogramms haben wir aber größten Wert daraufgelegt, dieses Risiko zu

minimieren. Wir werden während den einzelnen Terminen aufpassen, dass Ihre individuellen Limits nicht überschritten werden und Sie können eine Sitzung selbstverständlich jederzeit abbrechen oder pausieren, sollte es Ihnen nicht gut gehen.
Weiterhin werden wir die Luftdruckmanschetten während der quantitativen sensorischen Testung aufpumpen, bis Sie angeben, Schmerzen zu haben. Danach wird die Luft aber wieder abgelassen, sodass auch der Schmerz ohne dauerhafte Schäden wieder nachlässt. In manchen Fällen kann es durch die Beantwortung der Fragebögen zur Bewusstwerdung von Problemstellungen kommen und damit einhergehend zu verschlechterter Stimmung. Auch wenn wir davon ausgehen, dass dies vorübergehend ist, dürfen Sie uns in solchen Fällen gerne kontaktieren um weitere Unterstützungsmöglichkeiten zu besprechen.

Informationen zum Datenschutz

Allgemein: Die ärztliche Schweigepflicht und datenschutzrechtliche Bestimmungen werden eingehalten. Während der Studie werden medizinische Befunde und/oder persönliche Informationen von Ihnen erhoben und in der Prüfstelle in Ihrer persönlichen Akte niedergeschrieben oder elektronisch gespeichert. Wenn Sie uns in der Einwilligungserklärung explizit die Erlaubnis dazu geben und die jeweiligen Kollegen zu Zwecken der Studie von der Schweigepflicht befreien, erheben wir auch Daten Ihrer Hausärzte/ anderer Fachärzte. Die für die Studie wichtigen Daten werden zusätzlich in pseudonymisierter[1] Form gespeichert, ausgewertet und gegebenenfalls an Forschungseinrichtungen *(z.Bsp. Universitäten/Kliniken/Forschungsinstitute etc.)* weitergegeben, u.U. auch in Länder, in denen die Datenschutzanforderungen niedriger sind als in der Europäischen Union. Es handelt sich um Länder, für die die Europäische Kommission ein angemessenes gesetzliches Datenschutzniveau festgestellt hat. Die Studienleitung wird alle angemessenen Schritte unternehmen, um den Schutz Ihrer Daten gemäß den Datenschutzstandards der Europäischen Union zu gewährleisten. Die Daten sind gegen unbefugten Zugriff gesichert. Eine Entschlüsselung erfolgt nur bei Rücktritt von der Studie zum Zweck der Datenvernichtung. Sobald es nach dem Forschungs- oder Statistikzweck möglich ist, werden die personenbezogenen Daten

[1] „Pseudonymisierung" ist die Verarbeitung personenbezogener Daten in einer Weise, dass die personenbezogenen Daten ohne Hinzuziehung zusätzlicher Informationen („Schlüssel") nicht mehr einer spezifischen betroffenen Person zugeordnet werden können. Diese zusätzlichen Informationen werden dabei gesondert aufbewahrt und unterliegen technischen und organisatorischen Maßnahmen, die gewährleisten, dass die personenbezogenen Daten nicht einer identifizierten oder identifizierbaren natürlichen Person zugewiesen werden.

anonymisiert[2]. Ihre Daten werden für 15 Jahre aufbewahrt, um sicher zu stellen, dass die Daten für weiterführende statistische Analysen, z.B. nach Rückfragen aus der Forschungsgemeinschaft, verfügbar sind. Die Daten werden ausschließlich zu Zwecken dieser Studie verwendet.

Sie haben das Recht, vom Verantwortlichen (s.u.) Auskunft über die von Ihnen gespeicherten personenbezogenen Daten zu verlangen. Ebenfalls können Sie die Berichtigung unzutreffender Daten sowie die Löschung der Daten oder Einschränkung deren Verarbeitung verlangen. Der Verantwortliche für die studienbedingte Erhebung personenbezogener Daten ist:

Prof. Dr. med. Jonas Tesarz
Im Neuenheimer Feld 410, 69120 Heidelberg
E-Mail: jonas.tesarz@med.uni-heidelberg.de

Bei Anliegen zur Datenverarbeitung und zur Einhaltung der datenschutzrechtlichen Anforderungen können Sie sich an folgenden Datenschutzbeauftragten der Einrichtung wenden:

Datenschutzbeauftragter des Universitätsklinikum Heidelberg:
Im ZIM / Marsilius-Arkaden Nord
Im Neuenheimer Feld 130.1, 69120 Heidelberg
Telefon: 06221 / 56 7036
E-Mail: Datenschutz@med.uni-heidelberg.de

Im Falle einer rechtswidrigen Datenverarbeitung haben Sie das Recht, sich beifolgender Aufsichtsbehörde zu beschweren:

Der Landesbeauftragte für den Datenschutz und die Informationsfreiheit Baden-Württemberg
Postfach 10 29 32, 70025 Stuttgart
Königstraße 10a, 70173 Stuttgart
Tel.: 0711/61 55 41 – 0, Fax: 0711/61 55 41 – 15
E-Mail: poststelle@lfdi.bwl.de
Internet: http://www.baden-wuerttemberg.datenschutz.de

Freiwilligkeit / Rücktritt

Die Teilnahme an der Fragebogenerhebung erfolgt freiwillig. Falls Sie teilnehmen möchten, bitten wir Sie, die beiliegende Einwilligungserklärung zu unterschreiben. Dort können Sie auch angeben, ob Sie für zukünftige Studienangebote und Folgeuntersuchungen erneut kontaktiert werden dürfen. Sie können diese Einwilligung jederzeit schriftlich oder mündlich ohne Angabe von Gründen widerrufen, ohne dass Ihnen dadurch Nachteile entstehen. Wenn Sie Ihre Einwilligung widerrufen möchten, wenden Sie sich bitte an die Studienleitung oder das Sie behandelnde Personal. Bei einem Widerruf können Sie entscheiden, ob die von Ihnen studienbedingt erhobenen Daten gelöscht werden sollen oder weiterhin für die Zwecke der Studie verwendet

[2] „Anonymisierung" ist das Verändern personenbezogener Daten in der Weise, dass die betroffene Person nicht mehr oder nur mit einem unverhältnismäßig großen Kosten- oder Zeitaufwand identifiziert werden kann.

werden dürfen. Auch wenn Sie einer weiteren Verwendung zunächst zustimmen, können Sie nachträglich Ihre Meinung noch ändern und die Löschung der Daten verlangen; wenden Sie sich dafür bitte ebenfalls an die Studienleitung oder das Sie behandelnde Personal. Beachten Sie, dass Daten, die bereits in wissenschaftliche Auswertungen eingeflossen sind oder Daten, die bereits anonymisiert wurden, nicht mehr auf Ihren Wunsch gelöscht werden können.

Entstehen mir durch die Teilnahme Kosten? / Erhalte ich eine Bezahlung bzw. Aufwandsentschädigung?
Durch die Studienteilnahme entstehen ihnen keine direkten Kosten; möglicherweise können für Sie Fahrtkosten anfallen, wenn Sie mit dem Auto der der Straßenbahn in die Klinik fahren, eine Bezahlung oder Aufwandsentschädigung hierfür ist nicht vorgesehen.

Weitere Informationen
Für weitere Informationen sowie für Auskünfte über allgemeine Ergebnisse und den Ausgang der Studie steht Ihnen als Leiter der Studie Prof. Dr. Jonas Tesarz (Im Neuenheimer Feld 410, 69120 Heidelberg, E-Mail: PostCovid.MED2@med.uni-heidelberg.de) zur Verfügung.

Für Ihre Teilnahme an diesem Forschungsprojekt wären wir Ihnen dankbar!
