# Supplementary material for: Efficacy of supervised immersive virtual reality-based training for the treatment of chronic fatigue in post-COVID syndrome: study protocol for a double-blind randomized controlled trial (IFATICO Trial)
Source: Trials. 2024 Apr 3;25:232. doi: 10.1186/s13063-024-08032-w (PMC10993519; doi:10.1186/s13063-024-08032-w)
Supplement: Supplementary file 7 — Additional file 7. Patient information after randomisation in intervention group. [file 13063_2024_8032_MOESM7_ESM.pdf]

Informationsschrift IFATICO

Lieber Patient, liebe Patientin,

Mit diesem Informationsblatt möchten wir Sie über unsere Behandlung informieren.

Im Zuge der Corona-Pandemie sind zahlreiche Menschen an COVID erkrankt. Während die Krankheit bei einigen Menschen nach der akuten Phase vollständig ausheilt, bleiben die Symptome bei anderen länger bestehen und wieder andere beschreiben, dass die Symptome wieder auftreten, nachdem die Krankheit zunächst ausgeheilt schien. Haben Patient*innen 12 Wochen nach der akuten Infektion eine typische Symptomatik, so spricht man vom Post-COVID-Syndrom. Dazu gehören sowohl mentale, wie auch körperliche teilweise starke Einschränkungen wie Müdigkeit, Konzentrationsschwäche und ein Verschlechtern der Symptome nach körperlicher Belastung („Post Exertional Malaise“). Diese Symptome werden im Folgenden als „chronische Erschöpfung“ zusammengefasst. Aber auch Kurzatmigkeit, Schmerzen, Übelkeit, der Verlust des Geruch- oder Geschmackssinns, Schwindel oder psychische Symptome können dazugehören.

Wie entsteht chronische Erschöpfung?

Bis heute sind die Mechanismen, die zu chronischer Erschöpfung führen nicht genau geklärt, obwohl wir das Symptom von anderen Viruserkrankungen bereits kennen. Vermutet wird jedoch, dass eine Störung der Gehirn-Muskel-Achse von zentraler Bedeutung ist. Die Gehirn-Muskel-Achse ist ein komplexes Kommunikationsnetzwerk zwischen dem Gehirn und den Muskeln unseres Körpers. Dieses Netzwerk spielt eine entscheidende Rolle bei der Regulierung verschiedener Körperfunktionen wie Bewegung, Koordination und Kraft. Die Kommunikation zwischen Gehirn und Muskeln ist für die Aufrechterhaltung der allgemeinen Gesundheit und des Wohlbefindens unerlässlich. Eine Störung dieser Achse kann zu verschiedenen muskulären Störungen wie Muskelschwäche, Spastizität und sogar Lähmungen führen. Es gibt zunehmend Hinweise darauf, dass auch die Post-Covid-assoziierte Müdigkeit auf eine Störung der Gehirn-Muskel-Achse zurückzuführen ist. Werden die Gehirnsignale nicht richtig an die Muskeln weitergeleitet oder sind die Muskeln nicht fähig, auf diese Signale angemessen zu reagieren, kann dies zu verminderter Muskelfunktion und Ermüdung führen. Charakteristisch für diese Erschöpfungssyndrome ist, dass in klinischen Untersuchungen weder eine Schädigung der Muskulatur noch des Gehirns nachgewiesen werden kann, da das Problem nicht in den Organen selbst, sondern in deren gestörtem Zusammenspiel liegt. Darüber hinaus können auch Faktoren wie Überanstrengung, Dehydrierung und unzureichende Ernährung die Gehirn-Muskel-Achse beeinträchtigen und zu Müdigkeit führen. Daher ist es wichtig, die Gehirn-Muskel-Achse durch regelmäßige körperliche Aktivität, richtige Ernährung und ausreichende Ruhezeiten gesund zu erhalten, um Müdigkeit vorzubeugen und zu bewältigen. Auch die Behandlung von Grunderkrankungen und psychischen Problemen kann das Zusammenspiel von Gehirn und Muskeln verbessern und Müdigkeit verringern.

Wieso hilft Trainingstherapie gegen chronische Erschöpfung?

Es ist wichtig, ein gewisses Aktivitätsniveau aufrecht zu erhalten, damit der Körper nicht abbaut, sondern mental und körperlich weiter gefördert wird. Eine Trainingstherapie kann dabei helfen. In

zahlreichen Studien konnte gezeigt werden, dass Trainingstherapie bei Erschöpfung nach Infektionen mit einem Virus helfen kann. Die aktuelle Forschung weist darauf hin, dass dies auch auf die Infektion mit dem Coronavirus zutrifft. Das Gehirn kann durch eine Trainingstherapie neue und positive Erfahrungen mit Sport sammeln, sodass körperliche Betätigung auch nach der Therapie noch positiver gedeutet werden. Wichtig ist dabei, die individuellen Grenzen der Patient*innen nicht zu überschreiten, um eine Verschlechterung der Symptome nach der Belastung zu vermeiden und damit bestehende Erwartungen zu festigen.

Wie funktioniert eine personalisierte Trainingstherapie und warum ist sie wichtig für die Behandlung?

Um eine Überforderung zu vermeiden, wird die Intensität des Trainings bei einer personalisierten Trainingstherapie an die individuellen Möglichkeiten der Patient*innen angepasst. In unserem Fall bedeutet das konkret, dass wir gemeinsam mit Ihnen herauszufinden versuchen, wo genau Ihre kritische Belastungstoleranzschwelle liegt. Also diejenigen Grenze, ab welcher eine Belastung bei Ihnen zu einer Verschlechterung Ihrer Symptome führt. Die Therapie wird dann entsprechend ihrer persönlichen Belastungsgrenze angepasst, bzw. „personalisiert“, so dass das Training stets unterhalb der persönlichen Belastungsgrenze erfolgen und eine Überlastung vermieden werden kann. Wir werden Sie daher im Rahmen des Trainingsprogramms regelmäßig fragen, wie es Ihnen nach der letzten Trainingssession ergangen ist und das Programm gegebenenfalls daran anpassen. Unsere Therapie richtet sich dabei nach einem spezifischen Schema mit verschiedenen Trainingsstufen, die aufeinander abgestimmt sind und zwischen denen flexibel hin und her gewechselt werden kann. Hat sich Ihr Körper und ihr Nervensystem an eine Belastungsstufe angepasst, so können Sie das nächste Mal auf einer höheren Stufe trainieren. Ging es Ihnen hingegen nach einem Training weniger gut, so werden wir das Pensum reduzieren.

Was erwartet mich während der Behandlung und mit wieviel Zeitaufwand muss ich rechnen?

Die Trainingstherapie läuft über 6 Wochen, für jede Woche sind zwei Termine vorgesehen. Pro Termin sollten Sie dabei circa 1h einplanen. Was Sie dabei ganz konkret erwartet, hängt davon ab, zu welchem Therapieprogramm dieser Studie Sie zugewiesen werden. In allen Therapieprogrammen werden Sie eine personalisierte Trainingstherapie bekommen, nur die technische Umsetzung zwischen den Studienarmen unterscheidet sich. Wichtig ist, dass Sie möglichst alle zwölf Termine wahrnehmen, da die Behandlung dann die bestmögliche Aussicht auf Erfolg hat.

Außerdem wird vor und nach der Intervention ein Assessment durchgeführt bei dem verschiedene klinische Tests mit Ihnen gemacht werden (Konzentrationstests, Kraft- und Ausdauertest). Dafür sollten Sie jeweils eine halbe Stunde Zeit einplanen. Zusätzlich bekommen Sie vor und nach der Intervention einen Fragebogen zugesandt, dessen Beantwortung circa 30-40min dauert. Bitte beachten Sie, dass aus statistischen Gründen auch dann ein Assessment durchgeführt und der Fragebogen beantwortet werden soll, wenn Sie sich dazu entscheiden, die Studie abzubrechen. Drei und zwölf Monate nach der Intervention werden wir Ihnen noch einmal einen Fragebogen zuschicken, für den Sie 10-15min Zeit einplanen sollten.

Was bedeutet Zuweisung in diesem Zusammenhang?

In klinischen Studien weist man Patient*innen in der Regel einer Behandlung oder einer Kontrollgruppe zu. Im Rahmen von IFATICO legen wir großen Wert darauf, dass alle unsere Patient*innen eine effiziente Therapie erhalten. Wir vergleichen im Rahmen dieser Studie deswegen zwei unterschiedliche technische Umsetzungen einer personalisierten Trainingstherapie und teilen Sie zufällig einer der beiden Gruppen zu. Für die Auswertung der Studie ist es dabei wichtig, dass Sie nicht wissen, welche Behandlung die andere Gruppe bekommen hat. Wir bitten Sie deswegen, die Erfahrungen, die Sie machen, nicht mit anderen Studienteilnehmern zu teilen.

Virtual Reality Trainingstherapie

Ein vielversprechender neuer Ansatz in der medizinischen Forschung ist die Behandlung mit Virtual Reality gestützten Therapien. Dass solche Therapien helfen können, wurde bereits für verschiedenste Bereiche gezeigt, zum Beispiel in der Schmerzmedizin, in der Psychiatrie oder bei Patient*innen, die sich von einem Schlaganfall erholen.

In unserem Fall haben Patient*innen die Möglichkeit, eine Trainingstherapie auf dem Gerät ICAROS Health zu absolvieren. Das Gerät ist ein Medizinprodukt und die Benutzung ist sicher. Wir werden Sie außerdem während der einzelnen Termine stets 1:1 persönlich betreuen und stehen jederzeit bereit, wenn Sie den Eindruck haben, es geht Ihnen nicht gut.

Zusätzlich werden Sie gebeten, auch in Ihren Alltag nach Möglichkeit Bewegung einzubauen. Dafür bieten sich zum Beispiel Spaziergänge an oder andere Aktivitäten, die Sie gut dosieren können. Auch hier gilt, dass Sie bis zu dem gemeinsam erarbeiteten Erschöpfungsniveau trainieren sollten und nicht darüber hinaus. Wenn Sie über eine Woche keine Verschlechterung Ihrer Symptome verspüren, können Sie die Trainingszeit probehalber erhöhen, am besten in 30-90s Schritten. Führt also ein zehnminütiger Spaziergang eine Woche lang zu keiner Verschlechterung, so können Sie es einmal mit einem elfminütigen Spaziergang probieren.

Wie läuft eine Virtual Reality Trainingstherapie ab?

Das Kernelement der Virtual Reality basierten Trainingstherapie ist die neuromuskuläre Aktivierung mittels eines speziellen Trainingsgeräts, welches virtuelle Realität mit einem realem neuromuskulären Koordinationstraining kombiniert. Dabei gleicht das Gerät auf dem ersten Blick einer Art „Flugsimulator“: Man „legt“ sich in das Gerät und stützt sich dabei auf den Unterschenkeln, dem Bauch und den Unterarmen ab. Gleichzeitig wird man mittels einer hochauflösenden Virtual Reality Brille und einem speziellem Computersimulator eine virtuelle Realität eingeführt, welche bereits über kleinste Körperbewegungen gesteuert werden kann. Indem man den Körperschwerpunkt leicht in die eine oder andere Richtung verlagert, kippt das Gerät dementsprechend in die ein oder andere Richtung. Über die Virtual Reality Brille wird gleichzeitig das das Erleben hervorgerufen, über eine Landschaft zu fliegen und bestimmt die Richtung des Fluges über das Verlagern des Körpers. Um Sie nicht zu überfordern, werden wir Sie langsam an diese Erfahrung gewöhnen. Das Trainingsprogramm ist dabei bewusst „spielerisch“ gestaltet, um einer frühzeitigen Ermüdung vorzubeugen. Sie können sich daher aus einem Repertoire unterschiedlicher spielerischer Aktivierungsprogramme individuell ein für Sie passendes virtuelles Modul heraussuchen, welches Sie im Verlauf auch jederzeit wieder

wechseln können. In den Ihnen im Rahmen der Trainingsmodulen angebotenen unterschiedlichen virtuellen Welt gilt es dann unterschiedliche Aufgaben zu bewältigen, wie zum Beispiel in einer virtuellen Welt durch einen Parkour zu fliegen und Gegenstände einzusammeln. Dabei werden wir den Grad der erforderlichen Beweglichkeit, die Anforderung an die neuromuskuläre Koordination und den Schwierigkeitsgrad der zu bewältigenden Aufgaben individuell an Ihre Bedürfnisse anpassen. Die Anpassung erfolgt dabei so, dass Sie stets unterhalb Ihrer individuellen Belastungstoleranzgrenze bleiben können. Zum Training gehört außerdem ein kurzes Aufwärmen, Übungen für Kraft und Koordination und nach jedem Training das Auslockern und Dehnen der Muskeln. Dabei werden Sie stets 1:1 betreut. Sie müssen sich keine Sorgen machen, dass Sie die Therapie nicht schaffen.

Worauf beruht die Wirkung?

Ein neuer und viel versprechender Ansatz zur Stärkung der Gehirn-Muskel-Achse durch die Simulation einer intakten Körperfunktion ist das Training in virtueller Realität (Virtual Reality, VR). Durch den Einsatz von VR können Menschen Aktivitäten ausführen, die reale Bewegungen imitieren und die Gehirn-Muskel-Achse stimulieren, was zu einer Verbesserung der Muskelfunktion und zu einer Verringerung der Ermüdung führen kann, ohne dass es zur gleichen Zeit zu Erschöpfungssymptomen kommt. Studien haben gezeigt, dass VR-Training Menschen mit neurologischen Störungen wie Schlaganfall, traumatischen Hirnverletzungen und Multipler Sklerose helfen kann, ihre motorischen Fähigkeiten zu verbessern und Muskelschwäche und Müdigkeit zu reduzieren. VR-Training kann auch Sportlern und Menschen in der Physiotherapie helfen, bestimmte Muskeln und Bewegungen zu trainieren, was zu einer verbesserten Muskelfunktion und weniger Ermüdung führt. Durch die Simulation einer intakten Körperfunktion unterstützt die virtuelle Realität das Gehirn bei der Wiederherstellung der Gehirn-Muskel-Achse, verbessert die Muskelfunktion und verringert die Ermüdung. Die Weiterentwicklung dieser Technologie hat das Potenzial, die Art und Weise zu revolutionieren, wie wir Menschen mit muskulären Störungen trainieren und rehabilitieren.

Welche Risiken hat die Therapie?

Wie jede Trainingstherapie birgt auch die Virtual Reality Trainingstherapie die Gefahr, dass sich die Symptome nach der Belastung verstärken. Beim Design der Therapie haben wir aber größten Wert daraufgelegt, dieses Risiko zu minimieren. Sollten Sie sich doch einmal nach dem Training schlechter fühlen, so werden wir die Therapie umgehend anpassen.

Die Behandlung mit den Virtual Reality Brillen kann zu Schwindel, Übelkeit oder Kopfschmerzen führen. Diese Symptome sind vorübergehend und selbstverständlich können Sie die Behandlung jederzeit abbrechen oder pausieren, sollte es Ihnen nicht gut gehen. Außerdem stellen wir das ICAROS Health Gerät so ein, dass die Auslenkung gering ist und Sie schnell Kontrolle über das Gerät gewinnen. Auch könnten Sie negative Erfahrungen sammeln, insbesondere wenn Sie die virtuelle Welt als überfordernd wahrnehmen. Die ersten „Flüge“ auf dem Gerät werden deswegen ohne Brille stattfinden und erst wenn Sie sich wohlfühlen, können Sie ganz eintauchen. So können Sie sich in Ihrem Tempo an die Erfahrung gewöhnen.

FAQ

1. ### Wie werden die Termine ausgemacht?

   Die Vereinbarung von Terminen mit unserem Studienpersonal kann telefonisch stattfinden unter +49 163 1450412 oder per E-Mail: PostCovid.MED2@med.uni-heidelberg.de.

2. ### Wo findet das Training statt?

   Das Training findet in der Medizinischen Klinik in Heidelberg in der Psychosomatischen Ambulanz statt (Im Neuenheimer Feld INF 410). Die genauen Raumdaten klären wir mit Ihnen während der Terminvergabe.

3. ### Was passiert, wenn ich einen Termin nicht wahrnehmen kann?

   Falls Sie einen Termin nicht wahrnehmen können, so halten Sie bitte Rücksprache mit Ihrem Therapeuten oder Ihrer Therapeutin, gegebenenfalls ist eine Verschiebung Ihres Termins möglich.

4. ### Was passiert, wenn ich mehrmals fehle?

   Die Trainingstherapie lebt von Ihrer regelmäßigen Teilnahme. Bitte prüfen Sie vorab Ihre zeitlichen Ressourcen und fehlen Sie nicht öfter als zweimal. Wenn Sie während der Therapie feststellen, dass Sie mehrere Trainings nicht wahrnehmen können, so halten Sie bitte möglichst frühzeitig Rücksprache mit Ihrem Therapeuten oder Ihrer Therapeutin, damit wir für Sie andere Termine planen können.

5. ### Wie sollte ich mich auf das Training vorbereiten?

   Zur optimalen Vorbereitung auf das Training empfehlen wir, dass Sie viel Wasser trinken und in der Stunde vor dem Termin nichts mehr essen. Kommen Sie außerdem gerne in lockerer und bequemer Kleidung.

6. ### Kann ich Schäden von der Behandlung davontragen?

   Wir erwarten nicht, dass Sie langfristige Schäden davontragen. Es kann sein, dass Sie während dem Training oder danach eine verstärkte Erschöpfung empfinden. Sollte das während dem Training passieren, so sagen Sie uns bitte Bescheid, dann pausieren wir das Training oder brechen es ab. Bemerken Sie nach dem Training, dass es Ihnen schlechter geht als sonst, so informieren Sie uns bitte auch, dann passen wir das nächste Training daran an.

7. ### Was passiert, wenn Ich das Training nicht schaffe?

   Sie müssen sich keine Sorgen machen, dass Sie das Training nicht schaffen. Wir werden die Intensität an Ihre Möglichkeiten anpassen. Wenn Ihnen eine Übung zu schwierig ist oder Sie weniger Wiederholungen schaffen als geplant, so werden wir Übung und Wiederholungen leichter gestalten.

8. ### Was passiert, wenn ich anschließend an eine Sitzung eine Verschlechterung meiner Symptome verspüre?

   Wenn sich durch das Training Ihre Symptome verschlechtern, so sagen Sie uns bitte Bescheid! Wir werden das nächste Training dann anpassen.

   Brauchen Sie zu Hause akute Hilfe, so können Sie sich an den diensthabenden Arzt oder Ärztin in der Psychosomatik wenden.

9. Was passiert, wenn ich anschließend an Bewegung im Alltag eine Verschlechterung meiner Symptome verspüre?

Informieren Sie uns bitte auch, wenn die Bewegung zu Hause zu einer Verschlechterung der Symptome führt. Ihr Therapeut oder Ihre Therapeutin kann dann mit Ihnen besprechen, ob das Training reduziert werden muss oder ob es alternative Formen der Bewegung gibt, die zu Ihnen besser passt.

10. Können meine engste Familie und Freunde über die Erfahrungen in der Studie informiert werden?

Selbstverständlich können Sie Ihre Erfahrung mit Familie und Freunden teilen.

11. Darf ich mit anderen Studienteilnehmern über die Studie reden?

Diese Studie strebt ein verblindetes Design an, das bedeutet, die Teilnehmer sollen nicht wissen, in welche Gruppe sie eingeteilt wurden und welche Intervention die andere Gruppe macht. Das ist wichtig, damit die eigenen Meinungen der Teilnehmer über das Trainingsprogramm das Ergebnis der Studie nicht verfälschen. Sie dürfen also anderen Studienteilnehmern erzählen, dass Sie an der Studie teilnehmen aber nicht verraten, wie Ihr Trainingsprogramm abläuft.

12. Darf ich den Studienpersonal, die meine Daten erheben, erzählen, in welcher Gruppe ich war?

Auch das Studienpersonal, welches nicht an der Therapie beteiligt ist (z.Bsp. die Personen, die die Fragebögen erheben oder klinische Tests mit Ihnen durchführen), sollten in unserer Studie verblindet sein, also nicht wissen, welcher Gruppe Sie angehören. Das ist wichtig, weil auch die Erwartung dieser Personen das Ergebnis der Studie verfälschen könnte. Das bezieht sich selbstverständlich nur auf die Personen, die während und nach der Studie Ihre Daten erheben, also mit Ihnen über die Fragebögen sprechen oder klinische Tests machen. Die Betreuer während des Trainingsprogramms wissen natürlich, in welcher Gruppe Sie sind. Mit ihnen können Sie alles besprechen und sie alles fragen.

13. Darf mein Hausarzt informiert werden?

Auch Ihr Hausarzt oder Hausärztin darf natürlich gerne informiert werden. Wenn Sie es wünschen, können wir auch Daten an Ihre Hausarztpraxis übermitteln.

14. Gibt es die Möglichkeit, die Therapie nach Ende der Studie weiterzuführen?

Bisher werden die von uns angebotenen Trainingsprogramme noch nicht in der allgemeinen Versorgung angeboten. Sollten Sie am Ende der Studie das Gefühl heben, das Trainingsprogramm sei nicht ausreichend gewesen und Sie würden es gerne noch selbstständig fortführen, so sprechen Sie gerne einen unserer Therapeuten an, welche Möglichkeiten es für Sie gibt, das gelernte und erreichte individuell selbstständig fortzuführen.
