# Supplementary material for: Efficacy of supervised immersive virtual reality-based training for the treatment of chronic fatigue in post-COVID syndrome: study protocol for a double-blind randomized controlled trial (IFATICO Trial)
Source: Trials. 2024 Apr 3;25:232. doi: 10.1186/s13063-024-08032-w (PMC10993519; doi:10.1186/s13063-024-08032-w)
Supplement: Supplementary file 9 — Additional file 9. CONSORT-Statement for Randomized Trials of Nonpharmacologic treatments (2017). [file 13063_2024_8032_MOESM9_ESM.pdf]

 Annals of Internal Medicine

CONSORT Statement for Randomized Trials of Nonpharmacologic Treatments: A 2017 Update and a CONSORT Extension for Nonpharmacologic Trial Abstracts

Isabelle Boutron, MD, PhD; Douglas G. Altman, DSc; David Moher, PhD; Kenneth F. Schulz, PhD, MBA; and Philippe Ravaud, MD, PhD, for the CONSORT NPT Group*

Incomplete and inadequate reporting is an avoidable waste that reduces the usefulness of research. The CONSORT (Consolidated Standards of Reporting Trials) Statement is an evidence-based reporting guideline that aims to improve research transparency and reduce waste. In 2008, the CONSORT Group developed an extension to the original statement that addressed methodological issues specific to trials of nonpharmacologic treatments (NPTs), such as surgery, rehabilitation, or psychotherapy. This article describes an update of that extension and presents an extension for reporting abstracts of NPT trials. To develop these materials, the authors reviewed pertinent literature published up to July 2016; surveyed authors of NPT trials; and conducted a consensus meeting with editors, trialists, and methodologists.

Changes to the CONSORT Statement extension for NPT trials include wording modifications to improve readers' understanding and the addition of 3 new items. These items address whether and how adherence of participants to interventions is assessed or enhanced, description of attempts to limit bias if blinding is not possible, and specification of the delay between randomization and initiation of the intervention. The CONSORT extension for abstracts of NPT trials includes 2 new items that were not specified in the original CONSORT Statement for abstracts. The first addresses reporting of eligibility criteria for centers where the intervention is performed and for care providers. The second addresses reporting of important changes to the intervention versus what was planned. Both the updated CONSORT extension for NPT trials and the CONSORT extension for NPT trial abstracts should help authors, editors, and peer reviewers improve the transparency of NPT trial reports.

Ann Intern Med. 2017;167:40-47. doi:10.7326/M17-0046 **Annals.org**
For author affiliations, see end of text.
This article was published at Annals.org on 20 June 2017.
* For a list of members of the CONSORT NPT Group, see **Appendix 1** (available at Annals.org).

Incomplete reporting is responsible for a great deal of avoidable waste in research (1, 2). The CONSORT (Consolidated Standards of Reporting Trials) Statement (3–5), an evidence-based reporting guideline, was developed to improve research transparency.

Nonpharmacologic treatments (NPTs), such as surgery, rehabilitation, education, psychotherapy, and devices, represent a wide range of interventions. However, assessing NPTs raises specific methodological issues related to the complexity of the intervention, the influence of care providers, the expertise of the center, and the difficulties of blinding (6–14). To account for these issues, the CONSORT Group developed a CONSORT Statement extension for trials of NPTs ("CONSORT NPT extension"), which was published in *Annals of Internal Medicine* in 2008 (15, 16).

In 2010, the main CONSORT Statement was updated (5, 17). To account for this update and methodological developments since publication of the original NPT extension, we aimed to update the CONSORT NPT extension and develop a CONSORT extension for reporting abstracts of NPT trials (18, 19).

METHODS

We updated the CONSORT NPT extension in 3 steps. First, we reviewed the literature to identify up-to-date evidence. The search is detailed in **Appendix 2** (available at Annals.org). Second, we surveyed corresponding authors of published articles citing the 2008 CONSORT NPT extension. Of the 1525 authors invited by e-mail, 194 (13%) participated. For each item of the CONSORT NPT extension, participants were asked to indicate whether they believed the item should be modified and, if so, why and how. The results of the survey are reported in **Appendix Tables 1** and **2** (available at Annals.org). From the literature review and the survey, we synthesized proposals for changes to each item. Finally, we organized a 2-day consensus meeting in May 2014 in Paris, France, with 22 participants (9 editors, 6 trialists, and 7 methodologists). During this meeting, the survey results and proposals for change were presented and each item was discussed until consensus was reached. After the meeting, we developed a draft of the current manuscript, which was sent to all participants for comments. The updated checklist was not modified at this stage.

Updating the CONSORT NPT Extension

Main Changes to the CONSORT NPT Extension

The revision of the CONSORT NPT extension checklist consisted of the deletion of items, the addition of new items, wording changes, and reformatting. The numbering and content of items were adjusted to follow the 2010 CONSORT Statement. Some wording was changed to improve readers' understanding, such as the use of "care providers" instead of "those performing the intervention" in item 3.

Items 11a and 11b, related to blinding, were modified because they were incorporated into the 2010 CONSORT Statement. Three new items were added to account for the difficulties in replicating NPTs, the frequent lack of blinding, and the risk for a differential

 © 2017 American College of Physicians

Downloaded from https://annals.org by Univ of North Carolina user on 08/13/2019

Table 1. 2017 CONSORT Checklist of Information to Include When Reporting Randomized Trials Assessing NPTs*

Checklist Item Number, by Section/Topic Item	CONSORT Item	Extension for NPT Trials
**Title and abstract**		
1a	Identification as a randomized trial in the title	–
1b	Structured summary of trial design, methods, results, and conclusions (for specific guidance see CONSORT for abstracts)	Refer to CONSORT extension for abstracts for NPT trials
**Introduction**		
Background and objectives		
2a	Scientific background and explanation of rationale	–
2b	Specific objectives or hypotheses	–
**Methods**		
Trial design		
3a	Description of trial design (such as parallel, factorial) including allocation ratio	When applicable, how care providers were allocated to each trial group
3b	Important changes to methods after trial commencement (such as eligibility criteria), with reasons	–
Participants		
4a	Eligibility criteria for participants	When applicable, eligibility criteria for centers and for **care providers**
4b	Settings and locations where the data were collected	–
Interventions†		
5	The interventions for each group with sufficient details to allow replication, including how and when they were actually administered	Precise details of both the experimental treatment and comparator
5a		Description of the different components of the interventions and, when applicable, description of the procedure for tailoring the interventions to individual participants.
5b		Details **of whether and** how the interventions were standardized.
5c		Details **of whether and** how adherence of care providers to the protocol was assessed or enhanced
**5d**		**Details of whether and how adherence of participants to interventions was assessed or enhanced**
Outcomes		
6a	Completely defined pre-specified primary and secondary outcome measures, including how and when they were assessed	–
6b	Any changes to trial outcomes after the trial commenced, with reasons	–
Sample size		
7a	How sample size was determined	When applicable, details of whether and how the clustering by care providers or centers was addressed
7b	When applicable, explanation of any interim analyses and stopping guidelines	–
Randomization		
Sequence generation		
8a	Method used to generate the random allocation sequence	–
8b	Type of randomization; details of any restriction (such as blocking and block size)	–
Allocation concealment mechanism		
9	Mechanism used to implement the random allocation sequence (such as sequentially numbered containers), describing any steps taken to conceal the sequence until interventions were assigned	–
Implementation		
10	Who generated the random allocation sequence, who enrolled participants, and who assigned participants to interventions	–
Blinding		
11a	If done, who was blinded after assignment to interventions (for example, participants, care providers, those assessing outcomes) and how	If done, who was blinded after assignment to interventions (e.g., participants, care providers, **those administering co-interventions,** those assessing outcomes) and how
11b	If relevant, description of the similarity of interventions	
**11c**		**If blinding was not possible, description of any attempts to limit bias**

Continued on following page

Downloaded from https://annals.org by Univ of North Carolina user on 08/13/2019

Table 1–Continued

Checklist Item Number, by Section/Topic Item	CONSORT Item	Extension for NPT Trials
Statistical methods		
12a	Statistical methods used to compare groups for primary and secondary outcomes	When applicable, details of whether and how the clustering by care providers or centers was addressed
12b	Methods for additional analyses, such as subgroup analyses and adjusted analyses	–
**Results**		
Participant flow (a diagram is strongly recommended)		
13a	For each group, the numbers of participants who were randomly assigned, received intended treatment, and were analyzed for the primary outcome	The number of care providers or centers performing the intervention in each group and the number of patients treated by each care provider or in each center
13b	For each group, losses and exclusions after randomization, together with reasons	–
13c		**For each group, the delay between randomization and the initiation of the intervention**
New		Details of the experimental treatment and comparator as they were implemented
Recruitment		
14a	Dates defining the periods of recruitment and follow-up	–
14b	Why the trial ended or was stopped	–
Baseline data		
15	A table showing baseline demographic and clinical characteristics for each group	When applicable, a description of care providers (case volume, qualification, expertise, etc.) and centers (volume) in each group
Numbers analyzed		
16	For each group, number of participants (denominator) included in each analysis and whether the analysis was by original assigned groups	–
Outcomes and estimation		
17a	For each primary and secondary outcome, results for each group, and the estimated effect size and its precision (such as 95% confidence interval)	–
17b	For binary outcomes, presentation of both absolute and relative effect sizes is recommended	–
Ancillary analyses		
18	Results of any other analyses performed, including subgroup analyses and adjusted analyses, distinguishing pre-specified from exploratory	–
Harms		
19	All important harms or unintended effects in each group (for specific guidance see CONSORT for harms)	–
**Discussion**		
Limitations		
20	Trial limitations, addressing sources of potential bias, imprecision, and, if relevant, multiplicity of analyses	In addition, take into account the choice of the comparator, lack of or partial blinding, and unequal expertise of care providers or centers in each group
Generalizability		
21	Generalizability (external validity, applicability) of the trial findings	Generalizability (external validity) of the trial findings according to the intervention, comparators, patients, and care providers and centers involved in the trial
Interpretation		
22	Interpretation consistent with results, balancing benefits and harms, and considering other relevant evidence	–
**Other information**		
Registration		
23	Registration number and name of trial registry	–
Protocol		
24	Where the full trial protocol can be accessed, if available	–
Funding		
25	Sources of funding and other support (such as supply of drugs), role of funders	–

CONSORT = Consolidated Standards of Reporting Trials; NPT = nonpharmacologic treatment.
* Additions or modifications to the 2010 CONSORT checklist. Modifications of the extension are in boldface.
† These items are consistent with the Template for Intervention Description and Replication (TIDieR) checklist.

Downloaded from https://annals.org by Univ of North Carolina user on 08/13/2019

delay between randomization and initiation of the intervention. These items are dedicated to whether and how adherence of participants to interventions is assessed or enhanced (item 5d), the description of any attempts to limit bias if blinding is not possible (item 11c), and the delay between randomization and initiation of the intervention (item 13c).

The updated NPT checklist is shown in **Table 1**, with examples of adequate reporting in **Appendix Table 3** and **Appendix Figures 1** and **2** (available at Annals.org). The modified participant flow diagram is presented in the **Figure**.

Development of the CONSORT Extension for Reporting Abstracts of NPT Trials

The CONSORT extension for abstracts was published in 2008 (18). We added 2 new items to this extension: one for reporting "eligibility criteria for centers where the intervention is performed and for care providers", and one for "any important changes to the intervention delivered from what was planned" (**Table 2**). **Appendix Table 4** (available at Annals.org) provides examples of published abstracts that we modified to adhere to the CONSORT extension for abstracts of NPT trials.

Specific Methodological Issues Considered in the Update to the CONSORT NPT Extension

Complexity of NPTs. Nonpharmacologic treatments frequently involve multicomponent interventions delivered by multiple care providers, and each component or provider may influence the success of the overall intervention (20). Nonpharmacologic treatments are difficult to describe and standardize, and the "active ingredients" are sometimes difficult to disentangle (21). Furthermore, the intervention that is actually administered may differ substantially from the one that was planned.

To account for these issues, the updated CONSORT NPT extension recommends providing a description of the components of the intervention and, when applicable, a description of the procedure for tailoring the intervention to individual participants (item 5a) in the methods section. We also recommend describing whether and how the interventions were standardized (item 5b), whether and how adherence of care providers to the protocol was assessed or enhanced (item 5c), and whether and how adherence of participants to interventions was assessed or enhanced (item 5d).

In the results section, authors should report details of the experimental treatment and comparator as they were implemented (new item). In the abstract, authors should report "any important changes to the intervention delivered from what was planned".

These items are consistent with the Template for Intervention Description and Replication (TIDieR) checklist and guidance (22).

Influence of Center and Care Provider Expertise. For most NPT trials, the volume of the center providing the intervention and the expertise of the care providers can greatly affect estimates of treatment effect. Interventions that are beneficial in one setting may be less effective or even harmful in another setting (23). Furthermore, in NPT trials comparing interventions that could be performed by the same care provider (for example, surgical procedures), different methods for allocating care providers to each group are possible—care providers can deliver the intervention in both groups or only 1 group. All of these choices can raise specific methodological and logistical issues (**Appendix Table 5**, available at Annals.org).

The updated CONSORT NPT extension recommends reporting how care providers were allocated to each trial group (item 3a), eligibility criteria for centers and care providers (item 4a), the number of care providers or centers performing each intervention and the number of patients treated by each care provider or in each center (item 13a), and a description of care providers (for example, case volume, qualification, and expertise) and centers (volume) in each group (item 15).

Furthermore, the flow diagram (item 13a) includes a supplementary box to report the number of care providers or centers performing the intervention in each treatment group and the number of patients treated by each care provider or in each center (**Figure**). The flow diagram should report summary statistics, but the detailed description of the number of patients included and treated in each center and each group could be reported in an appendix. This information is particularly important because the interpretation and applicability of the results vary considerably if, for example, 1 high-volume surgeon in 1 high-volume center performs 90% of the interventions or if the interventions are well-distributed in all centers and among all surgeons. Finally, authors should discuss the limitations related to any differing expertise of care providers or centers in each group (item 20) and the generalizability according to the care providers and centers involved in the trial (item 21). In the abstract, we also recommend reporting eligibility criteria for care providers and centers where the intervention is performed.

Clustering. In individual randomized controlled trials (RCTs), standard sample size calculations and statistical analyses assume that the outcome for each participant is independent. However, this may not be true in individual NPT RCTs in which the outcomes tend to be more similar for patients treated by the same care provider than by other care providers (24). Lack of accounting for this type of clustering by care providers and centers may lead to an underestimation of the sample size required and result in imprecision (25–27). Many analysis methods, such as fixed-, random-, or mixed-effects models and generalized estimating equations, are available to account for clustering (28–30).

The updated CONSORT NPT extension recommends reporting details of whether and how the clustering by care providers or centers was addressed in the sample size calculation (item 7a) and the statistical analysis (item 12a).

Difficulties of Blinding. Blinding of patients and care providers is frequently impossible in trials assessing NPTs and often relies on complex methods when it

Downloaded from https://annals.org by Univ of North Carolina user on 08/13/2019

Figure. Modified CONSORT flow diagram for individual randomized controlled trials of nonpharmacologic treatments.

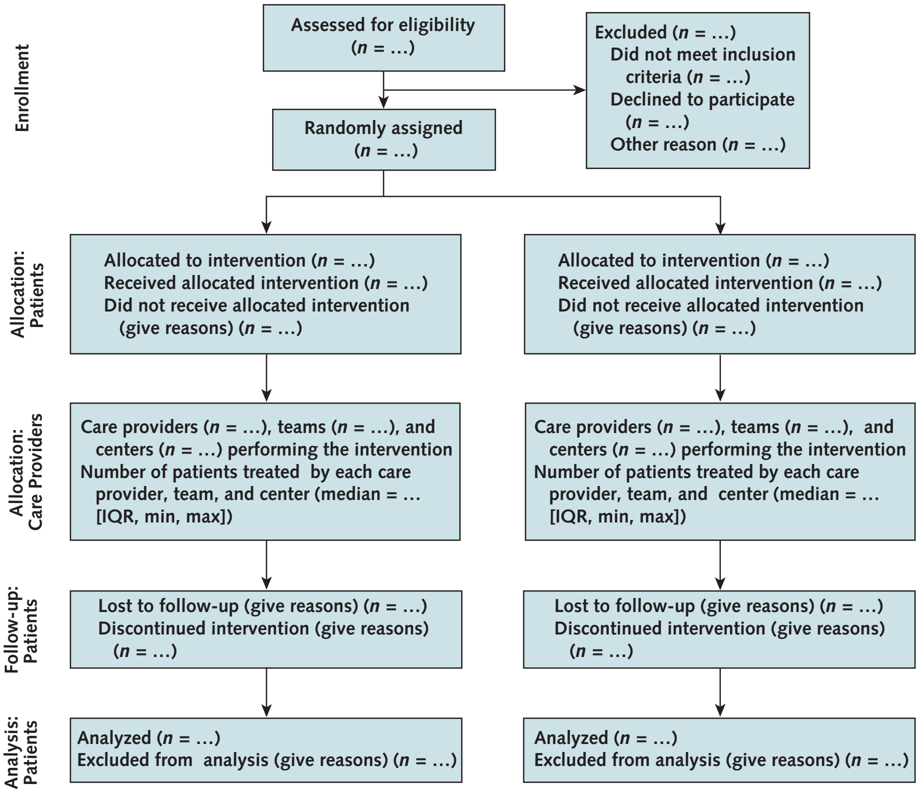

An extra box relating to care providers and centers has been added for each intervention group. CONSORT = Consolidated Standards of Reporting Trials; IQR = interquartile range; max = maximum; min = minimum.

is possible (9, 31). Some of these methods rely on blinding other care providers who do not perform the intervention but who will care for the patients after the intervention. To account for this, item 11a was modified slightly.

If blinding is not possible in a trial, the updated CONSORT NPT extension recommends reporting this information explicitly and providing a description of any attempts to limit bias, such as collection of data by an independent researcher (item 11c). Furthermore, the authors are advised to discuss the limitations related to the lack of blinding when relevant (item 20).

Delay Between Randomization and Initiation of the Intervention. For most NPT RCTs, a delay may occur between randomization and delivery of the intervention (32). This delay is typically related to logistical issues owing, for example, to scheduling hospitalizations or visits with the care provider (33). Such a delay could lead to crossover, loss to follow-up, or nonadherence to the intervention if the participant's status changes between randomization and intervention. The delays can be unequal between trial groups; for example, a trial comparing chemonucleolysis and manipulation in patients with lumbar disc herniation had an average delay of 3 weeks in one group and 13 weeks in the other group (34). The updated CONSORT NPT extension recommends reporting "for each group, the delay between randomization and initiation of the intervention" (item 13c) in the results section.

Adherence to the Original CONSORT NPT Extension

Several systematic reviews showed poor adherence to the original CONSORT NPT extension. Only 39% of NPT interventions were adequately described in NPT trial reports (35). A systematic evaluation of surgical RCTs showed that only 6% and 4% reported how clustering was addressed in the sample size calculation and statistical analysis, respectively (36).

DISCUSSION

The updated CONSORT NPT extension should enable authors to increase the transparency of their reports and facilitate an accurate interpretation of trial results. Improving transparency is particularly important in the context of the replication crisis in science (37).

Despite the publication of the CONSORT Statement extension to NPT trials, the completeness of reporting of NPT trials remains insufficient (38–40). Space constraints in published articles has been suggested as one reason for inadequate reporting of interventions; however, an online appendix or a link to a Web site that provides access to videos and manuals can be used to report this essential information. Open-access repositories, such as the Open Science Framework (https://osf.io), should facilitate dissemination of this information.

Downloaded from https://annals.org by Univ of North Carolina user on 08/13/2019

Table 2. Items to Include When Reporting RCTs Assessing NPTs in a Journal or Conference Abstract*

Item	Standard CONSORT Abstract Item	Extension for NPT Trials
Title	Identification of the study as randomized	–
Authors	Contact details for the corresponding author	–
Trial design	Description of the trial design (e.g. parallel, cluster, noninferiority)	–
Methods		
Participants	Eligibility criteria for participants and the settings where the data were collected	When applicable, report eligibility criteria for centers where the intervention is performed and for care providers
Interventions	Interventions intended for each group	–
Objective	Specific objective or hypothesis	–
Outcome	Clearly defined primary outcome for this report	–
Randomization	How participants were allocated to interventions	–
Blinding (masking)	Whether or not participants, care givers, and those assessing the outcomes were blinded to group assignment	–
Results		
Number randomly assigned	Number of participants randomized to each group	–
Recruitment	Trial status	–
		Report any important changes to the intervention delivered from what was planned
Number analyzed	Number of participants analyzed in each group	–
Outcome	For the primary outcome, a result for each group and the estimated effect size and its precision	–
Harms	Important adverse events or side effects	–
Conclusions	General interpretation of the results	–
Trial registration	Registration number and name of trial register	–
Funding	Source of funding	–

CONSORT = Consolidated Standards of Reporting Trials; NPT = nonpharmacologic treatment; RCT = randomized controlled trial.

Several initiatives to improve adherence to guidelines have already been implemented (41), most during the submission and peer review process. More recently, use of an online writing aid based on the CONSORT guidelines has shown promising results (42).

The process for developing these guidelines followed recommended practices (43). The updated checklist resulted from a consensus, and some specific issues that were discussed during the meeting, such as difficulties in recruiting in NPT RCTs because of strong investigator and patient preferences, did not lead to changes in the checklist.

Some of the issues considered in this extension can be applied more broadly to the reporting of RCTs assessing such pharmacologic treatments as complex chemotherapy. Finally, the updated checklist is consistent with reporting guidelines that were developed after publication of the 2008 CONSORT extension for NPTs, particularly the TIDieR checklist for better reporting of interventions (22).

We hope the 2017 update of the CONSORT NPT extension improves the reporting of RCTs. The guidelines are not intended to deter authors from publishing imperfect trials—the perfect trial being difficult to achieve—but to ensure transparency and a coherent approach to testing and reporting trials of complex interventions.

From Paris Descartes University, INSERM UMR1153, and Assistance Publique des Hôpitaux de Paris, Paris, France; University of Oxford, Oxford, United Kingdom; Ottawa Hospital Research Institute and University of Ottawa, Ottawa, Ontario, Canada; FHI 360, Durham, North Carolina; University of North Carolina, Chapel Hill, North Carolina; and Columbia University Mailman School of Public Health, New York, New York.

Financial Support: Funding was provided by the research funds of the Centre d'Epidémiologie Clinique, Assistance Publique des Hôpitaux de Paris, and the METHODS Team of the Centre of Research in Epidemiology and Statistics Sorbonne Paris Cité – CRESS-UMR1153.

Disclosures: Dr. Ravaud reports that he is a member of the CONSORT Group, director of EQUATOR France, and a member of the EQUATOR steering group. Authors not named here have disclosed no conflicts of interest. Disclosures can also be viewed at www.acponline.org/authors/icmje/ConflictOfInterestForms.do?msNum=M17-0046.

Requests for Single Reprints: Isabelle Boutron, MD, PhD, Equipe METHODS (CRESS-UMR1153), Centre d'épidémiologie clinique, Hôpital Hôtel-Dieu, 1 place du Parvis Notre-Dame, 75004 Paris, France.

Current author addresses and author contributions are available at Annals.org.

References

1. **Glasziou P, Altman DG, Bossuyt P, Boutron I, Clarke M, Julious S, et al.** Reducing waste from incomplete or unusable reports of biomedical research. Lancet. 2014;383:267-76. [PMID: 24411647] doi:10.1016/S0140-6736(13)62228-X
2. **Chalmers I, Glasziou P.** Avoidable waste in the production and reporting of research evidence. Lancet. 2009;374:86-9. [PMID: 19525005] doi:10.1016/S0140-6736(09)60329-9
3. **Begg C, Cho M, Eastwood S, Horton R, Moher D, Olkin I, et al.** Improving the quality of reporting of randomized controlled trials. The CONSORT statement. JAMA. 1996;276:637-9. [PMID: 8773637]
4. **Altman DG, Schulz KF, Moher D, Egger M, Davidoff F, Elbourne D, et al; CONSORT Group (Consolidated Standards of Reporting Trials).** The revised CONSORT statement for reporting randomized tri-

Downloaded from https://annals.org by Univ of North Carolina user on 08/13/2019

als: explanation and elaboration. Ann Intern Med. 2001;134:663-94. [PMID: 11304107] doi:10.7326/0003-4819-134-8-200104170-00012
5. **Moher D, Hopewell S, Schulz KF, Montori V, Gøtzsche PC, Devereaux PJ, et al.** CONSORT 2010 explanation and elaboration: updated guidelines for reporting parallel group randomised trials. BMJ. 2010;340:c869. [PMID: 20332511] doi:10.1136/bmj.c869
6. **Ceelen WP.** Clinical research in surgery: threats and opportunities. Eur Surg Res. 2014;53:95-107. [PMID: 25247239] doi:10.1159/000367606
7. **Evrard S, McKelvie-Sebileau P, van de Velde C, Nordlinger B, Poston G.** What can we learn from oncology surgical trials? Nat Rev Clin Oncol. 2016;13:55-62. [PMID: 26483296] doi:10.1038/nrclinonc.2015.176
8. **Boutron I, Tubach F, Giraudeau B, Ravaud P.** Methodological differences in clinical trials evaluating nonpharmacological and pharmacological treatments of hip and knee osteoarthritis. JAMA. 2003;290:1062-70. [PMID: 12941679]
9. **Boutron I, Guittet L, Estellat C, Moher D, Hróbjartsson A, Ravaud P.** Reporting methods of blinding in randomized trials assessing nonpharmacological treatments. PLoS Med. 2007;4:e61. [PMID: 17311468]
10. **Boutron I, Tubach F, Giraudeau B, Ravaud P.** Blinding was judged more difficult to achieve and maintain in nonpharmacologic than pharmacologic trials. J Clin Epidemiol. 2004;57:543-50. [PMID: 15246122]
11. **Pibouleau L, Boutron I, Reeves BC, Nizard R, Ravaud P.** Applicability and generalisability of published results of randomised controlled trials and non-randomised studies evaluating four orthopaedic procedures: methodological systematic review. BMJ. 2009;339:b4538. [PMID: 19920015] doi:10.1136/bmj.b4538
12. **Ergina PL, Cook JA, Blazeby JM, Boutron I, Clavien PA, Reeves BC, et al; Balliol Collaboration.** Challenges in evaluating surgical innovation. Lancet. 2009;374:1097-104. [PMID: 19782875] doi:10.1016/S0140-6736(09)61086-2
13. **Cook JA.** The challenges faced in the design, conduct and analysis of surgical randomised controlled trials. Trials. 2009;10:9. [PMID: 19200379] doi:10.1186/1745-6215-10-9
14. **McCulloch P, Altman DG, Campbell WB, Flum DR, Glasziou P, Marshall JC, et al; Balliol Collaboration.** No surgical innovation without evaluation: the IDEAL recommendations. Lancet. 2009;374:1105-12. [PMID: 19782876] doi:10.1016/S0140-6736(09)61116-8
15. **Boutron I, Moher D, Altman DG, Schulz KF, Ravaud P; CONSORT Group.** Extending the CONSORT statement to randomized trials of nonpharmacologic treatment: explanation and elaboration. Ann Intern Med. 2008;148:295-309. [PMID: 18283207] doi:10.7326/0003-4819-148-4-200802190-00008
16. **Boutron I, Moher D, Altman DG, Schulz KF, Ravaud P; CONSORT Group.** Methods and processes of the CONSORT Group: example of an extension for trials assessing nonpharmacologic treatments. Ann Intern Med. 2008;148:W60-6. [PMID: 18283201] doi:10.7326/0003-4819-148-4-200802190-00008-w1
17. **Schulz KF, Altman DG, Moher D; CONSORT Group.** CONSORT 2010 statement: updated guidelines for reporting parallel group randomised trials. PLoS Med. 2010;7:e1000251. [PMID: 20352064] doi:10.1371/journal.pmed.1000251
18. **Hopewell S, Clarke M, Moher D, Wager E, Middleton P, Altman DG, et al; CONSORT Group.** CONSORT for reporting randomised trials in journal and conference abstracts. Lancet. 2008;371:281-3. [PMID: 18221781] doi:10.1016/S0140-6736(07)61835-2
19. **Hopewell S, Clarke M, Moher D, Wager E, Middleton P, Altman DG, et al; CONSORT Group.** CONSORT for reporting randomized controlled trials in journal and conference abstracts: explanation and elaboration. PLoS Med. 2008;5:e20. [PMID: 18215107] doi:10.1371/journal.pmed.0050020
20. **Moore GF, Audrey S, Barker M, Bond L, Bonell C, Hardeman W, et al.** Process evaluation of complex interventions: Medical Research Council guidance. BMJ. 2015;350:h1258. [PMID: 25791983] doi:10.1136/bmj.h1258
21. **Blencowe NS, Blazeby JM, Donovan JL, Mills N.** Novel ways to explore surgical interventions in randomised controlled trials: applying case study methodology in the operating theatre. Trials. 2015;16:589. [PMID: 26710760] doi:10.1186/s13063-015-1127-x
22. **Hoffmann TC, Glasziou PP, Boutron I, Milne R, Perera R, Moher D, et al.** Better reporting of interventions: Template for Intervention Description and Replication (TIDieR) checklist and guide. BMJ. 2014;348:g1687. [PMID: 24609605] doi:10.1136/bmj.g1687
23. **Halm EA, Lee C, Chassin MR.** Is volume related to outcome in health care? A systematic review and methodologic critique of the literature. Ann Intern Med. 2002;137:511-20. [PMID: 12230353]
24. **Cook JA, Bruckner T, MacLennan GS, Seiler CM.** Clustering in surgical trials– database of intracluster correlations. Trials. 2012;13:2. [PMID: 22217216] doi:10.1186/1745-6215-13-2
25. **Roberts C, Roberts SA.** Design and analysis of clinical trials with clustering effects due to treatment. Clin Trials. 2005;2:152-62. [PMID: 16279137]
26. **Kahan BC, Morris TP.** Assessing potential sources of clustering in individually randomised trials. BMC Med Res Methodol. 2013;13:58. [PMID: 23590245] doi:10.1186/1471-2288-13-58
27. **Lee KJ, Thompson SG.** Clustering by health professional in individually randomised trials. BMJ. 2005;330:142-4. [PMID: 15649931]
28. **Roberts C, Walwyn R.** Design and analysis of non-pharmacological treatment trials with multiple therapists per patient. Stat Med. 2013;32:81-98. [PMID: 22865729] doi:10.1002/sim.5521
29. **Roberts C.** The implications of variation in outcome between health professionals for the design and analysis of randomized controlled trials. Stat Med. 1999;18:2605-15. [PMID: 10495459]
30. **Lee KJ, Thompson SG.** The use of random effects models to allow for clustering in individually randomized trials. Clin Trials. 2005;2:163-73. [PMID: 16279138]
31. **Wartolowska K, Judge A, Hopewell S, Collins GS, Dean BJ, Rombach I, et al.** Use of placebo controls in the evaluation of surgery: systematic review. BMJ. 2014;348:g3253. [PMID: 24850821] doi:10.1136/bmj.g3253
32. **Torgerson D.** Designing Randomised Trials in Health, Education and the Social Sciences. An Introduction. London: Palgrave Macmillan UK; 2008.
33. **Thornton JG, Hornbuckle J, Vail A, Spiegelhalter DJ, Levene M; GRIT study group.** Infant wellbeing at 2 years of age in the Growth Restriction Intervention Trial (GRIT): multicentred randomised controlled trial. Lancet. 2004;364:513-20. [PMID: 15302194]
34. **Burton AK, Tillotson KM, Cleary J.** Single-blind randomised controlled trial of chemonucleolysis and manipulation in the treatment of symptomatic lumbar disc herniation. Eur Spine J. 2000;9:202-7. [PMID: 10905437]
35. **Hoffmann TC, Erueti C, Glasziou PP.** Poor description of non-pharmacological interventions: analysis of consecutive sample of randomised trials. BMJ. 2013;347:f3755. [PMID: 24021722] doi:10.1136/bmj.f3755
36. **Nagendran M, Harding D, Teo W, Camm C, Maruthappu M, McCulloch P, et al.** Poor adherence of randomised trials in surgery to CONSORT guidelines for non-pharmacological treatments (NPT): a cross-sectional study. BMJ Open. 2013;3:e003898. [PMID: 24353256] doi:10.1136/bmjopen-2013-003898
37. **Baker M.** 1,500 scientists lift the lid on reproducibility. Nature. 2016;533:452-4. [PMID: 27225100] doi:10.1038/533452a
38. **Dworkin JD, McKeown A, Farrar JT, Gilron I, Hunsinger M, Kerns RD, et al.** Deficiencies in reporting of statistical methodology in recent randomized trials of nonpharmacologic pain treatments: ACTTION systematic review. J Clin Epidemiol. 2016;72:56-65. [PMID: 26597977] doi:10.1016/j.jclinepi.2015.10.019
39. **Yu J, Li X, Li Y, Sun X.** Quality of reporting in surgical randomized clinical trials. Br J Surg. 2017;104:296-303. [PMID: 27918069] doi:10.1002/bjs.10331
40. **McCleary N, Duncan EM, Stewart F, Francis JJ.** Active ingredients are reported more often for pharmacologic than non-pharmacologic interventions: an illustrative review of reporting practices in titles and abstracts. Trials. 2013;14:146. [PMID: 23688143] doi:10.1186/1745-6215-14-146

Downloaded from https://annals.org by Univ of North Carolina user on 08/13/2019

41. **Chan L, Heinemann AW, Roberts J.** Elevating the quality of disability and rehabilitation research: mandatory use of the reporting guidelines [Editorial]. Arch Phys Med Rehabil. 2014;95:415-7. [PMID: 24559651] doi:10.1016/j.apmr.2013.12.010
42. **Barnes C, Boutron I, Giraudeau B, Porcher R, Altman DG, Ravaud P.** Impact of an online writing aid tool for writing a randomized trial report: the COBWEB (Consort-based WEB tool) randomized controlled trial. BMC Med. 2015;13:221. [PMID: 26370288] doi:10.1186/s12916-015-0460-y
43. **Moher D, Schulz KF, Simera I, Altman DG.** Guidance for developers of health research reporting guidelines. PLoS Med. 2010;7:e1000217. [PMID: 20169112] doi:10.1371/journal.pmed.1000217

Annals Consult Guys: Learn and laugh with *Annals'* video-based CME feature.

Annals | Consult Guys

Annals Consult Guys bring a new perspective to the art and science of medicine with lively discussion and analysis of real-world cases and situations.

The videos are available to everyone, and ACP Members and *Annals* subscribers can earn .5 AMA PRA Category 1 Credit per video.

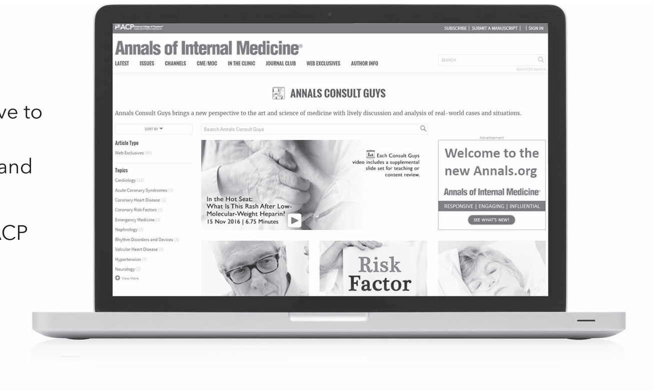

For more videos from and information on Annals Consult Guys, visit **www.annals.org/ConsultGuys.**

ACP American College of Physicians®
Leading Internal Medicine, Improving Lives

AIM6009

Downloaded from https://annals.org by Univ of North Carolina user on 08/13/2019

Current Author Addresses: Drs. Boutron and Ravaud: Equipe METHODS (CRESS-UMR1153), Centre d'épidémiologie clinique, Hôpital Hôtel-Dieu, 1 place du Parvis Notre-Dame, 75004 Paris, France.
Dr. Altman: Centre for Statistics in Medicine, Nuffield Department of Orthopaedics, Rheumatology and Musculoskeletal Sciences, University of Oxford, Botnar Research Centre, Windmill Road, Oxford OX3 7LD, United Kingdom.
Dr. Moher: Centre for Journalology, Ottawa Hospital Research Institute, The Ottawa Hospital, General Campus, 501 Smyth Road, Room L1288, Ottawa, Ontario K1H 8L6, Canada.
Dr. Schulz: FHI 360, 359 Blackwell Street, Suite 200, Durham, NC 27701.

Author Contributions: Conception and design: I. Boutron, D.G. Altman, D. Moher, K.F. Schulz, P. Ravaud.
Analysis and interpretation of the data: I. Boutron, D.G. Altman, D. Moher, P. Ravaud.
Drafting of the article: I. Boutron, D. Moher, P. Ravaud.
Critical revision of the article for important intellectual content: I. Boutron, D.G. Altman, D. Moher, K.F. Schulz, P. Ravaud.
Final approval of the article: I. Boutron, D.G. Altman, D. Moher, K.F. Schulz, P. Ravaud.
Statistical expertise: D.G. Altman, K.F. Schulz.
Obtaining of funding: P. Ravaud.
Administrative, technical, or logistic support: P. Ravaud.
Collection and assembly of data: I. Boutron, D. Moher, P. Ravaud.

Appendix 1: CONSORT NPT Group

The following members of the CONSORT NPT Group contributed to the article but did not author it:

Virginia Barbour (Office of Research Ethics & Integrity and Division of Research and Commercialisation, Queensland University of Technology, Brisbane, Queensland, Australia)

Kamaldeep Bhui (Centre for Psychiatry, Wolfson Institute of Preventive Medicine, Barts and the London School of Medicine and Dentistry, Queen Mary University of London, London, United Kingdom)

Nancy Chescheir (University of North Carolina at Chapel Hill Department of Obstetrics and Gynecology, Chapel Hill, North Carolina)

Pierre Alain Clavien (Department of Surgery, Swiss HPB and Transplantation Center, Department of Surgery, University Hospital Zürich, Zürich, Switzerland)

Markus K. Diener (Department of General, Visceral, and Transplantation Surgery; Study Center of the German Surgical Society, University of Heidelberg, Heidelberg, Germany)

Paul Glasziou (Centre for Research in Evidence Based Practice, Bond University, Robina, Queensland, Australia)

Robert M. Golub (*JAMA*)

Jeremy M. Grimshaw (Centre for Practice-Changing Research, Ottawa Hospital Research Institute; Department of Medicine, University of Ottawa, Ottawa, Ontario, Canada)

Trish Groves (*British Medical Journal*)

Sally Hopewell (Centre for Rehabilitation Research in Oxford, Nuffield Department of Orthopaedics, Rheumatology and Musculoskeletal Sciences, University of Oxford, Oxford, United Kingdom)

Sarah E. Lamb (Centre for Rehabilitation Research in Oxford, Nuffield Department of Orthopaedics, Rheumatology and Musculoskeletal Sciences, University of Oxford, Oxford, United Kingdom)

Peter McCulloch (Nuffield Department of Surgical Sciences, University of Oxford, Oxford, United Kingdom)

Cynthia Mulrow (*Annals of Internal Medicine*; University of Texas Health Science Center, San Antonio, Texas)

Daniel Riddle (Virginia Commonwealth University, Richmond, Virginia)

Joseph S. Ross (General Internal Medicine, School of Medicine and Health Policy and Management, School of Public Health, Yale University, New Haven, Connecticut)

Paula P. Schnurr (National Center for PTSD, White River Junction, Vermont; Geisel School of Medicine at Dartmouth, Hanover, New Hampshire)

David Schriger (University of California, Los Angeles, Los Angeles, California)

Larissa Shamseer (Centre for Practice-Changing Research, Ottawa Hospital Research Institute; School of Epidemiology, Public Health and Preventative Medicine, University of Ottawa, Ottawa, Ontario, Canada)

David Torgerson (York Trials Unit, Department of Health Sciences, University of York, York, United Kingdom)

Appendix 2: Literature Search

We searched for studies evaluating adherence to the CONSORT NPT extension and methodological developments that we should consider in the update. We searched Web of Science (search date, November 2013; update, July 2016) to identify all published articles citing 1 of the 2 articles on the 2008 CONSORT NPT extension (15, 16). We also searched MEDLINE via PubMed for studies evaluating the reporting of trials assessing NPTs since January 2009 (search date, November 2013; update, July 2016) by using the following search strategy: (Reporting AND CONSORT)[tiab], with a limitation to articles that had an abstract and were published in English. Finally, we used a personal collection of reports on new developments related to the specific methodological issues when assessing NPTs (for example, clustering within individual-patient RCTs or complexity of the intervention) and other relevant reporting guidelines developed since the publication of the 2008 CONSORT extension for NPTs, such as

Downloaded from https://annals.org by Univ of North Carolina user on 08/13/2019

the TIDieR checklist developed to report all types of interventions (22).

Web-Only References

44. **Frutos MD, Abrisqueta J, Lujan J, Abellan I, Parrilla P.** Randomized prospective study to compare laparoscopic appendectomy versus umbilical single-incision appendectomy. Ann Surg. 2013;257:413-8. [PMID: 23386239] doi:10.1097/SLA.0b013e318278d225

45. **Wyrsch B, McFerran MA, McAndrew M, Limbird TJ, Harper MC, Johnson KD, et al.** Operative treatment of fractures of the tibial plafond. A randomized, prospective study. J Bone Joint Surg Am. 1996;78:1646-57. [PMID: 8934478]

46. **Linehan MM, Comtois KA, Murray AM, Brown MZ, Gallop RJ, Heard HL, et al.** Two-year randomized controlled trial and follow-up of dialectical behavior therapy vs therapy by experts for suicidal behaviors and borderline personality disorder. Arch Gen Psychiatry. 2006;63:757-66. [PMID: 16818865]

47. **Molyneux A, Kerr R, Stratton I, Sandercock P, Clarke M, Shrimpton J, et al; International Subarachnoid Aneurysm Trial (ISAT) Collaborative Group.** International Subarachnoid Aneurysm Trial (ISAT) of neurosurgical clipping versus endovascular coiling in 2143 patients with ruptured intracranial aneurysms: a randomised trial. Lancet. 2002;360:1267-74. [PMID: 12414200]

48. **Bonjer HJ, Deijen CL, Abis GA, Cuesta MA, van der Pas MH, de Lange-de Klerk ES, et al; COLOR II Study Group.** A randomized trial of laparoscopic versus open surgery for rectal cancer. N Engl J Med. 2015;372:1324-32. [PMID: 25830422] doi:10.1056/NEJMoa1414882

49. **Zaidat OO, Fitzsimmons BF, Woodward BK, Wang Z, Killer-Oberpfalzer M, Wakhloo A, et al; VISSIT Trial Investigators.** Effect of a balloon-expandable intracranial stent vs medical therapy on risk of stroke in patients with symptomatic intracranial stenosis: the VISSIT randomized clinical trial. JAMA. 2015;313:1240-8. [PMID: 25803346] doi:10.1001/jama.2015.1693

50. **Lamb SE, Williamson EM, Heine PJ, Adams J, Dosanjh S, Dritsaki M, et al; Strengthening and Stretching for Rheumatoid Arthritis of the Hand Trial (SARAH) Trial Team.** Exercises to improve function of the rheumatoid hand (SARAH): a randomised controlled trial. Lancet. 2015;385:421-9. [PMID: 25308290] doi:10.1016/S0140-6736(14)60998-3

51. **Freedland KE, Carney RM, Rich MW, Steinmeyer BC, Rubin EH.** Cognitive behavior therapy for depression and self-care in heart failure patients: a randomized clinical trial. JAMA Intern Med. 2015;175:1773-82. [PMID: 26414759] doi:10.1001/jamainternmed.2015.5220

52. **Willett K, Keene DJ, Mistry D, Nam J, Tutton E, Handley R, et al; Ankle Injury Management (AIM) Trial Collaborators.** Close contact casting vs surgery for initial treatment of unstable ankle fractures in older adults: a randomized clinical trial. JAMA. 2016;316:1455-1463. [PMID: 27727383] doi:10.1001/jama.2016.14719

53. **Wiegersma M, Panman CM, Kollen BJ, Berger MY, Lisman-Van Leeuwen Y, Dekker JH.** Effect of pelvic floor muscle training compared with watchful waiting in older women with symptomatic mild pelvic organ prolapse: randomised controlled trial in primary care. BMJ. 2014;349:g7378. [PMID: 25533442] doi:10.1136/bmj.g7378

54. **Bronfort G, Hondras MA, Schulz CA, Evans RL, Long CR, Grimm R.** Spinal manipulation and home exercise with advice for subacute and chronic back-related leg pain: a trial with adaptive allocation. Ann Intern Med. 2014;161:381-91. [PMID: 25222385] doi:10.7326/M14-0006

55. **Friedli K, King MB, Lloyd M, Horder J.** Randomised controlled assessment of non-directive psychotherapy versus routine general-practitioner care. Lancet. 1997;350:1662-5. [PMID: 9400510]

56. **Rintoul RC, Ritchie AJ, Edwards JG, Waller DA, Coonar AS, Bennett M, et al; MesoVATS Collaborators.** Efficacy and cost of video-assisted thoracoscopic partial pleurectomy versus talc pleurodesis in patients with malignant pleural mesothelioma (MesoVATS): an open-label, randomised, controlled trial. Lancet. 2014;384:1118-27. [PMID: 24942631] doi:10.1016/S0140-6736(14)60418-9

57. **Droeser RA, Dell-Kuster S, Kurmann A, Rosenthal R, Zuber M, Metzger J, et al.** Long-term follow-up of a randomized controlled trial of Lichtenstein's operation versus mesh plug repair for inguinal hernia. Ann Surg. 2014;259:966-72. [PMID: 24169195] doi:10.1097/SLA.0000000000000297

58. **Van Buren G 2nd, Bloomston M, Hughes SJ, Winter J, Behrman SW, Zyromski NJ, et al.** A randomized prospective multicenter trial of pancreaticoduodenectomy with and without routine intraperitoneal drainage. Ann Surg. 2014;259:605-12. [PMID: 24374513] doi:10.1097/SLA.0000000000000460

59. **Feldon SE, Scherer RW, Hooper FJ, Kelman S, Baker RS, Granadier RJ, et al; Ischemic Optic Neuropathy Decompression Trial Research Group.** Surgical quality assurance in the Ischemic Optic Neuropathy Decompression Trial (IONDT). Control Clin Trials. 2003;24:294-305. [PMID: 12757995]

60. **Winstein CJ, Wolf SL, Dromerick AW, Lane CJ, Nelsen MA, Lewthwaite R, et al; Interdisciplinary Comprehensive Arm Rehabilitation Evaluation (ICARE) Investigative Team.** Effect of a task-oriented rehabilitation program on upper extremity recovery following motor stroke: the ICARE randomized clinical trial. JAMA. 2016;315:571-81. [PMID: 26864411] doi:10.1001/jama.2016.0276

61. **Guillou PJ, Quirke P, Thorpe H, Walker J, Jayne DG, Smith AM, et al; MRC CLASICC trial group.** Short-term endpoints of conventional versus laparoscopic-assisted surgery in patients with colorectal cancer (MRC CLASICC trial): multicentre, randomised controlled trial. Lancet. 2005;365:1718-26. [PMID: 15894098]

62. **Weeks JC, Nelson H, Gelber S, Sargent D, Schroeder G; Clinical Outcomes of Surgical Therapy (COST) Study Group.** Short-term quality-of-life outcomes following laparoscopic-assisted colectomy vs open colectomy for colon cancer: a randomized trial. JAMA. 2002;287:321-8. [PMID: 11790211]

63. **Foa EB, McLean CP, Capaldi S, Rosenfield D.** Prolonged exposure vs supportive counseling for sexual abuse-related PTSD in adolescent girls: a randomized clinical trial. JAMA. 2013;310:2650-7. [PMID: 24368465] doi:10.1001/jama.2013.282829

64. **Schnurr PP, Friedman MJ, Engel CC, Foa EB, Shea MT, Resick PM, et al.** Issues in the design of multisite clinical trials of psychotherapy: VA Cooperative Study No. 494 as an example. Contemp Clin Trials. 2005;26:626-36. [PMID: 16236558]

65. **Rogers CA, Pike K, Campbell H, Reeves BC, Angelini GD, Gray A, et al; CRISP investigators.** Coronary artery bypass grafting in high-RISk patients randomised to off- or on-Pump surgery: a randomised controlled trial (the CRISP trial). Health Technol Assess. 2014;18:v-xx, 1-157. [PMID: 25023641] doi:10.3310/hta18440

66. **Schnurr PP, Chard KM, Ruzek JI, Chow BK, Shih MC, Resick PA, et al.** Design of VA Cooperative Study #591: CERV-PTSD, comparative effectiveness research in veterans with PTSD. Contemp Clin Trials. 2015;41:75-84. [PMID: 25457792] doi:10.1016/j.cct.2014.11.017

67. **Costa ML, Achten J, Parsons NR, Rangan A, Griffin D, Tubeuf S, et al; DRAFFT Study Group.** Percutaneous fixation with Kirschner wires versus volar locking plate fixation in adults with dorsally displaced fracture of distal radius: randomised controlled trial. BMJ. 2014;349:g4807. [PMID: 25096595] doi:10.1136/bmj.g4807

68. **Duroy D, Boutron I, Baron G, Ravaud P, Estellat C, Lejoyeux M.** Impact of a computer-assisted Screening, Brief Intervention and Referral to Treatment on reducing alcohol consumption among patients with hazardous drinking disorder in hospital emergency departments. The randomized BREVALCO trial. Drug Alcohol Depend. 2016;165:236-44. [PMID: 27370526] doi:10.1016/j.drugalcdep.2016.06.018

69. **Cohen A, Assyag P, Boyer-Chatenet L, Cohen-Solal A, Perdrix C, Dalichampt M, et al; Réseau Insuffisance Cardiaque (RESICARD) PREVENTION Investigators.** An education program for risk factor management after an acute coronary syndrome: a randomized clinical trial. JAMA Intern Med. 2014;174:40-8. [PMID: 24126705] doi:10.1001/jamainternmed.2013.11342

70. **Taggart DP, Altman DG, Gray AM, Lees B, Nugara F, Yu LM, et al; ART Investigators.** Effects of on-pump and off-pump surgery in the Arterial Revascularization Trial. Eur J Cardiothorac Surg. 2015;47:1059-65. [PMID: 25217501] doi:10.1093/ejcts/ezu349

71. **Klaber Moffett JA, Jackson DA, Richmond S, Hahn S, Coulton S, Farrin A, et al.** Randomised trial of a brief physiotherapy intervention

Downloaded from https://annals.org by Univ of North Carolina user on 08/13/2019

compared with usual physiotherapy for neck pain patients: outcomes and patients' preference. BMJ. 2005;330:75. [PMID: 15585539]
72. **SoS Investigators.** Coronary artery bypass surgery versus percutaneous coronary intervention with stent implantation in patients with multivessel coronary artery disease (the Stent or Surgery trial): a randomised controlled trial. Lancet. 2002;360:965-70. [PMID: 12383664]
73. **Eker HH, Hansson BM, Buunen M, Janssen IM, Pierik RE, Hop WC, et al.** Laparoscopic vs. open incisional hernia repair: a randomized clinical trial. JAMA Surg. 2013;148:259-63. [PMID: 23552714] doi:10.1001/jamasurg.2013.1466
74. **Delitto A, Piva SR, Moore CG, Fritz JM, Wisniewski SR, Josbeno DA, et al.** Surgery versus nonsurgical treatment of lumbar spinal stenosis: a randomized trial. Ann Intern Med. 2015;162:465-73. [PMID: 25844995] doi:10.7326/M14-1420
75. **Neumayer LA, Gawande AA, Wang J, Giobbie-Hurder A, Itani KM, Fitzgibbons RJ Jr, et al; CSP #456 Investigators.** Proficiency of surgeons in inguinal hernia repair: effect of experience and age. Ann Surg. 2005;242:344-8. [PMID: 16135920]
76. **Shear K, Frank E, Houck PR, Reynolds CF 3rd.** Treatment of complicated grief: a randomized controlled trial. JAMA. 2005;293: 2601-8. [PMID: 15928281]
77. **Wright JG, Wang EE, Owen JL, Stephens D, Graham HK, Hanlon M, et al.** Treatments for paediatric femoral fractures: a randomised trial. Lancet. 2005;365:1153-8. [PMID: 15794970]
78. **Linde K, Streng A, Jürgens S, Hoppe A, Brinkhaus B, Witt C, et al.** Acupuncture for patients with migraine: a randomized controlled trial. JAMA. 2005;293:2118-25. [PMID: 15870415]
79. **Moseley JB, O'Malley K, Petersen NJ, Menke TJ, Brody BA, Kuykendall DH, et al.** A controlled trial of arthroscopic surgery for osteoarthritis of the knee. N Engl J Med. 2002;347:81-8. [PMID: 12110735]
80. **Hupperets MD, Verhagen EA, van Mechelen W.** Effect of unsupervised home based proprioceptive training on recurrences of ankle sprain: randomised controlled trial. BMJ. 2009;339:b2684. [PMID: 19589822] doi:10.1136/bmj.b2684
81. **Kutner JS, Smith MC, Corbin L, Hemphill L, Benton K, Mellis BK, et al.** Massage therapy versus simple touch to improve pain and mood in patients with advanced cancer: a randomized trial. Ann Intern Med. 2008;149:369-79. [PMID: 18794556] doi:10.7326/0003-4819-149-6-200809160-00003
82. **Keogh-Brown MR, Bachmann MO, Shepstone L, Hewitt C, Howe A, Ramsay CR, et al.** Contamination in trials of educational interventions. Health Technol Assess. 2007;11:iii, ix-107. [PMID: 17935683]
83. **Cook JA, Elders A, Boachie C, Bassinga T, Fraser C, Altman DG, et al.** A systematic review of the use of an expertise-based randomised controlled trial design. Trials. 2015;16:241. [PMID: 26025450] doi:10.1186/s13063-015-0739-5
84. **Devereaux PJ, Bhandari M, Clarke M, Montori VM, Cook DJ, Yusuf S, et al.** Need for expertise based randomised controlled trials. BMJ. 2005;330:88. [PMID: 15637373]
85. **Walter SD, Ismaila AS, Devereaux PJ; SPRINT Study Investigators.** Statistical issues in the design and analysis of expertise-based randomized clinical trials. Stat Med. 2008;27:6583-96. [PMID: 18837074] doi:10.1002/sim.3448
86. **Pike K, Angelini GD, Reeves BC, Taggart DP, Rogers CA.** Recruitment challenges in surgical trials: lessons from the CRISP trial. Trials. 2013;14(Suppl 1):P27. doi:10.1186/1745-6215-14-S1-P27
87. **Menon M, Muhletaler F, Campos M, Peabody JO.** Assessment of early continence after reconstruction of the periprostatic tissues in patients undergoing computer assisted (robotic) prostatectomy: results of a 2 group parallel randomized controlled trial. J Urol. 2008; 180:1018-23. [PMID: 18639300] doi:10.1016/j.juro.2008.05.046

Downloaded from https://annals.org by Univ of North Carolina user on 08/13/2019

Appendix Table 1. Characteristics of Corresponding Authors Surveyed About the CONSORT Statement Extension for RCTs of NPTs (*n* = 194)*

Characteristic	Value
Domain of expertise, %	
Surgery	23
Devices	4
Rehabilitation	24
Psychotherapy	8
Behavioral interventions	20
Other	21
Location, %	
Europe	54
United States	22
Canada	9
South America	3
Asia	3
Oceania	10
Mean RCTs participants had been involved in (SD), *n*	9.6 (30.5)
Mean reports of RCTs published (SD), *n*	3.8 (7.0)
Mean reports of RCTs evaluating NPTs published (SD), *n*	2.8 (3.7)
Use of CONSORT extension for NPT trials, %	40

CONSORT = Consolidated Standards of Reporting Trials; NPT = nonpharmacologic treatment; RCT = randomized controlled trial.
* Response rate was 13% (194 of 1525).

Appendix Table 2. Survey Results

Item	Item Number	Original CONSORT NPT Items	Participants Who Agreed to Retain the Item Without Modification (*n* = 194), %
Trial design	3a	How care providers were allocated to each trial group	89
Participants	4a	Eligibility criteria for centers and those performing the interventions	88
Interventions	5	Precise details of both the experimental treatment and comparator<br>5a. Description of the different components of the interventions and, when applicable, descriptions of the procedure for tailoring the interventions to individual participants<br>5b. Details of how the interventions were standardized<br>5c. Details of how adherence of care providers with the protocol was assessed or enhanced	72
Sample size	7a	Details of whether and how the clustering by care providers or centers was addressed	89
Blinding	11a	Whether or not those administering co-interventions were blinded to group assignment	81
Statistical methods	12a	Details of whether and how the clustering by care providers or centers was addressed	88
Participant flow	13a	The number of care providers or centers performing the intervention in each group and the number of patients treated by each care provider or in each center	84
Implementation of intervention	New item	Details of the experimental treatment and comparator as they were implemented	81
Baseline data	15	When applicable, a description of care providers (case volume, qualification, expertise, etc.) and centers (volume) in each group	88
Limitations	20	In addition, take into account the choice of the comparator, lack of or partial blinding, and unequal expertise of care providers or centers in each group	89
Generalizability	21	Generalizability (external validity) of the trial findings according to the intervention, comparators, patients, and care providers and centers involved in the trial	89

CONSORT = Consolidated Standards of Reporting Trials; NPT = nonpharmacologic treatment.

Downloaded from https://annals.org by Univ of North Carolina user on 08/13/2019

Appendix Table 3. Extended CONSORT Checklist of Information to Include When Reporting RCTs Assessing NPTs, With Examples for 2 Types of Interventions*

CONSORT Item and Extension for NPT Trials	Examples for RCTs Assessing Surgery and Procedures	Examples for RCTs Assessing Participative Interventions†
**Methods**		
Trial design		
Item 3a		
Standard CONSORT description<br>Description of trial design (such as parallel, factorial) including allocation ratio<br>Extension for NPT trials<br>When applicable, how care providers were allocated to each trial group	Surgery and procedures:<br>"This was a parallel group trial with a 1:1 allocation ratio. The same team of surgeons [. . .] performed both conventional laparoscopic appendectomy and single-port appendectomy." (44)<br>"Surgeons were assigned to a group on the basis of the operation that they preferred." (45)	Participative interventions:<br>Recruitment [. . .] of DBT [dialectical behavior therapy] Therapists<br>"There were 41 therapists in the study (16 DBT therapists and 25 CTBE therapists)." [. . .]<br>"Psychotherapists recommended by colleagues as potentially good DBT therapists were recruited for the study;" [. . .]<br>"The CTBE [Community Treatment by Experts] therapists were nominated by community mental health leaders. These included heads of inpatient psychiatric units and clinical directors of mental health agencies, who nominated therapists whom they considered experts in treating difficult clients." (46)
Participants		
Item 4a		
Standard CONSORT description<br>Eligibility criteria for participants<br>Extension for NPT trials<br>When applicable, eligibility criteria for centers and for care providers	Surgery and procedures:<br>"All participating centres [...] were major neurosurgical centres, treating large numbers of patients after aneurismal subarachnoid hemorrhage (SAH), each centre treating between 60 and 200 cases annually [...] Centres had to have expertise in both neurosurgical and endovascular management of ruptured aneurysms. Only accredited neurosurgeons with experience of aneurysm surgery were permitted to manage patients in the trial. Endovascular operators had to have done a minimum of 30 aneurysm treatment procedures, before they were permitted to treat patients in the trial." (47)<br>"The selection of centers for participation in the trial was based on stringent quality assessment by the study management committee to confirm the use of proper surgical technique. Unedited recordings of five consecutive laparoscopic total mesorectal excisions were evaluated. The respective pathology reports of these five consecutive cases were reviewed to confirm completeness of the specimens. Pathologists adhered to standardized processing and assessment of specimens, as described in detail in the trial protocol, to ensure accurate reporting by all participating centers. The circumferential resection margin was defined as "involved" when tumor cells were present within 2 mm from the lateral surface of the mesorectum." (48)<br>"To qualify as a study neurointerventionalist, physicians must have placed an intracranial stent in at least 10 patients (for aneurysm or atherosclerosis) in the 12 months prior to site initiation." (49)	Participative interventions:<br>"Therapists were trained to deliver both the exercise programme and control interventions. All therapists received 4 h of training covering theoretical and practical application of both interventions, and two short update training sessions during the trial. Therapists detailed the content of all treatment sessions in a standardised log book and clinical records. Every therapist received at least one quality-control assessment per intervention type, and all records were reviewed to ascertain attendance and for documentary evidence of assessment, and of progression or regression of exercises." (50)<br>"The cases were divided between 2 masters-level and 2 doctoral-level therapists, all of whom had prior training and experience with CBT for depression." (51)
Interventions		
Item 5		
Standard CONSORT description<br>The interventions for each group with sufficient details to allow replication, including how and when they were actually administered<br>Extension for NPT trials<br>Precise details of both the experimental treatment and comparator	See 5a, 5b, 5c	See 5a, 5b, 5c, 5d

Continued on following page

Downloaded from https://annals.org by Univ of North Carolina user on 08/13/2019

Appendix Table 3–Continued

CONSORT Item and Extension for NPT Trials	Examples for RCTs Assessing Surgery and Procedures	Examples for RCTs Assessing Participative Interventions†
Item 5a<br>Description of the different components of the interventions and, when applicable, description of the procedure for tailoring the interventions to individual participants.	Surgery and procedures:<br>"(. . .) participants were individually randomized to receive surgery or close contact casting (. . .)<br>Surgery was internal fixation conducted with internationally recognized principles and techniques.[12] Selection of implants, postoperative splinting, immediate or delayed weight bearing, and clinical follow-up were according to usual local practice and the surgeon's preference.<br>The close contact cast was applied in an operating room under general or spinal anesthesia by an orthopedic surgeon immediately after closed fracture reduction. Instructions were to achieve joint congruence with no talar shift or tilt. The close contact casting application was first a stockinette bandage (BSN Medical GmbH) and then shaped, self-adhesive foam pads (Fleecy Foam 5 mm; Hapla) placed over prominences (tibial crest, fibular head, calcaneum, Achilles tendon, and metatarsal heads) and medial and lateral sides of the ankle, where molding pressure was applied to hold the fracture reduction. The exact molding points for each participant were at the surgeon's discretion. Then 2 self-adhesive strips were applied to the full length of the cast (Fleecy web roll 5 cm; Hapla) to prevent plaster saw injury during removal. Finally, a single nonoverlapping synthetic wool layer (Soffban Plus; BSN Medical GmbH), plaster of paris (Gypsona; BSN Medical GmbH), and a reinforcing topcoat of synthetic casting material (Soft Cast Casting Tape; 3M Health Cate Ltd) were applied below the knee. All surgeons who applied casting had completed a 1-hour training session, supplemented with a video (https://www.youtube.com/playlist?list=PL2Gg_an4nwPflUC9RQV54Y2lb D76HiWcV) or were supervised by a surgeon who had completed training. Joint congruence was monitored with radiographs in the weeks after initial close contact cast application and after any reapplications for cast loosening. Reapplications did not require anesthesia. The protocol specified that if during clinical follow-up there was, in the treating surgeon's opinion, an unacceptable loss of fracture position before clinical union, he or she could remanipulate and reapply a cast in the outpatient clinic or operating room or convert to surgery. Guidance was that the casting group should touch or nonweight bear for 4 weeks and increase to full weight bearing by 6 to 8 weeks from intervention at the surgeon's discretion and patient volition.<br>The treatment protocol anticipated and allowed scenarios in which allocated treatment might have to be modified. Participants in the casting allocation could proceed to surgery when reduction could not be achieved or held with close contact casting in the operating room. Participants in the surgical allocation could proceed to traditional casting or external fixation when incision was considered unsafe, but not to close contact casting. For both allocations, a temporary treatment could be undertaken in the operating room (manipulation and splinting or external fixation) until it was appropriate to receive the allocated treatment. Each hospital followed its own protocols for thromboprophylaxis, surgical antibiotic prophylaxis, and rehabilitation." (52)	Participative interventions:<br>"[. . .]Pelvic floor muscle training was provided individually in face-to-face contacts combined with home exercises. The pelvic physiotherapists recorded the treatment modalities and the number of treatment sessions for each participant. For all participants, the intervention started with an explanation of the function of the pelvis and the pelvic floor and about pelvic floor dysfunctions; illustrations and three dimensional models of the pelvis were used. Pelvic floor muscle function was assessed by digital palpation. During this examination, the physiotherapists also checked whether participants were able to correctly contract ("squeeze and lift") and relax their pelvic floor muscles. If necessary, they used breathing exercises to increase awareness of the pelvic floor. Participants who were not able to contract or relax their pelvic floor muscles were first instructed how to do this by being given feedback during digital palpation or, if necessary, by application of myofeedback or electrical stimulation. Participants who were able to control their pelvic floor consciously but whose pelvic floor muscles were too weak started training their pelvic floor by doing exercises. All participants started with the same basic exercise scheme, to which specific exercises could be added (web **appendix 2**). The exercise programme was individualised and was modified at each appointment on the basis of examination findings. In cases of an overactive pelvic floor, the focus of the exercises was on relaxation rather than on contraction and, if necessary, general relaxation exercises were used. All participants were taught to contract their pelvic floor muscles before and during any increases in abdominal pressure ("the knack"), and attention was paid to lifestyle (diet, body weight) and toilet habits (web **appendix 2**). Initially, participants visited the pelvic physiotherapist on a weekly basis, but when they were able to correctly contract and relax their pelvic floor muscles the intervals between appointments were extended (two to three weeks). Participants were encouraged to continue practising at home three to five times a week, twice or three times each day." (53)<br>"The exercise programme was individualised and was modified at each appointment on the basis of examination findings. In cases of an overactive pelvic floor, the focus of the exercises was on relaxation rather than on contraction and, if necessary, general relaxation exercises were used." (53)<br>"The intervention protocols were developed and tested in previous pilot studies. Both interventions were intended to be pragmatic in nature (for example, modified to patient presentation and needs) and were informed by commonly recommended clinical practices, patient preferences, and promising research evidence." (54)<br>"Four therapists were employed for the study and had the necessary qualifications and experience to be accredited by the British Association for Counselling. They adhered to a Rogerian model of psychotherapy by helping the patient to express feelings, clarify thoughts, and to restate or reframe difficulties; the therapists also suggested alternative understandings. They did not give advice, but used empathy and advanced listening skills to help patients resolve their own difficulties. The therapists complied with a standard code of ethics and practice. Patients were offered six to 12 sessions of brief psychotherapy, which entailed 50 min of therapy once a week over 6 or more weeks. Patients randomised to the brief-psychotherapy intervention were able to see their general practitioners as usual. Therapists asked each patient to consent to the tape-recording of one session of brief psychotherapy. An independent therapist assessed the tapes for adherence to a non-directive, person-centred therapy style." (55)<br>Comparator:<br>"Participants randomised to watchful waiting received no treatment and no recommendations." (53)<br>"We chose routine general-practitioner care as the control approach. The doctors were able to talk to their patients and discuss their difficulties as they would do in normal practice. They were discouraged, however, from referring the patient to a therapist during the study period, unless absolutely necessary." (55)

Continued on following page

Downloaded from https://annals.org by Univ of North Carolina user on 08/13/2019

Appendix Table 3–Continued

CONSORT Item and Extension for NPT Trials	Examples for RCTs Assessing Surgery and Procedures	Examples for RCTs Assessing Participative Interventions†
Item 5b		
Details of whether and how the interventions were standardized.	Surgery and procedures:<br>"A detailed overview of the VAT-PP and talc pleurodesis techniques is described in the appendix. To ensure uniformity of approach, all surgeons discussed the VAT-PP procedure, and from Jan 13, 2009, they also recorded tumour extent and lung re-expansion before and after pleurectomy (appendix). At study outset, talc pleurodesis was done using talc slurry via an intercostal chest drain. From November, 2008, the protocol changed to allow talc pleurodesis by thoracoscopic poudrage." (56)<br>"All patients underwent standard repairs performed by residents, staff, or attending surgeons, following a detailed protocol. All surgeons participating in the study were personally instructed by the principal investigator and adequately trained in both surgical techniques." (57)<br>"For subjects assigned to the intraoperative drain group, the specific size, brand, and number of closed-suction drains were at the discretion of the surgeon." (58)<br>"The usual practices of surgeons performing optic nerve decompression surgery were determined through literature review and through a survey of study surgeons. These practices were described in the protocol as a series of 31 steps, only six of which were required to be performed so as to ensure adequacy of the surgery as well as safety of the patient. The remaining steps could be performed according to surgeon preference as they did not directly affect either patient safety or adequacy of surgery. Each study surgeon signed a written commitment to adhere to the six required steps, which were: general anesthesia, medial approach, no mechanical static traction, subarachnoid dissection if no cerebrospinal fluid release was seen following fenestration of the optic nerve sheath, no more than 7 minutes of sustained traction on the globe at any one time and rest periods of at least 2 minutes following any 7-minute period of globe traction." (59)	Participative interventions:<br>"The standardization process including initial training and maintenance throughout the trial is detailed in the Manual of Procedures (MOP). [. . .]<br>Training of all intervention therapists will occur during the initial six months of the study. The initial training activity will be led by the ASAP Intervention Team. ASAP intervention therapists (clinical site coordinators) will attend a 3-day training workshop in Los Angeles to accomplish Phase I competency in administration and documentation of a complete dose (30 hrs). For Phase II competency, each interventionist will be videoed, off-site during administration of each element (task-specific training; impairment mitigation; motivational enhancements) with study volunteers. Follow-up videotapes of the intervention therapist are required once a month for the first three months after the beginning of participant enrollment, once again three months later, and once every six months for the remainder of the project." (60)
Item 5c		
Details of whether and how adherence of care providers to the protocol was assessed or enhanced	Surgery and procedures:<br>"In every centre, a gastrointestinal pathologist ensured consistent reporting of resection specimens according to an agreed and established technique that focused on the completeness of resection and extent of the circumferential resection margin (CRM).[20] Tumour histology was reviewed centrally." (61)<br>"For surgical quality assurance, laparoscopic procedures performed on trial patients were videorecorded and members of the COST executive committee audited a randomly selected subset of the first 500 cases." (62)	Participative interventions:<br>"Counselors' adherence to treatment protocols was monitored via review of session videos during supervision meetings. Additionally, 20% of treatment sessions were randomly selected and rated by trained adherence raters who were otherwise uninvolved in the study. Raters assessed adherence to essential components of each treatment and monitored protocol violations." (63)<br>"All therapy sessions are videotaped [. . .]. A senior clinician who is independent of [. . .] treatment delivery will rate 10% of the videotapes using measures adapted from several randomized clinical trials of psychotherapy [. . .]; the 10% figure was chosen arbitrarily in an attempt to ensure an adequate sample of information from each treatment condition." (64)
Item 5d		
Details of whether and how adherence of participants to interventions was assessed or enhanced	Surgery and procedures: Not applicable	Participative interventions:<br>"Therapists detailed the content of all treatment sessions in a standardised log book and clinical records. Every therapist received at least one quality-control assessment per intervention type, and all records were reviewed to ascertain attendance and for documentary evidence of assessment, and of progression or regression of exercises. We defined patient compliance with the intervention as attendance at all face-to-face sessions with the therapist. Participants kept a diary record of exercise completion." (50)<br>"Treatment fidelity was facilitated through standardized training, manuals of operation, and clinical documentation forms that were monitored weekly by research staff." (54)

Continued on following page

Downloaded from https://annals.org by Univ of North Carolina user on 08/13/2019

Appendix Table 3–Continued

CONSORT Item and Extension for NPT Trials	Examples for RCTs Assessing Surgery and Procedures	Examples for RCTs Assessing Participative Interventions†
Sample size		
Item 7a		
Standard CONSORT description<br>How sample size was determined<br>Extension for NPT trials<br>Details of whether and how clustering by care providers or centers was addressed	Surgery and procedures:<br>"As all patients randomised to a given surgeon under expertise-based randomisation will have had their operations using the same technique, they cannot be regarded as independent of each other. Assuming that 80 surgeons would take part in the trial, the resultant intraclass correlation coefficient (ICC) was estimated from data from Bristol and Oxford cardiac databases to be 0.005. Using these assumptions, a sample size of 5418 patients had 90% power to detect a 30% reduction in RR with 5% significance (two tailed)." (65)	Participative interventions:<br>"The original sample size calculation did not include inflation for therapist effects, although we included assessment for these effects in the final analysis." (50)<br>"Although the treatment will be delivered on an individual basis, observations from the participants treated by the same therapist are likely to be correlated. Assuming each therapist treats *m* study participants, the sample size needs to be inflated by the following inflation factor *f* to retain the same power [56] and [57]: $f = 1 + (m - 1)\rho$, where ρ is the intraclass correlation due to therapist, or equivalently, the correlation between the primary outcomes from two individuals receiving treatment from the therapist. (. . .) The Planning Committee determined that it is reasonable to assume each therapist will deliver either PE or CPT to 8 participants over the course of the study ($m = 8$). It follows that $f = 1 + (8-1)*0.134 = 1.94$. Hence, a total of 878 participants (439 per group) is needed to provide 90% power to detect $\Delta\mu = 5$ in the primary outcome [. . .]" (66).
Blinding		
Item 11a		
Standard CONSORT description<br>If done, who was blinded after assignment to interventions (for example, participants, care providers, those assessing outcomes) and how<br>Extension for NPT trials<br>If done, who was blinded after assignment to interventions (for example, participants, care providers, those administering co-interventions, those assessing outcomes) and how	Surgery and procedures:<br>"The operating surgeon could not be blind in the trial and, as the Kirschner wires protrude on the back of the wrist and the locking plate require an incision, nor could the patient. All staff involved in checking, entering, and analysing questionnaire responses, however, were blind to allocation" (67)	Participative interventions:<br>"Participants were blinded to trial hypothesis. They were informed that the study aimed to compare two computer-assisted interventions in patients admitted to EDs, but they were not informed of all the research hypotheses. Furthermore, to limit bias, the data collection was standardized and performed by phone by independent and trained research assistants, who were blinded to the treatment allocation." (68)<br>"Using the standard double-blinding procedures employed in medication research is not feasible or desirable in psychotherapy research. Therapists need to be aware of which treatment they are delivering, and patients need to know as well. Blinded assessment is the gold standard in psychotherapy trials. Using centralized phone assessment for the primary outcome in this trial enhances blinding because assessors are not physically located where patients are receiving treatment, which offers an additional layer of protection from accidental unblinding. For secondary outcomes, the Site Coordinator collects patient self-report questionnaires by providing folders containing the questionnaire measures to participants and then collecting these folders from participants after completion." (66)
Item 11c		
Extension for NPT trials<br>If blinding was not possible, description of any attempts to limit bias	Surgery and procedures:<br>"A blinded health professional performed outcome assessments at the primary end point (6 months). Before assessments, opaque ankle bandages were applied to obscure the ankle. (. . .) The assessments at 6 weeks were not blinded because the assessor needed knowledge of postoperative instructions for weight bearing and movement. It was not possible to mask the surgeons or participants because of the nature of the interventions, nor was it possible to mask the radiograph assessors." (52)	Participative interventions:<br>"Blinding was not feasible in this study. However, independent research staff rather than the treating physician performed outcome assessments." (69)
Statistical methods		
Item 12a		
Standard CONSORT description<br>Statistical methods used to compare groups for primary and secondary outcomes<br>Extension for NPT trials<br>When applicable, details of whether and how clustering by care providers or centers was addressed	"We compared the on-pump and off-pump procedures for each baseline characteristic using a mixed-effect logistic regression model, fitting the group as the dependent variable and each baseline characteristic as the independent variable. We used an exchangeable covariance structure in the model to account for the clustering effect of surgeons. The analysis was stratified by allocated treatment group. Comparisons of the perioperative data between the two procedures were carried out using a generalized linear mixed-effect model. Similar adjustment for clustering of surgeons was included in the model." (70)	Participative interventions:<br>"We estimated therapist effects from a random effect nested within every centre." (50)<br>"Although the participants were individually randomized, a clustering of outcomes is potentially possible since a single therapist was treating several patients. If these clustering effects were strong, then this might alter the results. We therefore used multilevel modeling to check for any clustering effects by undertaking an analysis on the primary outcome." (71)<br>"The difference in the primary outcome between the intervention and comparator groups was evaluated at 12 months by a linear mixed longitudinal model estimating the difference in change from baseline between the 2 groups (coefficient for time × group interaction) and accounting for the correlation in data for the same patient and the same center (random effects)." (68)

Continued on following page

Downloaded from https://annals.org by Univ of North Carolina user on 08/13/2019

Appendix Table 3–Continued

CONSORT Item and Extension for NPT Trials	Examples for RCTs Assessing Surgery and Procedures	Examples for RCTs Assessing Participative Interventions†
**Results**		
Participant flow (a diagram is strongly recommended)		
Item 13a		
Standard CONSORT description<br>For each group, the number of participants who were randomly assigned, received intended treatment, and were analysed for the primary outcome<br>Extension for NPT trials<br>The number of care providers or centers performing the intervention in each group and the number of patients treated by each care provider or in each center	See Appendix Figure 1	See Appendix Figure 2
Item 13c		
Extension for NPT trials<br>For each group, the delay between randomization and the initiation of the intervention	Surgery and procedures:<br>"Patients meeting the clinical and angiographic criteria were randomly assigned 1:1 [. . .] The stent group underwent the stenting procedure within 48 hours of randomization." (49)<br>In a study comparing the effect of stent-assisted percutaneous coronary intervention (PCI) versus coronary artery bypass grafting (CABG) in the management of patients with multivessel disease (72): "The index procedure was done within 2 weeks of randomisation in 51% (n=247) of PCI patients and 33% (n=166) of surgery patients, and within 6 weeks in 94% (n=460) of PCI patients and 85% (n=422) of CABG patients. The median delay between randomisation and index procedure was 14 days (IQR 5–29) for PCI and 23 days (11–38) for CABG."	Participative interventions:<br>"The mean delay before commencement of manipulative treatment in the manipulator's office practice was 3 weeks (SD 3.6), whilst the mean delay for chemonucleolysis, performed at the hospital, was 12.9 weeks (SD 7.8)". (34)
New item		
Extension for NPT trials<br>Details of the experimental treatment and comparator as they were implemented	"Operative data for both groups are shown in Table 2. The mean operative time in the laparoscopic group was significantly longer than in the open group (76 minutes vs 100 minutes; P = .001). In the laparoscopic group, 8 of the 94 patients (8.5%) required conversion to open repair because of technical reasons. The estimated blood loss was significantly higher in the open group compared with the laparoscopic group (median, 50 mL vs 10 mL; P = .05). None of the patients required blood transfusion. Closed suction drains were placed subcutaneously in 45 patients in the open group and in the abdominal cavity in 3 patients in the laparoscopic group (P < .001)." (73)	Participative interventions:<br>"Overall, 94% of study patients attended their prescribed treatment visits: 98% in the SMT plus HEA group and 91% in the HEA group. The mean number of HEA visits was 3.8 (SD, 0.6; median, 4.0) in the SMT plus HEA group and 3.6 (SD, 1.0; median, 4.0) in the HEA group. The mean number of SMT visits was 14.6 (SD, 3.8; median, 16) in the SMT plus HEA group. Each HEA provider delivered care to approximately the same number of patients in each treatment group (range for SMT plus HEA group, 1 to 38; range for HEA group, 2 to 47); 7 chiropractors who delivered SMT plus HEA also delivered at least 1 HEA session. [. . .] There were no crossovers of treatment assignments during the trial." (54)<br>"Treatment Fidelity of physical therapy (PT). The average number of PT sessions attended was 8.4 (SD, 4.6). Fifty-four patients (66%) attended at least 50% of the prescribed 12 sessions. Thirteen patients (16%) did not attend at least 1 session, and 77% of them (*n* = 10) had surgery." (74)
Baseline data		
Item 15		
A table showing baseline demographic and clinical characteristics for each group<br>Extension for NPT trials<br>When applicable, a description of care providers (case volume, qualification, expertise, etc.) and centers (volume) in each group.	Surgery and procedures:<br>"We dichotomized surgeon's experience in laparoscopic repair into greater than 250 repairs (experienced) and less than 250 repairs (inexperienced) [...] Surgeons participating in this trial ranged in age from 27 to 70 with a median of 42 years in the laparoscopic group (55 surgeons) and from 30 to 76 with a median of 42 in the open group (77 surgeons). In the laparoscopic group, 8 surgeons were classified as experienced and 47 as inexperienced." (75)	Participative interventions:<br>"All therapists were master's- or doctoral-level clinicians who had at least 2 years of psychotherapy experience and who underwent extensive training and certification in either IPT or CGT. Certification entailed completion of 2 treatment cases in a manner judged competent by K.S. (for CGT) or E.F. (for IPT). Therapists received ongoing group supervision, separately for IPT and CGT, throughout the study period. Selected audiotapes or videotapes were used in supervision sessions as a part of the discussion. Therapy sessions were audiotaped for adherence and competence ratings, performed on a randomly selected subset of sessions." (76)

Continued on following page

Downloaded from https://annals.org by Univ of North Carolina user on 08/13/2019

Appendix Table 3–Continued

CONSORT Item and Extension for NPT Trials	Examples for RCTs Assessing Surgery and Procedures	Examples for RCTs Assessing Participative Interventions†
**Discussion**		
Limitations		
Item 20		
Standard CONSORT description<br>Trial limitations, addressing sources of potential bias, imprecision, and, if relevant, multiplicity of analyses<br>Extension for NPT trials<br>In addition, take into account the choice of the comparator, lack of or partial blinding, and unequal expertise of care providers or centers in each group	Surgery and procedures:<br>"First, surgeons might not be proficient in one or both treatments. The difference in malunion rates between the two treatment groups was consistent across all four study sites, indicating the difference is due to the procedure and not technical proficiency. Staff from all four centres were experienced in both techniques and, therefore, the results are probably typical of other paediatric centers." (77)	Participative interventions:<br>"The sham acupuncture intervention in our study was designed to minimize potential physiological effects by needling superficially at points distant from the segments of 'true' treatment points and by using fewer needles than in the acupuncture group. However, we cannot rule out that this intervention may have had some physiological effects. The nonspecific physiological effects of needling may include local alteration in circulation and immune function as well as neurophysiological and neurochemical responses. The question investigated in our comparison of acupuncture and sham acupuncture was not whether skin penetration matters but whether adherence to the traditional concepts of acupuncture makes a difference. For this purpose, our minimal acupuncture intervention was clearly an appropriate sham control although it might not be an inert placebo." (78)
Generalisability		
Item 21		
Standard CONSORT description<br>Generalizability (external validity, applicability) of the trial findings<br>Extension for NPT trials<br>Generalizability (external validity) of the trial findings according to the intervention, comparators, patients, and care providers and centers involved in the trial	Surgery and procedures:<br>"One surgeon performed all the procedures in this study. Consequently, his technical proficiency is critical to the generalisability of our findings. Our study surgeon is board-certified, is fellowship-trained in arthroscopy and sports medicine, and has been in practice for 10 years in an academic medical center. He is currently the orthopaedic surgeon for a National Basketball Association team and was the physician for the men's and women's U.S. Olympic basketball teams in 1996." (79)	Participative interventions:<br>"As the intervention was implemented for both sexes, all ages, all types of sports, and at different levels of sports, the results indicate that the entire range of athletes, from young elite to intermediate and recreational senior athletes, would benefit from using the presented training programme for the prevention of recurrences of ankle sprain. By including non-medically treated and medically treated athletes, we covered a broad spectrum of injury severity. This suggests that the present training programme can be implemented in the treatment of all athletes. Furthermore, as it is reasonable to assume that ankle sprains not related to sports are comparable with those in sports, the programme could benefit the general population." (80)

ASAP = Accelerated Skill Acquisition Program; CBT = cognitive behavior therapy; CGT = complicated grief treatment; CONSORT = Consolidated Standards of Reporting Trials; CPT = cognitive processing therapy; ED = emergency department; HEA = home exercise and advice; IPT = interpersonal psychotherapy; IQR = interquartile range; NPT = nonpharmacologic treatment; PE = prolonged exposure; RCT = randomized controlled trial; RR = relative risk; SMT = spinal manipulative therapy; VAT-PP = video-assisted thoracoscopic partial pleurectomy.
* We focused on these 2 types of interventions because they concern very different audiences.
† For example, rehabilitation or education.

Downloaded from https://annals.org by Univ of North Carolina user on 08/13/2019

Appendix Figure 1. Example of a participant flow diagram for a single-center trial of surgery.

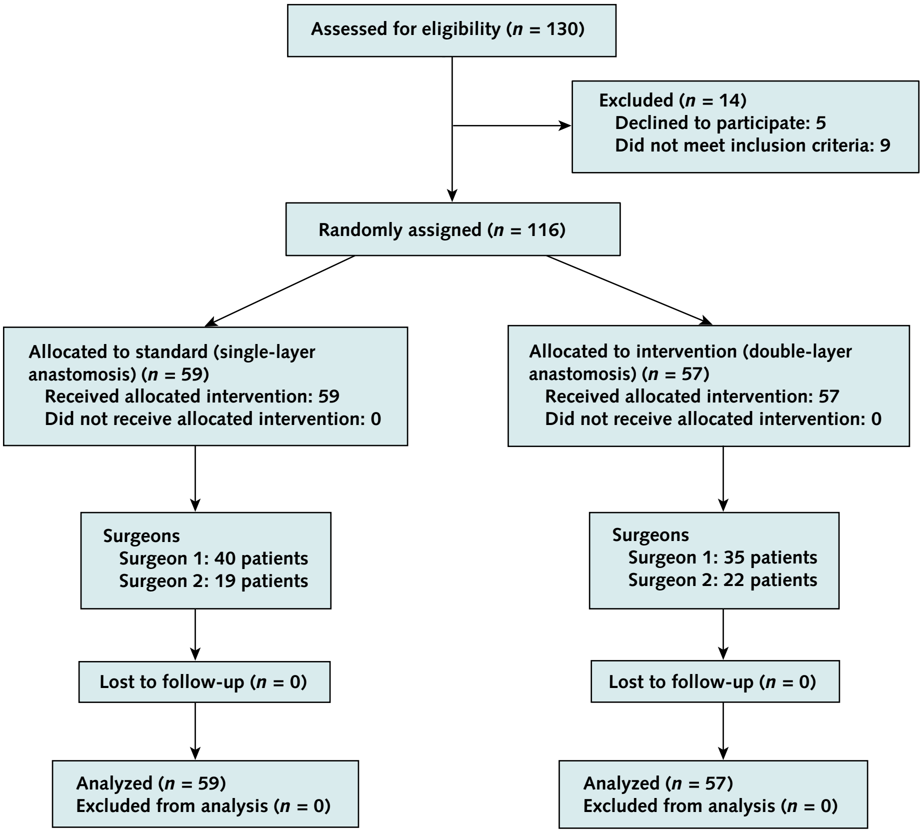

Adapted from reference 87.

Downloaded from https://annals.org by Univ of North Carolina user on 08/13/2019

Appendix Figure 2. Example of a participant flow diagram for a multicenter trial of participative interventions.

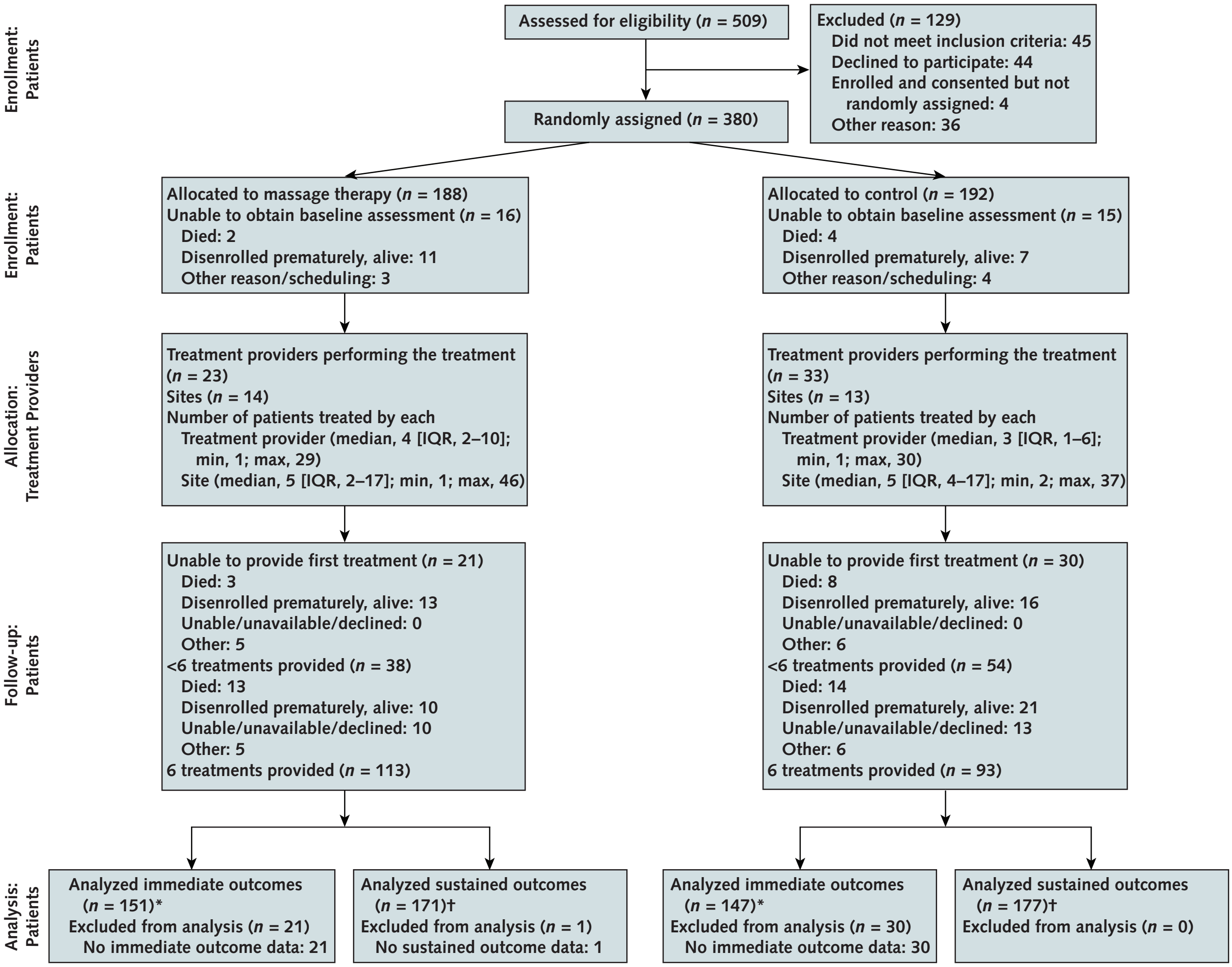

Assessments refer to the weekly or sustained outcomes. Immediate outcome data collection occurred in conjunction with every treatment session. IQR = interquartile range; max = maximum; min = minimum. Reproduced from reference 81, with permission.
* Number who had any treatment: 113 + 38 for massage therapy and 93 + 54 for control.
† Number with baseline or any sustained outcome assessments: 188 − 17 for massage therapy and 192 − 15 for control.

Downloaded from https://annals.org by Univ of North Carolina user on 08/13/2019

Appendix Table 4. Examples of Abstracts Adherent to the CONSORT Extension for Abstracts of NPT Trials*

**Title and Abstract of a Participative Intervention RCT Adherent to CONSORT NPT Abstracts**	**Title and Abstract of Surgical RCT Adherent to CONSORT NPT Abstracts**
Title: Massage therapy versus simple touch to improve pain and mood in patients with advanced cancer: a randomized trial (81).<br>Background: Small studies of variable quality suggest that massage therapy may relieve pain and other symptoms.<br>Objective: To evaluate the efficacy of massage for decreasing pain and symptom distress and improving quality of life among persons with advanced cancer.<br>Design: 2-arm parallel group single blind multisite, randomized clinical trial using a centralized computer generated randomization process. Only data collectors were blinded to treatment assignment (ClinicalTrials.gov: NCT00065195).<br>Setting: 15 U.S. hospices of the population-based Palliative Care Research Network.<br>Patients: 380 adults with advanced cancer who were experiencing moderate-to-severe pain (188 massage therapy, 192 control).<br>Intervention: Six 30-minute massage sessions **by licensed therapists with at least 6 months of experience** or simple-touch sessions **provided after a standardized training** over 2 weeks.<br>Measurements: Primary outcomes were immediate (Memorial Pain Assessment Card, 0- to 10-point scale) and sustained (Brief Pain Inventory [BPI], 0- to 10-point scale) change in pain over 3 weeks. Secondary outcomes were immediate change in mood (Memorial Pain Assessment Card) and 60-second heart and respiratory rates and sustained change in quality of life (McGill Quality of Life Questionnaire, 0- to 10-point scale), symptom distress (Memorial Symptom Assessment Scale, 0- to 4-point scale), and analgesic medication use (parenteral morphine equivalents [mg/d]). Immediate outcomes were obtained just before and after each treatment session. Sustained outcomes were obtained at baseline and weekly for 3 Weeks.<br>Results: 298 (151 massage therapy, 147 control) patients were included in the immediate outcome analysis and 348 (171 massage therapy, 177control) in the sustained outcome analysis. **A total of 82 patients did not receive any allocated study treatments (37 massage patients, 45 control patients).** Both groups demonstrated immediate improvement in pain (massage, −1.87 points [95% CI, −2.07 to −1.67 points]; control, −0.97 point [CI, −1.18 to −0.76 points]) and mood (massage, 1.58 points [CI, 1.40 to 1.76 points]; control, 0.97 point [CI, 0.78 to 1.16 points]). Massage was superior for both immediate pain and mood (mean difference, 0.90 and 0.61 points, respectively; $P < 0.001$). No between-group mean differences occurred over time in sustained pain (BPI mean pain, 0.07 point [CI, −0.23 to 0.37 points]; BPI worst pain, −0.14 point [CI, −0.59 to 0.31 points]), quality of life (McGill Quality of Life Questionnaire overall, 0.08 point [CI, −0.37 to 0.53 points]), symptom distress (Memorial Symptom Assessment Scale global distress index, −0.002 point [CI, −0.12 to 0.12 points]), or analgesic medication use (parenteral morphine equivalents, −0.10 mg/d [CI, −0.25 to 0.05 mg/d]). Adverse events were infrequent, were similar in both groups, and did not seem to be related to treatments.<br>Limitations: The immediate outcome measures were obtained by unblinded study therapists, possibly leading to reporting bias and the overestimation of a beneficial effect. The generalizability to all patients with advanced cancer is uncertain. The differential beneficial effect of massage therapy over simple touch is not conclusive without a usual care control group.<br>Conclusion: Massage may have immediately beneficial effects on pain and mood among patients with advanced cancer. Given the lack of sustained effects and the observed improvements in both study groups, the potential benefits of attention and simple touch should also be considered in this patient population.<br>Funding: National Institutes of Health and National Center for Complementary and Alternative Medicine (1R01AT01006-01A2), Mendel/Asarch Lung Cancer Family Foundation Grants Program.	Title: Surgery Versus Nonsurgical Treatment of Lumbar Spinal Stenosis: A Randomized Trial (74).<br>Background: Primary care management decisions for patients with symptomatic lumbar spinal stenosis (LSS) are challenging, and nonsurgical guidance is limited by lack of evidence.<br>Objective: To compare surgical decompression with physical therapy (PT) for LSS and evaluate sex differences.<br>Design: Multisite 2-arm parallel group randomized, controlled trial. (ClinicalTrials.gov: NCT00022776). Randomization was computer generated and concealed using sequentially numbered and sealed envelopes.<br>Setting: Neurologic and orthopedic surgery departments and PT clinics.<br>Participants: Surgical candidates with LSS aged 50 years or older who consented to surgery.<br>Intervention: Surgical decompression or PT. **All surgical procedures were performed by fellowship-trained spine surgeons or surgeons with more than 20 years of experience dedicated to spine surgery. Physical therapy was prescribed for 6 weeks, with a frequency of 2 visits per week, and was delivered by licensed physical therapists.**<br>Measurements: Primary outcome was physical function score on the Short Form-36 Health Survey at 2 years assessed by masked testers.<br>Results: The study took place from November 2000 to September 2007. A total of 169 participants were randomly assigned and stratified by surgeon and sex (87 to surgery and 82 to PT), with 24-month follow-up completed by 74 and 73 participants in the surgery and PT groups, respectively.<br>**All but 2 patients assigned to the surgery group received surgery. In contrast, 47 (57%) of the participants in the PT group crossed over to surgery over the 2-year period.** Mean improvement in physical function for the surgery and PT groups was 22.4 (95% CI, 16.9 to 27.9) and 19.2 (CI, 13.6 to 24.8), respectively. Intention-to-treat analyses revealed no difference between groups (24-month difference, 0.9 [CI, −7.9 to 9.6]). Sensitivity analyses using causal-effects methods to account for the high proportion of crossovers from PT to surgery (57%) showed no significant differences in physical function between groups. Thirty-three surgery-related complications occurred, 11 of which were in participants who crossed over from PT to surgery. All 9 PT-related complications were reports of worsening symptoms.<br>Limitation: Without a control group, it is not possible to judge success attributable to either intervention.<br>Conclusion: Surgical decompression yielded similar effects to a PT regimen among patients with LSS who were surgical candidates. Patients and health care providers should engage in shared decision-making conversations that include full disclosure of evidence involving surgical and nonsurgical treatments for LSS.<br>Primary Funding Source: National Institutes of Health and National Institute of Arthritis and Musculoskeletal and Skin Diseases.

CONSORT = Consolidated Standards of Reporting Trials; NPT = nonpharmacologic treatment; RCT = randomized controlled trial.
* Original abstracts were modified to adhere to the CONSORT extension for abstracts and the CONSORT extension for abstracts of NPT trials (CONSORT NPT abstracts). Information requested for CONSORT NPT abstracts is in boldface.

Downloaded from https://annals.org by Univ of North Carolina user on 08/13/2019

Appendix Table 5. Allocation of Care Providers to Each Trial Group

Allocation Across Group

In NPT trials where care providers could perform the intervention in both groups, the allocation of care providers to each trial group raises specific issues. Several situations are possible, all having advantages and drawbacks.

- Care providers deliver the intervention in both groups.
- In this situation, care providers may have to administer an intervention they are less confident with or expert in. Consequently, there is a risk for:
  - Low adherence to the protocol
  - Contamination (i.e., participants randomly assigned in 1 group inadvertently receive the intervention [or some component of the intervention] that is allocated to the other group) (82). Contamination may underestimate treatment effect estimates and reduce the study power.
  - Differential expertise bias (i.e., a disproportionate number of patients treated by an expert care provider in 1 group compared with the other group).
- Care providers could be randomly assigned to perform the intervention in 1 group.
- Care providers may be randomly assigned to an intervention they are not expert in or are less committed to. This situation would increase the risk for low adherence and contamination.
- Care providers could perform only the intervention they prefer or are expert in. Such choice is also called an "expertise-based randomized controlled trial" (83, 84). This design has several advantages. It should reduce the risk for contamination, take into account the learning curve and avoid the risk for differential expertise bias, and facilitate participation of care providers and patients. However, it also raises feasibility issues and questions the applicability of the trial results (85, 86).

Allocation Within Group

In some NPT trials, the care providers can perform the intervention in only 1 group (e.g., surgery vs. drug or surgery vs. physiotherapy). The allocation of care providers within groups is frequently determined by logistical considerations. However, in some situations, care providers could be randomly assigned among a pool of eligible care providers.

NPT = nonpharmacologic treatment.

Downloaded from https://annals.org by Univ of North Carolina user on 08/13/2019
