# Supplementary material for: Efficacy of supervised immersive virtual reality-based training for the treatment of chronic fatigue in post-COVID syndrome: study protocol for a double-blind randomized controlled trial (IFATICO Trial)
Source: Trials. 2024 Apr 3;25:232. doi: 10.1186/s13063-024-08032-w (PMC10993519; doi:10.1186/s13063-024-08032-w)
Supplement: Supplementary file 1 — Additional file 1. Ethical approval document. [file 13063_2024_8032_MOESM1_ESM.pdf]

University Hospital Heidelberg | Im Neuenheimer Feld 410 | 69120 Heidelberg

TRIALS

Victoria Vickerstaff, PhD

Editorial Office

Medical Hospital (Krehl-Klinik)
General Internal Medicine and Psychosomatics
Prof. Dr. med. H.-C. Friederich
Head of Department

Corresponding author Jonas Tesarz

Im Neuenheimer Feld 410
69120 Heidelberg, Germany
Tel 0049 /6221/56-37862
Fax 0049 /6221/56-5749
e-mail: jonas.tesarz@med.uni-heidelberg.de

Heidelberg, January 18th, 2024

Revision note for manuscript TRLS-D-23-00829

To the Editorial Board of TRIALS,

Dear Dr. Victoria Vickerstaff,

Thank you very much for reviewing our manuscript entitled: "Efficacy of supervised immersive Virtual Reality-based training for the treatment of chronic fatigue in Post-COVID Syndrome: Study protocol for a double-blind randomized controlled trial (IFATICO Trial)".

We are very pleased with the positive feedback from the reviewers, who describe our study as "a very promising and innovative intervention approach [...] to treat persistent fatigue associated with Covid-19" (Reviewer 2), state that it's a "clearly written protocol" (Reviewer 1) that addresses an "important and current topic" (Reviewer 2).

This respectful praise, together with the absolutely justified points of criticism and constructive comments, has greatly motivated us to incorporate them as fully as possible into the manuscript.

We have taken their suggestions to heart and have worked to create a more detailed description of our methodological considerations. This effort aims to make the conceptual framework more accessible and comprehensible, ensuring that our research findings are reproducible by the scientific community. As a result, we have revisited the presentation of our results and figures, refining the manner in which they are illustrated to enhance clarity and facilitate understanding.

In the attached response to reviewers, we describe point-by-point how we revised the manuscript according to each specific comment. Again, we are grateful for your invitation to revise our manuscript according to the reviewers' suggestions and are excited to continue working on it with you.

With kind regards,

Jonas Tesarz

Revision note for manuscript TRLS-D-23-00829:

"Efficacy of supervised immersive Virtual Reality-based training for the treatment of chronic fatigue in Post-COVID Syndrome: Study protocol for a double-blind randomized controlled trial (IFATICO Trial)"

Reviewer comments:

Responses to the comments of Reviewer #1:

This manuscript presents the protocol for a parallel group RCT of a VR mediated intervention vs active moderated exercise comparator for treating fatigue in people with long covid. This was a clearly written protocol, but lacking in some detail in some areas sufficient to allow replication. I also have some serious reservations about the validity of this trial due to the assumptions made in the sample size calculation, which I have explained in more detail below. My comments are structured in relation to adherence to individual items in the SPIRIT checklist.

- Thank you for praising our protocol as "clearly written" and also for pointing out some shortcomings in the description of the methodological details. Please find our point-by-point responses to your comments below.

Item 5b - The authors state that this item is n/a as the study was funded by the hospital that also features as the study site. However, funding and sponsorship are not the same thing. The study sponsor is the legal institution that takes responsibility for the conduct of the research. If this is the University Hospital Heidelberg, this should be explained here. There is a sponsor listed in the trial registration, so should also appear in this manuscript.

- Thank you for pointing this out - we are of course aware that funding and sponsorship are two completely different terms and that the responsible institution is acting as a sponsor here. We mistakenly overlooked this point in the SPIRIT checklist and have now corrected it. We have included a respective statement on the front page of our manuscript:

  Front page:

  "This study was conducted under the sponsorship of University Hospital Heidelberg, Germany. The sponsor had no role in the study design, data collection, management, analysis, interpretation of data, report writing, or in the decision to submit the report for publication".

Item 5d / Item 21a- the authors indicate that this is n/a in the SPIRIT checklist. This is not adequate. The authors need to include a statement in the manuscript that there are no oversight committees (trial steering committee or independent safety monitoring board) and the this.

- Thank you for highlighting this missing information. We have rewritten the section “Monitoring” to accommodate for this:

  Monitoring (p.36, 2nd para)

  “An independent Data Safety and Monitoring Board (DSMB) will be established for this trial to ensure the ongoing safety and integrity of the research. The DSMB will conduct regular assessments of the study at key intervals (baseline, 50% and 75% enrolment and at the end of recruitment and testing (last patient out)). The study will be overseen by the Principal Investigator (J. Tesarz), without the formation of a formal Scientific Study Steering Committee. This information has been included in the manuscript for full reporting of study governance.”

Item 11a - the description of how the participants will guided through the intervention or comparator exercise program is good- but there is still a lack of detail as to the type of activities they perform whilst using the ICARUS device or as part of the comparator exercise program. As it currently stands, I do not think the intervention or comparator programs could be replicated based on the information provided.

- Thank you for this useful comment, we fully agree that more specific description would be beneficial. We have therefore added a detailed description of the intervention and comparator program to the supplemental material of our manuscript, including screenshots of the games played on the ICAROS-device.

  Please find the rewritten training manuals attached.

It would also be useful to know what kind of trained specialists will be delivering the intervention/ comparator exercise program. Will they be physios/ research nurses/ occupation therapists or a mix of backgrounds? It would also be helpful to know if the same specialists delivering the VR intervention also deliver the comparator exercise program, or will these be different people.

- We agree that this information is of primary interest. The two training programs will be conducted by different personnel in order to maintain blinding and ensure treatment neutrality. Accordingly, specialists in the intervention group and comparison group will be different people who will be assigned their own patients and who will not switch between groups. The respective specialists (persons with a medical-therapeutic background, e.g. medical or psychology students or physiotherapists) will be trained by medical professionals on the respective training program as well as on medical information about post-COVID, on the difficulties of implementing

activation therapy in the treatment of post-COVID and on general issues in the treatment of patients with chronic diseases, and are regularly supervised. In the intervention arm, this specialist must have completed a training program by representatives of ICAROS ensuring safe and effective handling of the device. In the control arm, this specialist must have a completed training by study coordinates ensuring safe and effective execution of all exercises. Otherwise, the experts will not differ in the extent of their training and the information on the study between the two study arms. We have added this information to the manuscript:

Intervention integrity (p.16, 2nd para)

"Concerning intervention integrity, we consider criterion 1) to be fulfilled by the fact that all training sessions only take place under face-to-face supervision by a specialist. The two training programmes will be conducted by different personnel in order to maintain blinding and ensure treatment neutrality. Accordingly, specialists in the intervention group and comparison group will be different people who will be assigned their own patients and who will not switch between groups. The respective specialists (persons with a medical-therapeutic background, e.g. medical or psychology students or physiotherapists) will be trained by medical professionals on the respective training programme as well as on medical information about post-COVID, on the difficulties of implementing activation therapy in the treatment of post-COVID and on general issues in the treatment of patients with chronic diseases, and will be regularly supervised (2x2h introductory training, and every 4 weeks - adjust so that it corresponds approximately to the truth). In the intervention arm, this specialist must have completed a training program by representatives of ICAROS ensuring safe and effective handling of the device. In the control arm, this specialist must have a completed training by study coordinates ensuring safe and effective execution of all exercises. Otherwise, the experts will not differ in the extent of their training and the information on the study between the two study arms. ~~In the intervention arm, this specialist must have completed a training program by representatives of ICAROS ensuring safe and effective handling of the device."~~

Item 11c - the authors provide comprehensive detail as to how fidelity will be monitored in the intervention group- but there is no mention of how fidelity to the exercise program in the comparator group will be monitored. Please could this be included. If the monitoring methods described apply to both groups, please can this be stated.

- Thank you for this helpful comment! The monitoring methods apply to both groups and we have added respective statements in the "fidelity"-section to make this clearer:

Intervention integrity (p.15, last para and p.17, 2nd para)

"We consider criterion 2) to be met if a dose of a minimum of ten and a maximum of 18 training sessions (of a minimum of 15 and a maximum of 40 minutes) has taken place over a period of 4-8 weeks. We will assess the adherence to criterion 3) by requesting therapists in

both groups to fill in a self-assessment questionnaire at the end of each training session. This documents the content performed (type of exercises, including intensity, time, and number of repeats with a minimum number of 1 repeat), as well as any deviations from the treatment manual (see additional files 4 and 5). In the intervention arm, patients will additionally fill out the "Igroup Presence Questionnaire" assessing level of immersion (39, 40). Fidelity is met, if the level of immersion is above "0". We consider criterion 4) to be met, if patients report in 70% or more session that they remembered to include physical activity in their daily lives since the last session.

For further reference on intervention integrity, please find the fidelity checklist attached as additional file 3. This fidelity-checklist will be used to ensure intervention integrity in both the VR- and the comparison group."

Item 14 - This section is confusing. The authors state that this is an explorative study and so no formal sample size calculation was conducted, but then go on to describe a sample size calculation. They also state the this is an 'explorative study' but have confidently chosen a primary outcome and have seemingly determined a sample size based on power. This section needs to be re-written to be less contradictory.

I have some reservations about the assumptions chosen to determine the sample size as these do not appear to be wholly based in evidence and the power level is quite low. This very much feels like '100 participants' was picked as a sensible target and the sample size calculation reverse engineered to suit this. Conducting a trial that isn't adequately powered, based on broad assumptions could be considered unethical due to the high likelihood of finding no between group differences.

If the authors want to maintain that this is an 'exploratory' study, that is fine, but then it needs to be made clear that this is a pilot/ feasibility study and not powered to draw any conclusions.

- Thank you for your comment. When rereading the paragraph, we fully agree that it was confusing. We have comprehensively revised the derivation of our sample size calculation and clarified its presentation in the manuscript.

  In developing our study design, particularly in the absence of direct research on the therapeutic effect of immersive virtual reality-based neuromuscular training for post-COVID-associated fatigue, we consulted extensively with our biometrics department. In the absence of specific MCIC (minimal clinically important change) values for post-COVID fatigue measured by the FSS, we found the study by Scott Rooney et al., which examined MS patients, suggesting an MCIC range of 0.45–0.88 points on the FSS. We complemented this with data from a recent post-COVID intervention study (Jimeno-Almazán et al.), which observed a common mean FSS standard deviation of about 1.5 in treatment and control groups.

  We appreciate the feedback and have updated our manuscript to more clearly articulate these points, ensuring that our methodology and rationale are transparent and robustly supported.

Sample size calculation (page 19)

"The sample size calculation is based on testing the primary hypothesis that that immersive VR-based training therapy leads to a significantly different change in mean fatigue severity score (FSS) from baseline to follow-up assessment (T1) as compared to training therapy in the comparison arm. A difference of 0.45-0.88 points on the FSS is considered clinically relevant

(Rooney et al); we conservatively define the margin of 0.9 as the minimal clinically relevant difference for our trial. Based on previous post-COVID research (Jimeno-Almazán et al) we assume a common standard deviation of 1.5 among FSS- fatigue scores.

With a sample size of 45 in the experimental group (immersive neuromuscular training) and 45 in the control group (non-immersive physical activity training), a two-sided test for the difference between two means with a 0.05 significance level has 80% power to detect a difference of 0.9 when the standard deviation is 1.5. Assuming a dropout rate of 10%, we will randomize 100 participants, 50 to each condition. Sample size calculation was performed in PASS 16.0.12.''

References:

- Rooney S, McFadyen DA, Wood DL, Moffat DF, Paul PL. Minimally important difference of the fatigue severity scale and modified fatigue impact scale in people with multiple sclerosis. Mult Scler Relat Disord. 2019 Oct;35:158-163. doi: 10.1016/j.msard.2019.07.028. Epub 2019 Jul 28. PMID: 31400557.
- Jimeno-Almazán A, Franco-López F, Buendía-Romero Á, Martínez-Cava A, Sánchez-Agar JA, Sánchez-Alcaraz Martínez BJ, Courel-Ibáñez J, Pallarés JG. Rehabilitation for post-COVID-19 condition through a supervised exercise intervention: A randomized controlled trial. Scand J Med Sci Sports. 2022 Dec;32(12):1791-1801. doi: 10.1111/sms.14240. Epub 2022 Sep 23. PMID: 36111386; PMCID: PMC9538729

Item 17a - The authors state that the those delivering the intervention cannot be blinded, although they have taken steps to 'blind' the participants. The larger bias here is those conducting the outcomes assessments being blinded (or not) to the participant's allocation. However, I can find no information in the manuscript about the blinded status of the outcome assessors. Please can the authors include this information.

- Thank you for this comment, we fully agree, that blinding of outcome assessors should be discussed more up-front in the section “Masking and patient information” and have therefore rewritten the section.

Masking and patient information (p.21, first para)

“To minimize potential bias due to expectancy and reporting bias, this study is randomized-controlled and blinded in accordance with current recommendations for conducting nonpharmacological trials (22, 59). ~~By withholding information about the exact content of the interventions and the hypothesis of our study, we attempt to provide the best alternative to a double-blind nonpharmacologic intervention study.~~ Double-blinding encompasses firstly that subjects are not given insight into the content of the control study arm, and secondly that outcome assessors are not informed about what the study objective is and to which intervention arm participants are assigned. All outcome assessors are unaware of all patients group assignments. By also withholding information about the exact content of the interventions and the hypothesis of our study to patients, we attempt to provide the best alternative to a double-blind nonpharmacologic intervention study. Blinding of research staff conducting the intervention to this randomization is not possible. (..) All researchers who assess the outcomes or perform the data analyses will be masked as to group assignment. Patients will also be instructed not to discuss the content of their training program with other participant during the course of the study and may contact their trainer if they have any problems during study participation. In addition, we will instruct patients before the post-intervention interview not to mention which group, control, or intervention, they belonged to. In the case of unintentional unblinding during the assessment, the assessors will document how, and at which point the unblinding unfolded. Hence, we will be able to subsequently determine the extent to which blinded assessment was successful.”

Item18/19 - there is no indication as to how the data for the measures are collected. Are they paper based or using electronic data capture only? If on paper, how are these entered into the database? How is data quality maintained?

- This is another important concern, that we gladly addressed in our section “data collection”:

Data collection (page 23, 2nd para)

“We will collect participant data from intervention and comparison arms at baseline just prior to randomisation and at three- and six-months post intervention (see Figure 2 for the study schedule). We will use validated performance tasks and questionnaires and inform all participants that if they decide to withdraw from the study, the data already provided will be retained and used in the analyses unless they request otherwise and that a post-interventional assessment is planned even if they didn’t finish the study.

For the collection of patient-reported outcome measures as well as demographic and health data, we will use an electronic survey conducted in REDCAP. All data will automatically be updated to this database. For the collection of data from our assessments, we will use standardised paper-based checklists and sheets for all paper-based tasks. Data obtained from assessments will be manually uploaded to REDCAP. To ensure date quality, the data monitoring board will randomly choose patient files to assess whether data in REDCAP is identical to data in assessment-checklists and in sheets used for paper-based tasks."

Item 26a - the authors indicate that this is on p35 of the manuscript- but this refers to the consent for publication, not the SPIRIT item regarding who will consent participants to take part in the trial Please can the authors refer to the SPIRIT guidance and include this detail in the body of the manuscript

- We fully agree and have rewritten our section "data collection" to accommodate for this:

Data collection (page 22, last para)

"We will collect participant data from intervention and comparison arms at baseline just prior to randomisation and at three- and six-months post intervention (see Figure 2 for the study schedule). Informed verbal consent will be obtained from trial participants during our telephone screening by our study coordinates and informed written consent will be obtained by outcome assessors just prior to the pre-treatment assessment. Patients will be sent the consent form 24h before giving verbal consent and therefore more than 24h before giving written consent. We will use validated performance tasks and questionnaires and inform all participants that if they decide to withdraw from the study, the data already provided will be retained and used in the analyses unless they request otherwise and that a post-interventional assessment is planned even if they didn't finish the study."

Item 27 - this is not adequately detailed in the manuscript. There is a brief reference to pseudoanonymisation, but no detail on how this is achieved. There is also the issue of the collection of data from people who have not provided consent (see my comments re Figure 1 below). What will happen to the screening data collected on people that have not provided consent?

- Thank you for this important remark! We have rewritten our section "data management" to accommodate for this:

Data management (p.23)
"Responsibility for data management will be held by an independent data manager, which will not be involved in either the assessments or the delivery of therapies. Data are collected, managed, and stored in the central database under the supervision and responsibility of the data manager. The data manager ensures that all legal requirements for data protection are met and that data quality, sharing, and security (e.g., integrity of randomization) are maintained during all phases of the study. Data collected from patients after giving verbal but before giving written consent will be deleted if patients do not proceed to give written consent. In accordance with GCP guidelines, we ensure that all data and study documents are

pseudonymized and retained for at least 15 years after study completion. All participants will receive a code number as a pseudonym and all records that contain names or other personal identifiers will be stored separately."

Figure 1 - this is very blurry and difficult to read. I have a problem with data being collected form potential participants prior to obtaining their consent. Looking at the nature of the data collected in Figure 2 prior to informed consent, this includes personal and health data. I find it surprising that his has been approved by an ethics committee.

Thank you for stating this concern! We fully agree that this approach is unusual and shared your worry about patient safety. However, in Post-Covid-patients, every trip to the hospital is a great burden and we are very eager to minimize the number of hospital visits. We will educate patients about data protection in our telephone screening and obtain verbal consent before collecting personal and health related data. No data collected will be included in our analysis if we don't subsequently obtain written consent and all data of patients that no longer want to participate in the study after completing the survey will be deleted.
We apologize for the picture quality and have uploaded an Excel-sheet now called "Table 1"!

Figure 2 - this is also blurry and difficult to read.

- We apologize for the picture quality and have uploaded a Word-file now called "Figure 1"!

Responses to the comments of Reviewer #2:

The authors address an important and current topic. In the study protocol a very promising and innovative intervention approach is described to treat persistent fatigue associated with Covid-19.

- Thank you for praising our approach as a “very promising and innovative” study on an “important and current topic”. We have taken your suggestions to heart, please find a point-by-point response below.

Only a few points have to be addressed:

1. In the study protocol presented, the VR intervention is compared with an active control group. In addition, it would be beneficial to implement another control group such as a passive control group (e.g. waiting list) in line with the gold standard recommendations.

- We are happy to hear this suggestion as we also find that a passive control group would be beneficial to the quality of the study and in line with gold standard recommendations. However, we currently lack the resources to recruit for and supervise a passive control group. We are thinking about conducting another trial with a larger sample-size and a multicenter-approach, should the results of this trial be promising. If we do so, we will try to implement your suggestion.

2. In the methods section on page 10 from line 8 onwards, the authors point out that "immersion in virtual reality enables patients with limited physical capacity to experience joyful activities almost "fatigue-free", such as flying through the mountains in a wingsuit or moving weightlessly through space". However, this should already be mentioned in the "Background and rationale" section.

- Thank you for this helpful comment! We agree and have included these key points in our “Background and rationale” section on page 5:

  Background and rationale (p.6, 3rd para)
  “The virtual reality flight simulator is controlled by the participant's own body movement and thus conveys the feeling of flying. To promote participant motivation and engagement, the approach combines immersion with gamification, opening new challenges and game variations for participants. Patients with limited physical capacity can therefore experience joyful and “fatigue-free” physical activities linking the experience of joy to the experience to the experience of being physically active. ICAROS systems are used in sports, and rehabilitation training (19). Initial studies have shown that ICAROS training programs simultaneously develop reflexes and coordination skills and train different muscle groups.(20) The brain can thus gradually relearn its capabilities without having to exceed individual limits. So far, however, little is known about the impact of "virtual reality"-based fitness applications on individuals with Post-COVID associated conditions.”

3. The background section lacks a current estimate of the prevalence of persistent fatigue after Covid-19 infection, which makes it difficult for the reader to evaluate the significance of this important intervention approach ("Some of those infected experience long-term symptoms that can last for weeks to months" p. 3 l. 15).

- We have adopted this great suggestion and included a statement in our “Background” section: Background and rationale (p.4, first para)

  “Worldwide, hundreds of millions of people have become infected with the novel virus during the SARS-CoV-2 pandemic. Some of those infected experience long-term symptoms that can last for weeks to months and are often referred to as "Long-COVID " or "Post-COVID syndrome"(1, 2). According to current knowledge, the most common long-term symptoms are fatigue (3), poor concentration and shortness of breath, as well as a pronounced exercise intolerance ("Post-exertional malaise") (4, 5). The symptom-cluster “fatigue” (including “chronic fatigue” and “rapid physical exhaustion”) is the most reported symptom cluster 6 to 12 months after acute infection with an estimated prevalence of 37,2%. (Peter et. al)
  Affected persons who previously had no complaints, both physically and psychologically, suddenly find themselves confronted with substantial performance losses in both their mental and physical abilities (4, 5).”

  Reference: Peter RS, Nieters A, Krausslich HG, Brockmann SO, Gopel S, Kindle G, et al. Post-acute sequelae of covid-19 six to 12 months after infection: population based study. BMJ. 2022;379:e071050.

4. The subjects could participate in other intervention studies during the active intervention phase in the study (p. 13, line 9). This limits the interpretability of the results and should, if applicable, be included in the analysis as an important confounding variable or at least discussed critically.

- Thank you for this comment! We agree that participation in another intervention study would be an important confounder and have therefore included it in our section “exclusion criteria”. However, we agree that this should also be stated in our section on concomitant care and have therefore rewritten it:

  Concomitant care (p.15, first para)

  “The interventions are planned as an add-on to standard therapy. Prior to the trial, there are no restrictions regarding medication or other finished treatments. In both groups, participants may continue the treatment they received at baseline or start new therapies recommended by their treatment providers. Patients that participate in other intervention studies are however excluded from the study. For ~~exceptions~~ more detail~~-~~, see exclusion criteria.”

5. The subjects are encouraged to perform aerobic training at home in addition to the physical exercise intervention. This should be included as an important co-variable in the primary analysis in order to really estimate the specific effect of the intervention on the outcome variables, otherwise it is not possible to distinguish whether differences in the outcome variables are really attributable to the intervention or the confounder.

- Thank you for your insightful comment. We agree that the inclusion of home aerobic exercise as a covariate is critical to accurately assess the effect of the intervention on the outcome variables. To address this, we will include it as an additional sensitivity analysis and have updated the manuscript accordingly to reflect this improvement in our analytical approach.

  Statistical methods (p.34, last para)

  "In a sensitivity analysis, the per-protocol population is analyzed, which includes only patients in whose cases the integrity of the intervention (criteria 1-3) is met. Additionally, a complete case analysis will be performed meaning that the analysis of the primary endpoint is repeated in the subset with no missing values for the primary endpoint or relevant covariates. ~~In another sensitivity analysis, we will exclude unmasked patients from the analysis, to assess the impact of unmasking patients.~~ Furthermore, to evaluate the potential influence of at-home aerobic training, we will conduct an additional sensitivity analysis. This will involve adjusting for at-home aerobic training as a co-variable to discern its impact on outcome variables. Lastly, we will execute another sensitivity analysis where patients who were unmasked are excluded, assessing the effect of unmasking on the results."

6. Concerning the intervention integrity, the range in terms of session time from min. 15 minutes to max. 40 minutes can be classified as very high. A maximu m number of 18 sessions is also very generous and makes it difficult to compare the results. This should also be adjusted or at least discussed.

- Thank you for this important note. The range of sessions as well as treatment duration is indeed quite high. Since exhaustion levels in Post-COVID also differ greatly between patients and over time, we don't want patients to adhere to a strict schedule but to be able to lengthen or shorten session duration according to their pacing needs. However, we have adjusted the maximum number of sessions to 14 session and the session time to 45min with a range of 30 – 60min being acceptable in terms of fidelity.

Intervention integrity (p.15, last para and p.17, first para)

"We formulated the following core intervention components: 1) individual training sessions supervised by trained health specialists, 2) fixed dose of 2-3 training sessions per week of ~~30~~ 45 minutes each for each patient over a period of approximately six weeks

[...]

We consider criterion 2) to be met if a dose of a minimum of ten and a maximum of ~~18~~ 14 training sessions (of a minimum of ~~15~~ 30 and a maximum of ~~40~~ 60 minutes) has taken place over a period of 4-8 weeks. We will assess the adherence to criterion 3) by requesting therapists to fill in a self-assessment questionnaire at the end of each training session. This documents the content performed (type of exercises, including intensity, time, and number of repeats with a minimum number of 1 repeat), as well as any deviations from the treatment manual (see additional files 4 and 5). In the intervention arm, patients will additionally fill out the "Igroup Presence Questionnaire" assessing level of immersion (39, 40)."

Please find the rewritten fidelity checklist attached.

All modifications in the manuscript are marked in colour or ~~crossed out~~ using the track and change mode of MS word.
